# Novel *Para*‐Phenylenediamine‐Based Derivatives as Receptor Tyrosine Kinase‐like Orphan Receptor 1 (ROR1) Inhibitors: An In Vitro Preliminary Characterization

**DOI:** 10.1002/cmdc.202500247

**Published:** 2025-06-01

**Authors:** Gerardina Smaldone, Maria Rosaria Miranda, Francesca Di Matteo, Valeria Napolitano, Michela Aliberti, Simona Musella, Veronica Di Sarno, Gianluigi Lauro, Giuseppe Bifulco, Giacomo Pepe, Giovanna Aquino, Mario Felice Tecce, Isabel Maria Gomez‐Monterrey, Pietro Campiglia, Carmine Ostacolo, Alessia Bertamino, Vincenzo Vestuto, Tania Ciaglia

**Affiliations:** ^1^ Department of Pharmacy University of Salerno Via G. Paolo II 132, Fisciano 84084 Salerno Italy; ^2^ Department of Pharmacy University Federico II of Naples Via D. Montesano 49 80131 Naples Italy

**Keywords:** binding assays, cytotoxic effects, kinase inhibitors, novel chemotypes, synthesis

## Abstract

ROR1 kinase is an underexplored promising target for the development of novel anticancer drugs, being strongly expressed in several cancer cell lines, but poorly in non‐tumor cells. This property, together with the scarce number of molecules effective against ROR1, leads to the design and development of a research program aimed at the discovery of new chemical entities able to inhibit ROR1 thus interfering with its protumoral activity. Step‐by‐step in silico studies guide the design and synthesis of *para*‐phenylenediamine‐based compounds. Surface plasmon resonance and Cellular Thermal Shift Assay analyses, coordinated with cytotoxicity assays carried out on JeKo‐1 (mantle cell lymphoma) and SH‐SY5Y (neuroblastoma cell) cell lines, demonstrate the strong affinity and the anticancer potential of the derivative **17**, respectively, further confirming its mechanism of action. Moreover, pharmacokinetic assessment reveals a good stability profile for derivative **17**, paving the way for additional SAR studies on the *para*‐phenylenediamine as a scaffold for developing new ROR1 inhibitors.

## Graphical abstract

ROR1 represents a promising target for the development of novel antiproliferative compounds, giving its high expression in different cancer cell lines. The present study describes the workflow leading to the design, synthesis, and characterization of a series of *para*‐phenylenediamine‐based compounds able to interact with the target kinase, inhibiting its pro‐tumor effect.

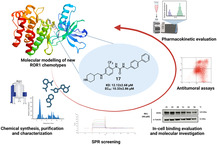

## 1 Introduction

ROR1 (Receptor tyrosine kinase‐like Orphan Receptor 1), reported as an orphan receptor for a long time, has been recently identified as target of Wnt signaling,^[^ ^1^ ^]^ which is an ancient and highly conserved family of proteins playing a pivotal role in embryonic development and adult tissue homeostasis.^[^ ^2^ ^]^ Wnt consists of canonical and noncanonical pathways, mediating different biological effects via binding to Frizzled receptors.^[^ ^2^, ^3^, ^4^ ^]^ Along with the binding of the noncanonical factor Wnt5a to ROR1, Ser, and Tyr residues phosphorylation, homo‐ and/or heterodimerization with the homologous protein ROR2 can occur,^[^ ^5^ ^]^ leading to the activation of several signaling transduction pathways, such as NF‐κB,^[^ ^1^ ^]^ Rho/Rac1 GTPases,^[^ ^6^ ^]^ PI3K/AKT/mTOR,^[^ ^7^ ^]^ JAK/STAT, and Hippo,^[^ ^8^ ^]^ favoring prosurvival mechanisms in multiple cancer cell lines,^[^ ^9^ ^]^ and explaining the growing interest of the scientific community in the protein activity. The increased attraction in the pseudo‐kinase functionality can be further explained by its peculiar expressional behavior: ROR1 is an embryonic protein, missing in most tissues during adult life, while it is highly expressed in several tumor types, including chronic lymphocytic leukemia (CLL), mantel cell lymphoma (MCL), large B‐cell lymphoma (DLBCL), pancreatic, ovarian, endometrial and breast cancers.^[^ ^10^, ^11^, ^12^, ^13^, ^14^, ^15^, ^16^ ^]^ The structure of the transmembrane protein ROR1 resembles that of proteins belonging to the receptor tyrosine kinases (RTK) family, with an extracellular immunoglobulin‐like (*I*g) domain, a Frizzled domain, rich in cysteine residues, and a Kringle domain. The intracellular side contains the catalytic moiety (aa 473–746) and two Ser/Thr domains, interspersed with a Pro‐rich domain.^[^ ^17^ ^]^ ROR1 is classified as pseudo kinase, because it is devoid of catalytic activity and consequently of the catalytic aminoacidic triad in its kinase domain, and its exact mechanism of action remains still ambiguous.^[^ ^18^ ^]^ ROR1 displays an autophosphorylation activity; however, it has been demonstrated to be highly *trans*‐phosphorylated by other kinases, including Met, Src, and Lyn.^[^ ^19^, ^20^ ^]^ Y641, Y656, and Y646, located in ROR1 pseudo‐kinase domain, are among the amino acids exposed to the *trans*‐phosphorylation reaction.

**Figure 1:**
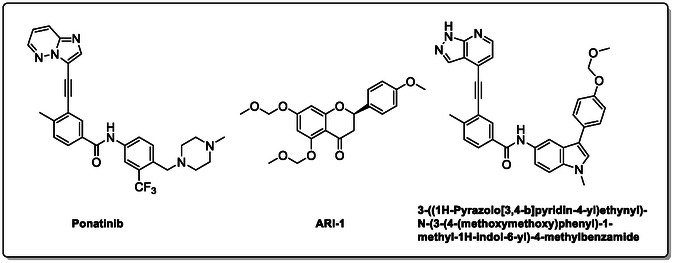
Known chemical structures of ROR1 inhibitors.

In this last decade, scientific interest has been addressed toward ROR1 because of its critical role in tumor development. In recent years, it has been demonstrated that by blocking ROR1 signaling, the response to chemotherapy results improved,^[^ ^21^ ^]^ while cell proliferation decreases in patients with breast cancer.^[^ ^22^ ^]^ The positive effect of ROR1 inhibition/silencing has been further demonstrated in lung cancer cells,^[^ ^23^ ^]^ chemo‐resistant ovarian cancer cells,^[^ ^24^ ^]^ and lymphocytic leukemia cells.^[^ ^25^, ^26^, ^27^, ^28^ ^]^ Despite the growing interest in ROR1 kinase pivotal role in cancer progression, just a monoclonal antibody, cirmtuzumab, reached clinical trial and, overall, a very reduced number of molecules demonstrated to act as ROR1 inhibitors in vitro, such as ARI‐1 and the 3‐((1 H‐pyrazolo[3,4 ‐ b]pyridin‐4‐yl)ethynyl)‐N‐(3‐(4‐(methoxymethoxy)phenyl)‐1‐methyl‐1 H‐indol‐6‐yl)‐4‐methylbenzamide (**Figure** **1**).^[^ ^23^, ^29^, ^30^ ^]^ The best characterized small molecules interacting with ROR1 is ponatinib, the 3‐(2‐imidazo[1,2‐*b*]pyridazin‐3‐ylethynyl)‐4‐methyl‐*N*‐[4‐[(4‐methylpiperazin‐1‐yl)methyl]‐3‐(trifluoromethyl)phenyl]benzamide (Figure 1), a pan tyrosine kinase inhibitor for which the cocrystallized structure is deposited in the Protein Data Bank (PDB code: 6TU9).^[^ ^31^ ^]^ Here, molecular modeling experiments were carried out using ROR1 crystal structure, leading to the design and synthesis of a series of *para*‐phenylenediamine‐based derivatives as potential ROR‐1 inhibitors. The initial exploration of ROR1 binding pocket has been driven by a structure‐based approach combined with synthetical considerations, derived from chemical accessibility and suitability to generate chemical diversity. In this regard, the phenylenediamine scaffold ensures two different functionalization sites expanding the knowledge of the interaction area within the protein binding pocket, and for this reason, it has been chosen as the starting core for our preliminary investigation. Therefore, in this article, we describe the process leading to the development of novel chemical entities acting as ROR1 ligands and further characterized for their biological activity. The synthesized compounds were first analyzed for the direct binding to ROR1 kinase domain by using surface plasmon resonance (SPR) assays, selecting the most promising molecules for further cellular studies. Cytotoxicity was then assessed through MTT assays in two different cell lines, SH‐SY5Y and JeKo‐1, revealing a stronger antiproliferative effect in JeKo‐1 cells. Given this higher sensitivity, we focused on JeKo‐1 to further investigate apoptotic effects, where Annexin V/PI staining confirmed a significant induction of apoptosis upon treatment. To deepen our understanding of the compound interaction with ROR1, we performed a Cellular Thermal Shift Assay (CETSA), which demonstrated clear stabilization of ROR1 in the presence of the inhibitor, confirming a direct target engagement. Finally, western blot analysis revealed that treatment with our derivatives led to a marked reduction in ROR1 phosphorylation, impacting downstream signaling pathways and promoting proapoptotic factors, further supporting the compound mechanism of action. Moreover, a preliminary in vitro pharmacokinetic study highlighted high stability for compound 17, supporting its potential for further investigations. Collectively, these findings establish 17 as a novel chemotype effective against ROR1 and pave the way for further progress in the identification of novel ROR1 inhibitors. Although kinase selectivity profiling is a valuable aspect of kinase inhibitor characterization, it was not performed at this stage for compound 17. As it represents an initial hit identified against a relatively underexplored target, and showed promising engagement and cellular activity in the mid‐micromolar range, our priority was to validate its potential for further optimization. Broader selectivity profiling will be considered in future studies as part of the hit‐to‐lead development process.

## 2 Results and Discussion

### 2.1 Chemistry

**Scheme 1:**
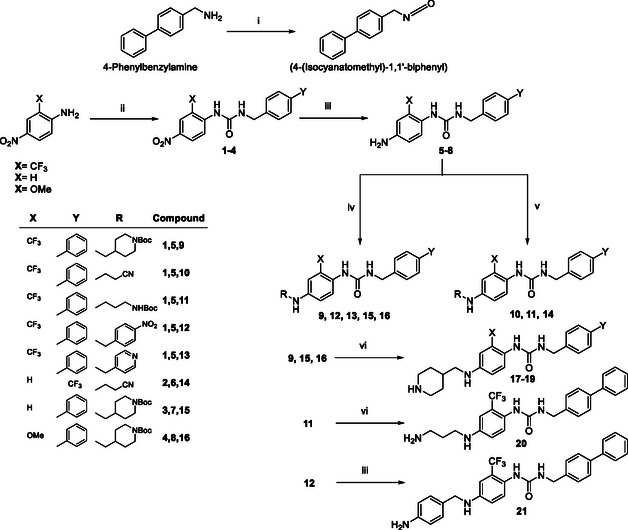
Synthesis of derivatives **10**, **13**, **14**, and **17**–**21**. Reagents and conditions: i) Cl_3_COCOOCCl_3_, TEA, DCM, 60 °C, 12 h; ii) (4‐(Isocyanatomehtyl)‐1,1′‐biphenyl) or 4‐(CF_3_)phenyl isocyanate, *tert*‐BuOK, ACN, 100 °C, 12 h; iii) HCO_2_NH_4_, Pd/C, MeOH/THF, 100 °C, 1–2 h; iv) R‐CHO, CHCl_3_, 50 °C, 12 h, then NaBH_4_, rt, 1 h; v) RBr, *tert*‐BuOK, ACN, MW, 120 °C, 1 h; vi) DCM/TFA (3/1, v/v), TIS, rt, 3 h.

Derivatives **10**, **13**, **14**, and **17–21** were synthesized as depicted in **Scheme** **1**. 4‐Nitro‐2‐(trifluoromethyl)aniline, 4‐nitroaniline, or 2‐methoxy‐4‐nitroaniline was reacted with (4‐(Isocyanatomethyl)‐1,1′‐biphenyl) in acetonitrile using potassium *tert*‐butoxide as base, furnishing the urea intermediates **1**, **3**, and **4** (yield 39–62%). (4‐(Isocyanatomethyl)‐1,1′‐biphenyl) was previously obtained from 4‐phenylbenzylamine and triphosgene in dichloromethane as solvent. The same synthetic approach led to intermediate **2** in 42% yield starting from 2‐trifluoromethyl‐4‐nitro aniline and the commercially available 4‐(trifluoromethyl)phenyl isocyanate. The reduction of the nitro group employing ammonium formate as hydrogen donor and Pd/C as catalyst in a mixture of MeOH/THF led to the amino intermediates **5**–**8** (yield 75–92%). Compounds **5**, **7**, and **8** were subjected to a reductive amination in the presence of 1‐Boc‐piperidine‐4‐carboxaldehyde, 4‐nitrobenzaldehyde, or 4‐pyridinecarboxaldehyde and sodium borohydride as reducing agents generating the trisubstituted compounds **9**, **12**, **13**, **15**, and **16** (yield 51–58%). Intermediates **5** and **6** were further reacted in basic medium under microwave irradiation with 3‐bromopropionitrile, affording, respectively, final compounds **10** and **14** (yield 43–48%), while the reaction of intermediate **5** with 3‐(Boc‐amino)propyl bromide yielded intermediate **11** (yield 43%). N‐Boc removal from intermediates **9**, **11**, **15**, and **16**, in DCM/TFA 3/1 as ratio, using triisopropylsilane as scavenger, gave final derivatives **17**–**20** almost quantitatively, while the reduction of the nitro group of intermediate **12**, under the conditions described above, allowed to obtain derivative **21** in 77% of overall yield.

**Scheme 2:**
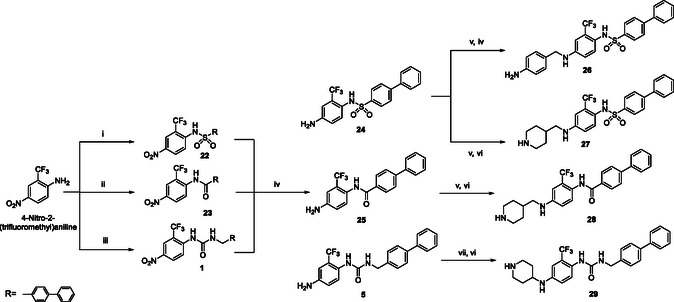
Synthesis of derivatives **26**‐**29**. Reagents and conditions: i) Biphenyl‐4‐sulfonyl chloride, NaH, THF, 90 °C, 12 h; ii) Biphenyl‐4‐carbonyl chloride, DBU, DCM, 45 °C, 12 h; iii) (4‐(Isocyanatomethyl)‐1,1′‐biphenyl), *tert*‐BuOK, ACN, 100 °C, 12 h; iv) HCO_2_NH_4_, Pd/C, MeOH/THF, 100 °C, 1 h; v) 1‐Boc‐piperidine‐4‐carboxaldehyde, CHCl_3_, 50 °C, 12 h then NaBH_4_, rt, 1 h; vi) DCM/TFA (3/1, v/v), TIS, rt, 3 h; vii) 1‐Boc‐4‐bromopiperidine, *tert*‐BuOK, ACN, MW, 120 °C, 1 h.

Final compounds **26–29** were synthesized in accordance with **Scheme** **2**. Treatment of 4‐nitro‐2‐(trifluoromethyl)aniline with biphenyl‐4‐sulfonyl chloride, biphenyl‐4‐carbonyl chloride, or 4‐(isocyanatomethyl)‐1,1′‐biphenyl under basic conditions led, respectively, to intermediates **22**, **23** and **1** (yield 39–47%). The following reduction reaction, applying the previously reported conditions, furnished compounds **24**, **25**, and **5** in 73–84% of yield. Intermediates **24** and **25** were subjected to a reductive amination reaction employing 1‐Boc‐piperidine‐4‐carboxaldehyde and a subsequent Boc deprotection afforded final derivatives **27** and **28** in 35% and 44% of overall yields, respectively. Compound **24** was also reacted with 4‐nitrobenzaldehyde, generating the correspondent Schiff base, which was reduced using NaBH_4_ and finally converted in the amino derivative **26** (52% of yield). Final compound **29** was obtained in 36% of yield from the reaction between **5** and 1‐Boc‐4‐bromopiperidine under microwave irradiation, and subsequent Boc removal in the conditions described above.

### 2.2 In Silico Studies and Biophysical Assays

Computational studies were performed for the step‐by‐step rational design of new compounds capable of interfering with the activity of ROR1 through interaction with its pseudo‐kinase domain. We performed molecular docking experiments to explore the binding mode of novel derivatives using the crystallographic structure of the protein co‐complexed with ponatinib, a known inhibitor of ROR1 (PDB code: 6TU9).^[^ ^30^ ^]^ Integrating in silico studies and binding assays allowed the generation and investigation of a small library of compounds sharing similar chemical functions and exhibiting a promising behavior against ROR1. To evaluate the affinity of the synthesized molecules (**10**, **13**, **14**, **17**–**21**, **26**–**29**) for the protein counterpart, SPR assay was performed against kinase domain ROR1. Protein immobilization was achieved using the amide coupling method, and the molecules were tested at 10 different concentrations, ranging from 0 to 100 μM. Ponatinib was used as a positive control^[^ ^30^ ^]^ (**Table** **1**).

**Table 1:** SPR assays of ponatinib, derivatives **10**, **13**, **14**, **17**–**21**, and **26**–**29** on ROR1 kinase domain.

Compound	Structure	Kinase domain K_D_ [μM ± SD]a)
**Ponatinib**	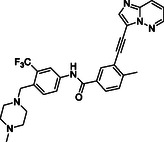	2.90 ± 0.85
**10**	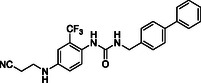	n.b.
**13**	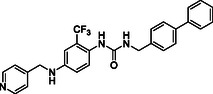	n.b.
**14**	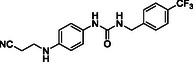	n.b.
**17**	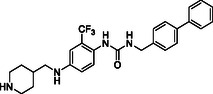	12.12 ± 2.68
**18**	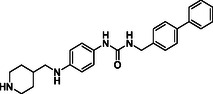	18.43 ± 3.14
**19**	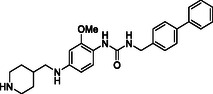	15.02 ± 2.47
**20**	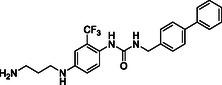	16.65 ± 2.86
**21**	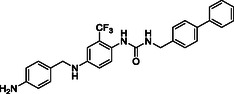	n.b.
**26**	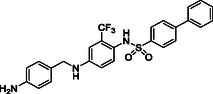	n.b.
**27**	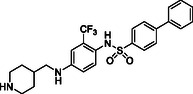	n.b.
**28**	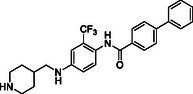	n.b.
**29**	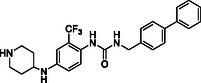	13.45 ± 1.92

a) K_D_ = dissociation constant; SD = standard deviation; n.b. = no binding.

**Figure 2:**
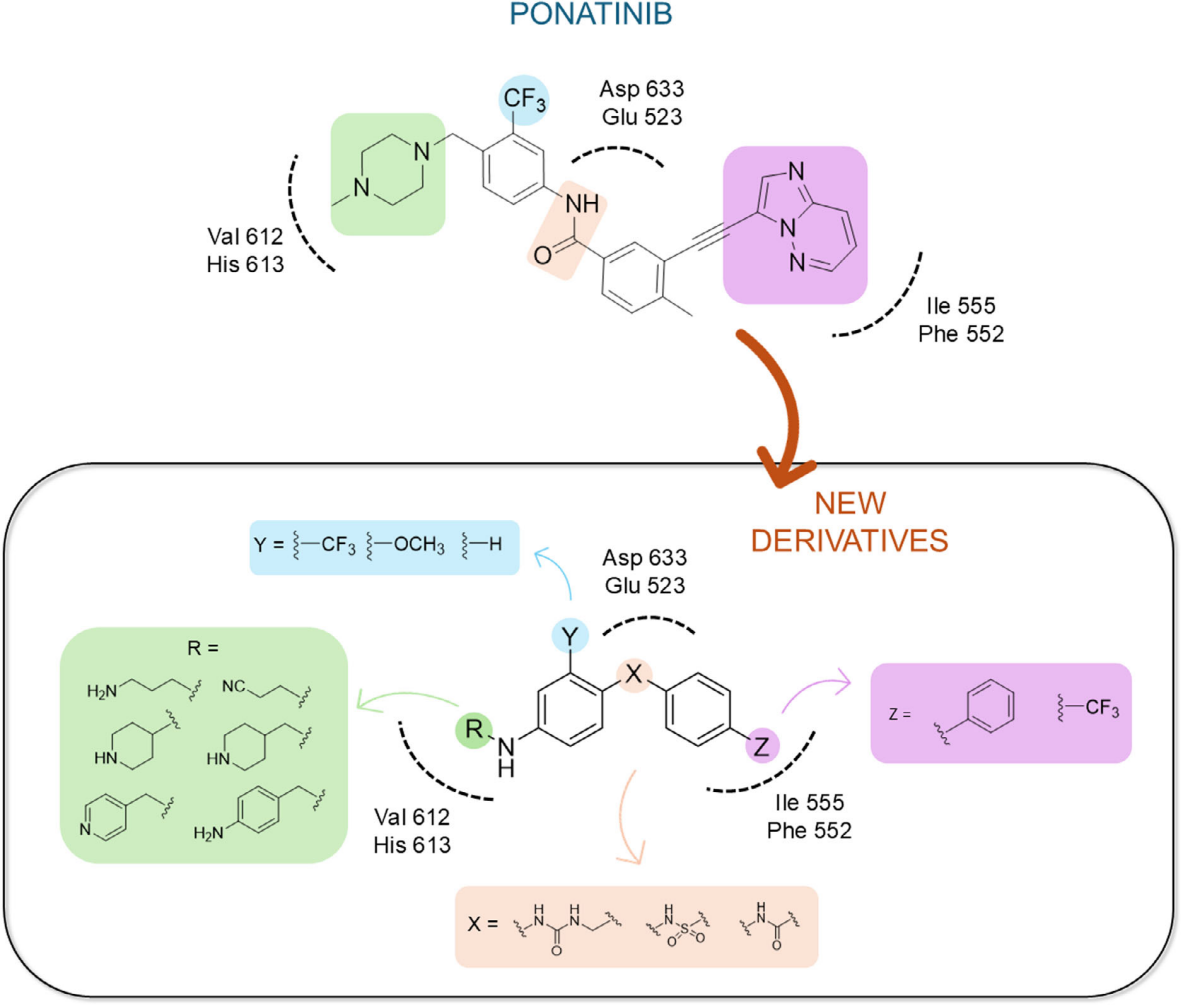
Structure‐based drug design approach leading to the *para‐*phenylenediamine derivatives acting as ROR1 inhibitors.

**Figure 3:**
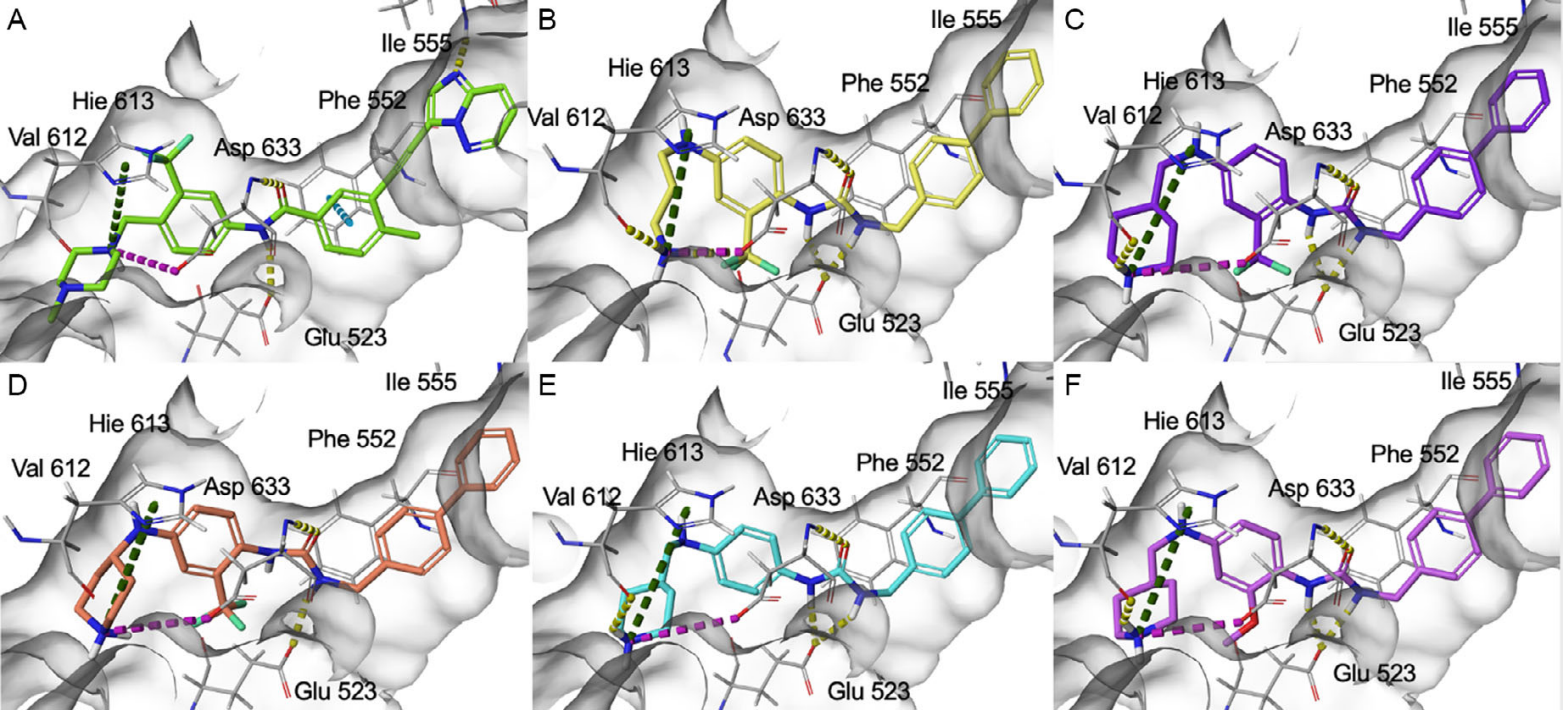
Binding mode of A) **ponatinib** (colored according to atom type: C green, O red, N blue, F light green). B) **20** (colored according to atom type: C yellow, O red, N blue, F light green, H light gray). C) **17** (colored according to atom type: C violet, O red, N blue, F light green, H light gray). D) **29** (colored according to atom type: C faded red‐orange, O red, N blue, F light green, H light gray). E) **18** (colored according to atom type: C cyan, O red, N blue, H light gray). F) **19** (colored according to atom type: C faded plum, O red, N blue, H light gray). H‐bonds, *π*‐cation interactions, and salt bridges are depicted as yellow, dark green, and purple dashed lines, respectively.

Starting from the careful analysis of the binding mode of ponatinib onto the binding site of ROR1, we identified a set of new inhibitors able to establish main interactions with the protein counterpart (see **Figure** **2**). We evaluated the use of a substituted *para*‐phenylenediamine scaffold, which we first functionalized by introducing an amide group as an exit vector for the subsequent functionalization with a biphenyl substituent (compound **28**). Compound **28** bound to the region corresponding to the ATP‐binding site of ROR1, inserting the biphenyl moiety within the binding pocket in a similar manner to the imidazo[1,2‐*b*]pyridazin moiety of ponatinib (**Figure** **3**A and Figure S1, Supporting Information). On the other hand, the amide group did not show key interactions with Asp633 and Glu523 amino acids and featured a predicted poor affinity for the protein. This was subsequently confirmed by SPR binding assays (Table 1). Then, we evaluated the replacement of the amide moiety with a sulfonamide group, obtaining compound **27** (Figure S1, Supporting Information). Unfortunately, no interesting interaction with key amino acids was disclosed, showing a different binding mode arising from the flip of the central *para*‐phenylenediamine scaffold which also led to a reduced predicted binding affinity as evincible by the docking score values (Table S1, Supporting Information). Compound **26**, in which the sulfonamide was retained and an aniline group was introduced, showed similar behavior and failed to interact with the amino acids Asp633 and Glu523 (Figure S1, Supporting Information). Biophysical data confirmed the poor binding of **26** and **27** (Table 1).

To further explore the *para*‐phenylenediamine‐based compounds, we moved to the introduction of a ureidic moiety at position 1 of the aromatic nucleus with the aim of mimicking the amide group of the reference inhibitor. The analysis of the predicted binding mode of urea‐based items highlights a better accommodation in the binding site in which the establishment of interactions with Asp633 and Glu523 was detected. Specifically, we started from compounds **21** and **13**, featuring an aniline group and a pyridine moiety, respectively which, apart from the above‐reported interactions, exhibited a loss of fundamental contacts (Figure S1 and Table S1, Supporting Information), leading to an increase of KD values. In light of these results, we evaluated the possibility of introducing a functionalized aliphatic chain with the goal of obtaining new derivatives with enhanced activity able to gain interactions in the ROR1 binding site. Compound **10**, containing a cyanoethyl group, demonstrated a poor predicted binding affinity (Figure S1 and Table S1, Supporting Information). Similarly, compound **14** did not effectively occupy the binding pocket of the receptor (Figure S1 and Table S1, Supporting Information). Considering this data, we hypothesized that the reduction of the nitrile group could re‐establish the interaction with Val612. As expected, compound **20**, featuring a propylamine chain, showed a promising binding mode, covering the entire binding site and establishing interactions with Val612 and His613 key residues of the domain (Figure 3B), as also confirmed by biophysical data. Further, the introduction of a heterocyclic group was examined, leading to derivative **17**, where the 4‐piperidin‐4‐yl‐methylamino group at position 4 of the aromatic ring exhibited two hydrogen bonds with Val612 and His613, and a salt bridge with Asp633, while urea moiety retains the same interactions pattern with Asp633 and Glu523 (Figure 3D). According to the computational data, **17** proficiently bound ROR1 with a KD = 12.12 ± 2.68 μM (Table 1). For compound **29**, in which a 4‐piperidin‐4‐yl‐amino function was introduced, we observed a similar interaction pattern to that of **17** (Figure 3D and Table 1).

Finally, the importance of the ‐CF_3_ substituent on the central aromatic ring was evaluated. Compound **18**, in which this group was removed, showed a similar affinity to that of **17** (Figure 3E and Table 1). A similar interaction pattern was observed for compound **19**, in which an ‐OCH_3_ group was introduced (Figure 3F and Table 1). In both cases, the results did not show a significant change in binding mode, suggesting that the presence or absence of electron‐withdrawing groups, such as ‐CF_3_, or electron‐donating groups, such as ‐OCH_3_, did not affect optimal ligand positioning.

### 2.3 Cytotoxicity Studies

Elevated ROR1 expression is closely linked to proliferative signaling pathways in various malignant cell types; hence, the activity reduction of this pseudo‐kinase inhibits cell proliferation and triggers apoptosis.^[^ ^23^, ^25^ ^]^ Cytotoxicity studies, assessing the activity of the synthetized compounds, were performed only on the molecules showing the best binding profile in the SPR assay. MTT test was performed on two cell lines known for overexpressing ROR1, particularly MTT assays were conducted on JeKo‐1 (human mantle cell lymphoma) and SH‐SY5Y (neuroblastoma), exposing them to different concentrations of compounds for 72 h (**Table** **2**). Derivatives **17**, **20**, and **29** exhibited significant cytotoxic activity against both cell lines, with greater potency observed in JeKo‐1. This increased effectiveness is likely due to the high expression of the target protein in leukemia and other hematologic malignancies. In contrast, **18** and **19** did not display any cytotoxic effects in either cell line. Although SPR assays confirmed their ability to bind the kinase domain of ROR1, their lack of cytotoxic activity suggests that mere binding affinity is not sufficient to confer biological efficacy, but pharmacokinetic issues cannot be excluded. A key structural difference in **18** and **19** compared to active compounds is the absence of the trifluoromethyl group on the aromatic ring. Indeed, the removal of the trifluoromethyl group (**18**) or the incorporation of an electron‐donating substituent (**19**) alters the interaction with the ATP‐binding pocket.

**Table 2:** In vitro cytotoxicity of compounds **17**–**20** and **29**. SH‐SY5Y and JeKo‐1 cells were treated for 72 h. Data are reported as EC_50_ ± SD (μM).

Compound	Structure	EC_50_ ± SD [μM]	
		SH‐SY5Y	JeKo‐1
**Ponatinib**	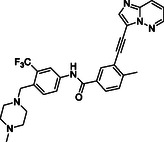	4.25 ± 3.92	1.69 ± 1.03
**17**	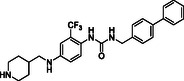	17.70 ± 2.73	10.33 ± 2.86
**18**	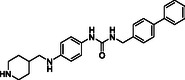	>50	>50
**19**	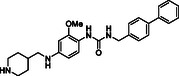	>50	>50
**20**	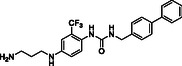	14.17 ± 4.81	11.28 ± 3.15
**29**	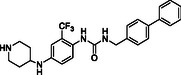	18.52 ± 2.65	13.92 ± 2.75

### 2.4 Apoptosis Evaluation

**Figure 4:**
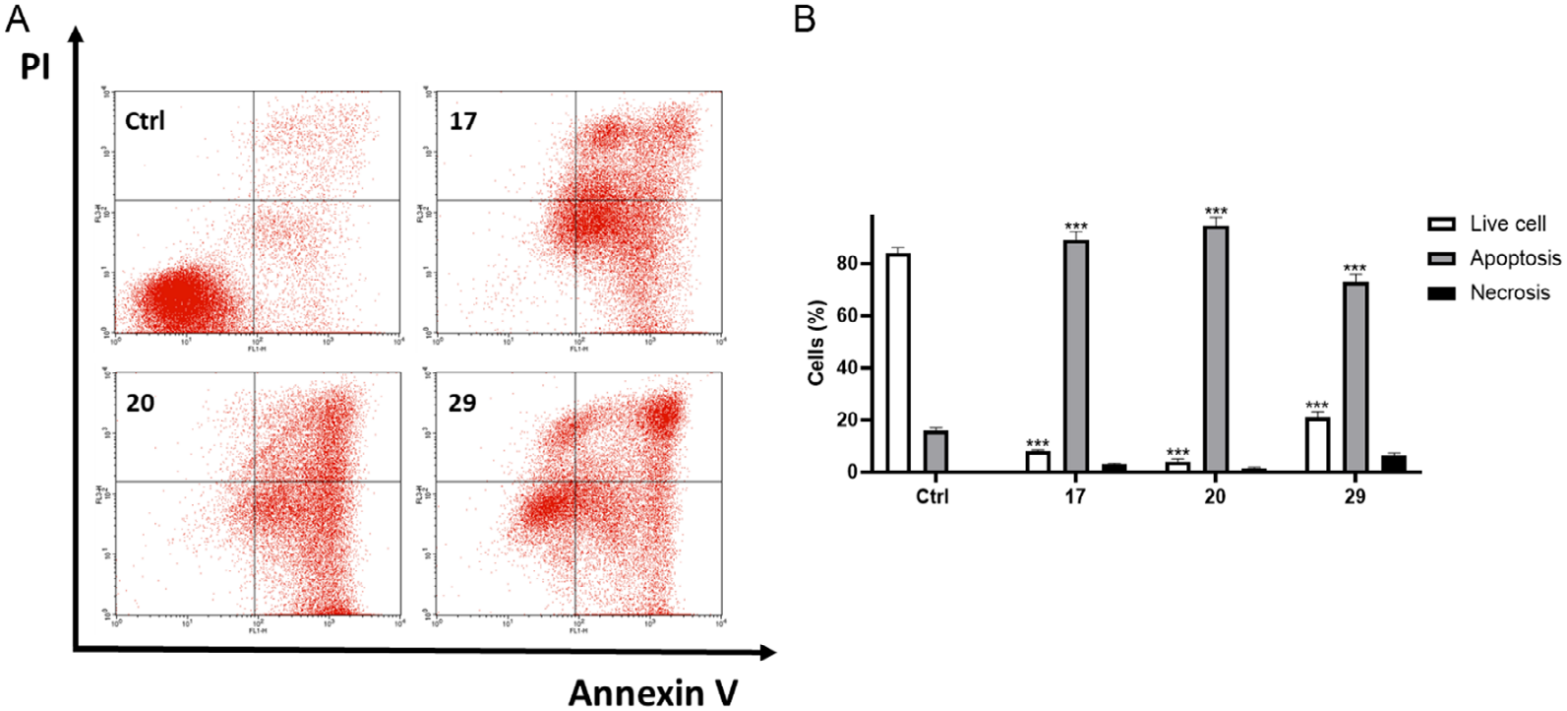
Proapoptotic activity of compounds **17**, **20**, and **29** (20 μm) on JeKo‐1 cells treated for 72 h. A) Representative flow cytometry plots using Annexin V‐FITC/PI staining for apoptosis. B) Related quantitative analysis is reported. Data are expressed as a percentage of live, apoptotic, and necrotic cells. Results are shown as mean ± standard deviation (SD) from three independent experiments. *** denote *p* < 0.001 versus Ctrl.

Initially, we tested the compounds on both SH‐SY5Y and JeKo‐1 cell lines, but preliminary viability assays indicated that the compounds exhibited significantly higher cytotoxicity in JeKo‐1 cells. This observation aligns with previous studies reporting that ROR1 is highly expressed in B‐cell malignancies, including mantle cell lymphoma, where it plays a crucial role in survival signaling and resistance to apoptosis.^[^ ^10^, ^32^ ^]^ Given the higher susceptibility of JeKo‐1 cells to the inhibitor, we selected this model for further apoptotic analysis. However, for completeness, apoptotic data for the SH‐SY5Y cell line are also provided in the Supporting Information (Figure S39, Supporting Information). Specifically, cells were incubated with compounds **17**, **20**, or **29** (20 μM) for 72 h and a flow cytometry analysis using Annexin V/PI staining was performed. After treatment, a very strong increase in the apoptotic response was observed, in both early and late apoptotic populations compared to the negative control (**Figure** **4**). Specifically, the results from FACS analysis revealed that the treatment with all the tested compounds led to an increase both in early and late apoptosis (**17**: 89.02 ± 3.12% of apoptotic cells, *p* < 0.001 versus Ctrl; **20**: 94.50 ± 3.12% of apoptotic cells, *p* < 0.001 versus Ctrl; **29**: 73.15 ± 2.76% of apoptotic cells, *p* < 0.001 versus Ctrl). These findings are in accordance with the literature and further support the potential of ROR1 inhibition in promoting cell death in mantle cell lymphoma.^[^ ^33^ ^]^

### 2.5 In‐Cell Binding Evaluation

**Figure 5:**
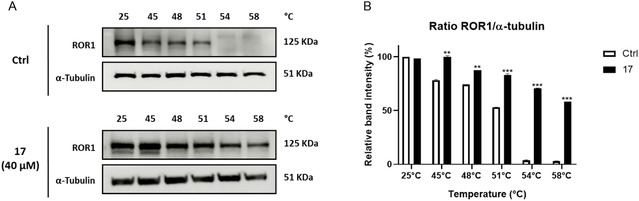
CETSA identifies ROR1 protein in the binding with **17** in JeKo‐1 cells. A) CETSA western blots and B) corresponding quantitative analysis of band intensity. **17** group was treated with **17** (40 μM) for 2 h and then subjected to 5 min incubation at the indicated temperature (45–58 °C). Ctrl group was treated with vehicle (0.1% DMSO) only in the same conditions. ROR1 level at 25 °C was set at 100%. Densitometry‐based quantification of western blotting signals was calculated by first normalizing to *α*‐tubulin levels in individual samples. Statistical analyses were performed comparing the compound **17** group to the Ctrl group across different temperature points. Results are shown as mean ± SD from three independent experiments. ** and *** denote respectively *p* < 0.01 and *p* < 0.001 versus Ctrl.

To further ascertain the binding between **17** and ROR1, we performed CETSA, a cell‐based test allowing us to carry out both qualitative and quantitative analyses of the direct interaction of a drug candidate to a target protein inside a cell.^[^ ^34^ ^]^ CETSA assay was performed on JeKo‐1 cell line incubated with **17** for 2 h. After the treatment, ROR1 levels were evaluated by western blotting analysis. Our result showed a strong stabilization of ROR1 upon treatment with **17**. In the control condition, the ROR1 signal is completely lost at 54 °C and above, suggesting protein denaturation and degradation. On the contrary, in the presence of the inhibitor, not only the ROR1 signal is significantly stronger at lower temperatures (45 °C **17**, *p* < 0.01 versus 45 °C Ctrl; 48 °C **17**, *p* < 0.01 versus 48 °C Ctrl; 51 °C **17**, *p* < 0.001 versus 51 °C Ctrl), but it also remains very detectable at 54 °C (54 °C **17**, *p* < 0.001 versus 54 °C Ctrl) and even at 58 °C (58 °C **17**, *p* < 0.001 versus 58 °C Ctrl). These results suggest that the compound binds to ROR1 with high affinity, increasing its thermal stability and confirming direct interaction. These findings provide strong evidence that the inhibitor effectively engages with ROR1 in cells, further supporting its mechanism of action (**Figure** **5**). Ponatinib was used as a positive control (Figure S40, Supporting Information).

### 2.6 Target Protein Detection

Subsequently, to understand the molecular mechanism leading to apoptosis in cancer cells upon compound **17** treatment, western blots were conducted to determine the upregulated expression of proapoptotic biomarkers and the alterations in the expression of ROR1.

**Figure 6:**
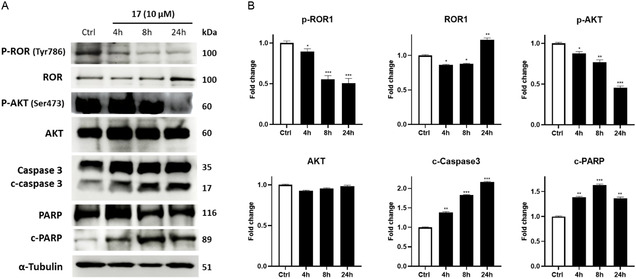
Molecular effects of **17** on ROR1 pathways in JeKo‐1 cells. For each western blotting analysis, three independent experiments were performed, and the SD is expressed by error bars. The relative fold change versus untreated cells, set as 1, is shown in the graph. *, **, and *** denote respectively *p* < 0.05, *p* < 0.01, and *p* < 0.001 versus Ctrl.

First, a marked reduction in ROR1 phosphorylation, confirming the direct inhibition of its signaling activity, was detected (**Figure** **6**A). This time‐dependent reduction in *P*‐ROR1 is particularly significant, as it represents the upstream event that drives the observed proapoptotic downstream effects. Specifically, the loss of ROR1 activation correlates with a significant decrease in AKT phosphorylation, which plays a crucial role in sustaining a prosurvival signaling in cancer cells, as we observed after the treatment with compound **17** (Figure 6B). Indeed, activation of ROR1 initiates the nonclassical Wnt (PI3K/AKT/mTOR) pathway and upregulates the phosphorylation level of AKT, which contributes to the survival and proliferation of cancer cells.^[^ ^25^ ^]^

The inhibition of this pathway creates a shift in the cellular balance toward apoptosis, as evidenced by the corresponding increased levels of cleaved caspase‐3 and PARP cleavage (Figure 6).^[^ ^23^ ^]^ These findings strongly suggest that ROR1 inhibition disrupts the survival advantage provided by AKT signaling, ultimately sensitizing cells to apoptotic processes. Moreover, the induction of apoptotic markers aligns with our previous apoptosis assay, further validating the functional impact of ROR1 inhibition.

Interestingly, despite the inhibition of ROR1 phosphorylation at 24 h, we observed an increase in total ROR1 protein levels (Figure 6). This could suggest a compensatory upregulation of ROR1 expression in response to reduced phosphorylation. It is possible that the inhibition of ROR1 activity triggers a feedback mechanism leading to increased protein synthesis or stabilization of ROR1. However, since phosphorylated ROR1 levels are reduced, this suggests that **17** effectively blocks its activation, reinforcing its role as a phosphorylation inhibitor.

Together, these results highlight the critical role of ROR1 in modulating cell survival and underscore the **17** potential to target ROR1‐expressing cells.

### 2.7 In Vitro Pharmacokinetic Evaluation

**Figure 7:**
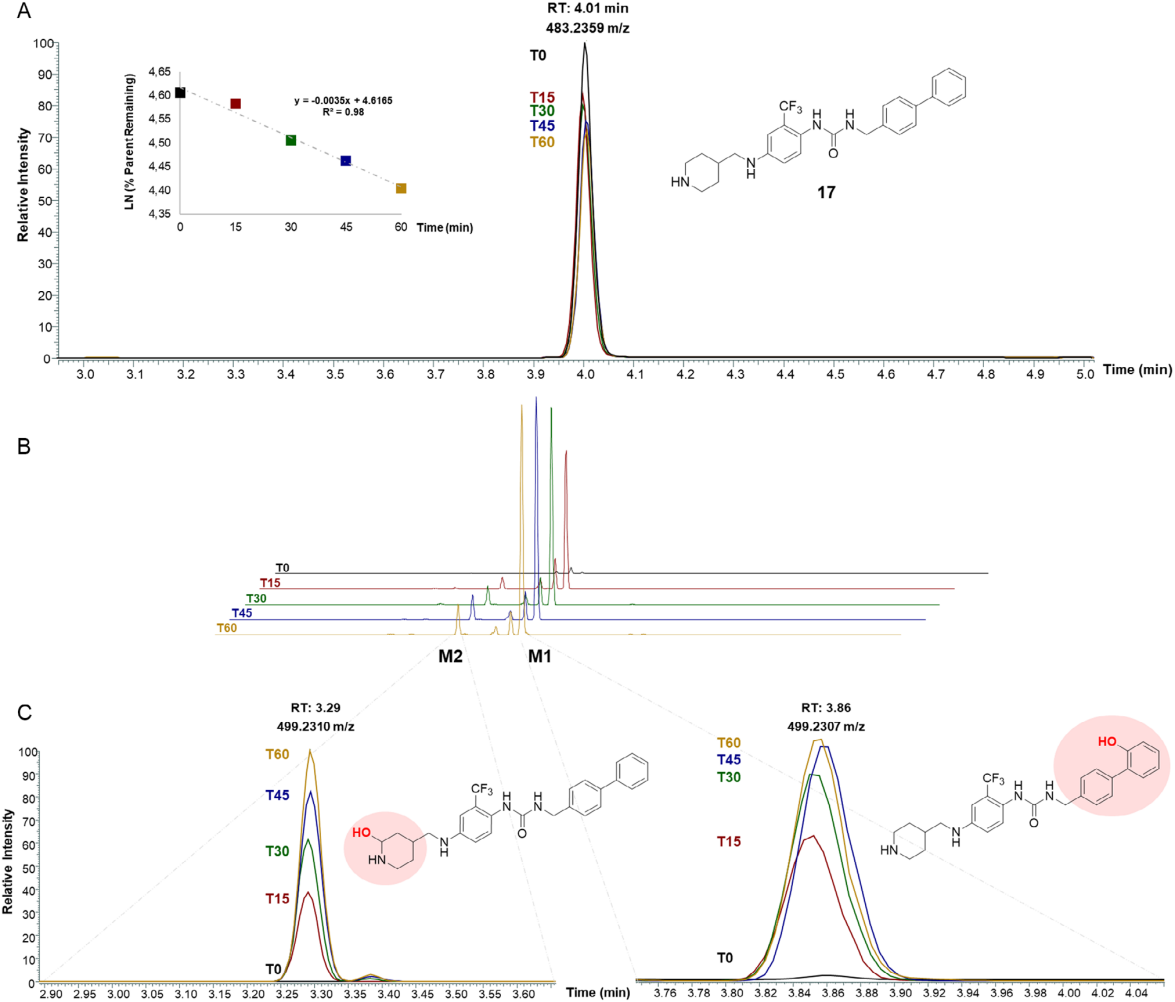
A) Compound 17 exhibited a time‐dependent decrease following incubation with mouse liver microsomes. B) LC‐MS/MS profile of mono‐oxidized metabolites of compound 17 at different time points. C) Zoomed‐in overlay of chromatograms showing M1 and M2 metabolites.

To evaluate the metabolic stability of our hit compound and assess its drug‐likeness, we investigated the microsomal stability of **17** by incubating it with mouse liver microsomes. We monitored the loss of the compound over time under CYP‐mediated metabolic pathways (**Figure** **7**A). The natural logarithm of the percentage remaining of the compound, calculated by comparing the concentration at each time point with the t0 sample, was plotted against time, yielding a linear regression (*R* ^2^ = 0.98). The slope of the linear regression (*y* = −0.0035*x* + 4.6165), representing the initial rate of metabolism, was used to determine the half‐life, which was ≈198 min. This result classifies **17** as a compound with high metabolic stability, showing ≈20% degradation after 60 min with mouse microsomes.

As the substrate underwent time‐dependent degradation, several transformation products emerged (Figure 7B). To further explore these metabolic derivatives, we putatively identified the compounds formed after microsomal incubation based on their accurate mass and fragmentation patterns. LC‐MS/MS analysis revealed that oxidation was the predominant metabolic transformation, with the main products [*m/z* 499, C_27_H_29_F_3_N_4_O_2_] showing a 16 Da mass increase compared to the parent compound [*m/z* 483, C_27_H_30_F_3_N_4_O] indicative of a single oxidation reaction.

The primary oxidative product [**M1**, *m/z* 499.2307] displayed an MS/MS spectrum characterized by a fragment ion at *m/z* 114.0912 [C_6_H_12_NO]^+^, compared to **17** at *m/z* 98.0962 [C_6_H_12_N]^+^, suggesting hydroxylation of the piperidine moiety. A minor oxidation product [**M2**, *m/z* 499.2310], detected at 3.30 min, exhibited a fragment ion at *m/z* 183.0800 [C_13_H_11_O]^+^ versus **17** at *m/z* 167.0853 [C_13_H_11_]^+^, indicating possible hydroxylation of the biphenyl group.

The proposed structures for the oxidative metabolites are putative, based on MS/MS fragmentation patterns, as no analytical standards were available for confirmation. Further experimental validation with synthesized standards is needed to confirm their identity (Figure 7C).

In addition to hepatic metabolism, drugs are also prone to degradation and modification by plasma enzymes, including hydrolases and esterases. Compounds with low plasma stability often show rapid clearance and a short half‐life. Evaluating plasma stability is a critical component of the ADME screening process, as instability can significantly impact pharmacokinetics and, ultimately, therapeutic efficacy. In this study, we assessed the metabolic stability of compound **17** by incubating it in mouse plasma for 120 min. The results demonstrated good stability, with over 85% of the compound remaining intact throughout the incubation period.

Plasma protein binding plays a crucial role in drug distribution and biological activity. After absorption into systemic circulation, drug molecules may bind to plasma proteins, and the bound drug cannot cross biological membranes, rendering it unavailable to exert a pharmacological effect. Only the free drug, unbound to plasma proteins, is pharmacologically active. Further, the plasma protein binding affinity of compound **17** was assessed, revealing a high binding affinity, with 99% of the drug bound to plasma proteins.

## 3 Conclusion

Despite the recent progress in cancer treatment, it still represents an open challenge for the scientific community. In this field, the identification of ROR1 as a molecular target of several tumor types defines an opportunity for finding novel anticancer drugs. Considering that the main plague of the current chemotherapy is based on its side effects, a target selectively expressed in tumoral conditions could be very profitable to reduce the complications of the anticancer treatment. Moreover, targeting ROR1 offers the advantage of decreasing the pharmaco‐resistance and improving the chemotherapy response, making this approach valuable in coadministration protocols. Unfortunately, the ROR1 cellular pathway is not fully understood and very few attempts have been made to develop ROR1 inhibitors. Hence, we report a preliminary exploration of the structure–activity relationship of a series of *para*‐phenylenediamine‐based compounds developed as ROR1 inhibitors. Pilot in silico studies combined with a structure‐based drug design approach drove the definition of the molecular space suitable for the building of novel derivatives acting against ROR1 protein; the binding analyses performed step‐by‐step revealed that 5 of the 12 synthesized compounds exhibited good affinity to the pseudo‐kinase and were evaluated for their cytotoxic effect in two different cell lines overexpressing ROR1. The encouraging results obtained from JeKo‐1 cells led us to analyze the proapoptotic mechanism of the most effective synthesized derivatives, confirming that ROR1 inhibition promoted cell death in mantle cell lymphoma. Based on the previous experiments, 17 showed the best pharmacological profile and it was selected for further *in cell* binding analyses. CETSA test corroborated the binding affinity of 17, shedding further light on the hypothesized mechanism of action. Again, an in vitro pharmacokinetic study allowed us to assess the drug‐likeness profile of the most active compound.

Overall, this study represents a preliminary definition of novel chemical entities able to inhibit ROR1 kinase, validating the *para*‐phenylenediamine scaffold as suitable core for the synthesis of antitumor compounds and paving the way for future structural modifications that can lead to the development of more effective ROR1 modulators.

## 4 Experimental Section

4.1

4.1.1

##### In Silico Studies

3D structure of ROR1 pseudo‐kinase domain bound to ponatinib (PDB ID: 6TU9)^[^ ^30^ ^]^ was downloaded and processed using Protein Preparation Wizard software (Schrödinger Suite),^[^ ^35^, ^36^ ^]^ cap termini were incorporated, hydrogen atoms were added, and bond orders were assigned after the removal of all water molecules, solvent, and cocomplexed compound. The grid for the subsequent molecular docking experiment was generated using the cocrystallized ligand as reference. The final center coordinates were − 18.43 (*x*), 13.75 (*y*), and −5.15 (*z*). The inner dimensions of the box were 10 Å while the outer dimensions were 31.84 Å.

Compounds investigated were drawn using 2D Sketcher (Maestro, Schrödinger Suite) and prepared using LigPrep software (Schrödinger Suite).^[^ ^37^ ^]^ All the possible tautomers and protonation states were generated at pH = 7.4 ± 1.0. After that, the obtained structures were minimized using the OPLS 2025 force field.

Molecular docking experiments were performed using Glide software and setting the Extra Precision (*XP*) mode.^[^ ^36^, ^37^, ^38^, ^39^, ^40^, ^41^, ^42^ ^]^ From the 10,000 poses generated in the initial docking phase, 800 conformations were retained and subjected to the minimization step with an energy threshold of 0.15 kcal mol^−1^. Finally, a maximum of 20 poses were saved for each compound.

##### General

All the reagents for the synthesis were purchased from Merck (Milan, Italy) unless otherwise described; all solvents and additives were purchased by Merck (Darmstadt, Germany) and were of reagent grade for synthetic purposes and LC‐MS grade for analytical purposes. Reactions were carried out with magnetic stirring in round‐bottom flasks except for microwave‐assisted reactions that were conducted using glass vials and microwave closed vessel apparatus (CEM, Discover 2.0, Charlotte, North Carolina). Moisture‐sensitive reactions were conducted in oven‐dried glassware under nitrogen stream, using freshly distilled solvents. TLC analysis of reaction mixtures was performed on precoated glass silica gel plates (F254, 0.25 mm, VWR International), while crude products were purified by the Isolera Spektra One automated flash chromatography system (Biotage, Uppsala, Sweden), using commercial silica gel cartridges (SNAP KP‐Sil, Biotage). NMR spectra were recorded on a Bruker Avance 400 MHz apparatus, at room temperature. Chemical shifts were reported in *δ* values (ppm) relative to internal Me_4_Si for ^1^H and ^13^C‐DEPTq. *J* values were reported in hertz (Hz). ^1^H NMR peaks were described using the following abbreviations: *s* (singlet), *bs* (broad singlet, *d* (doublet), *t* (triplet), and m (multiplet). HR‐MS spectra were recorded by LTQ‐Orbitrap‐XL‐ETD mass spectrometer (Thermo Scientific, Bremen, Germany), equipped with an ESI source. The purity of final compounds was assessed by ultrahigh‐performance liquid‐chromatography (UHPLC) analyses, performed on a Jasco Extrema LC 4,000 (Jasco, Japan) consisting of an LC‐Net CG cable controller, quaternary flow pump system PU‐4,285, a DG‐4,000–04 thinsp;degasser, a UV ‐ 4,075 detector, and an AS‐4,250 autosampler. Purity assessment UHPLC runs were carried out on an EVO C18 150  × 2.1 mm × 2.6 μm (100 Å) column (Phenomenex, Bologna, Italy) and chromatograms were monitored at 254 nm. The optimal mobile phase consisted of 0.1% HCOOH/H_2_O v/v (A) and 0.1% HCOOH/ACN v/v (B). Analysis was performed in gradient elution as follows: 0–10.00 min, 5–95% B; 10–12.00 min, 95–95% B; 12–15.00 min, isocratic to 5% B. Flow rate was 0.5 mL min^−1^. The injection volume was set at 5 μL.

##### General Procedure A: Urea Synthesis (1‐4)

To a solution of the proper aniline (4.0 mmol) in acetonitrile (25 mL), potassium *tert*‐butoxide (6.0 mmol) and 4‐(isocyanatomethyl)‐1,1′‐biphenyl or 4(trifluoromethyl)phenyl isocyanate (6.0 mmol) were added and the mixture was stirred at 100 °C for 12 h. Afterward, the reaction mixture was diluted with dichloromethane (20 mL), and the resulting solution was washed with a 2M aqueous solution of HCl (2 × 25 mL), dried over Na_2_SO_4_, filtered, and concentrated in vacuo. Flash chromatography of the residues, using mixtures of n‐hexane/ethyl acetate as mobile phase furnished the corresponding ureidic derivatives in 39–68% of yield.

##### General Procedure B: Hydrogenation (5–8, 21, 24, 25)

To a solution of starting material (3.0 mmol) in a mixture of THF/MeOH (1/:1 v/:v, 20 mL), ammonium formate (30 mmol) and Pd/C (10% mol) were added. The reaction mixture was refluxed at 100 °C until the complete disappearance of the starting material, monitored by TLC. After completion, the reaction solution was filtered through Celite 503 (Merck Millipore, Burlington, USA) and evaporated under vacuum. The compounds were purified by flash chromatography using mixtures of n‐hexane/ethyl acetate as eluent system (yield 75–92%).

##### General Procedure C: Reductive Amination (9, 12, 13, 15, 16)

The proper amino intermediate (1.29 mmol) was dissolved in chloroform (15 mL), added with 1‐Boc‐piperidine‐4‐carboxaldehyde, 4‐nitrobenzaldehyde or 4‐pyridinecarboxaldehyde (1.55 mmol) and molecular sieves, and refluxed overnight under nitrogen atmosphere. Then, NaBH_4_ (3.9 mmol) was added and the mixture was reacted for further 1 h at room temperature. Afterward, the reaction was diluted with ethyl acetate, washed with water (2 × 20 mL), and extracted. The organic layer was dried over Na_2_SO_4_, filtered, and evaporated. The crude products were purified by flash chromatography using mixtures of DCM/ethyl acetate as eluent affording the corresponding derivatives in 40%‐57% of yield.

##### General Procedure D: Boc Removal (17–20, 27–29)

The amino‐Boc intermediates (0.8 mmol) were dissolved in a mixture of TFA/DCM (1/3, v/v, 10 mL), and triisopropylsilane (TIS, 0.25 mmol) was added. Reactions were stirred at room temperature for 3 h until completion. Then, the mixtures were diluted with DCM (10 mL) and washed with a solution of NaOH (2 N, 10 mL). Upon extraction, the crudes were dried over Na_2_SO_4_, filtered, and concentrated in vacuo. The final products were precipitated from mixtures of MeOH/diethyl ether and used in the following reaction without further purification step.

##### General Procedure E: Alkylation Reaction (10, 11, 14)

A mixture of intermediate 5 or 6 (1.3 mmol), the proper alkyl halide (2.6 mmol), and potassium *tert*‐butoxide (2.6 mmol) in acetonitrile (6 mL) was heated to 120 °C (150 Watt) in a 10 mL microwave reactor for 1 h. Afterward, the reaction mixture was diluted with dichloromethane (20 mL), and the resulting solution was washed with a 10% aqueous solution of NaHCO_3_ (2 × 25 mL), dried over Na_2_SO_4_, filtered, and concentrated in vacuo. Flash chromatography of the residues, using mixtures of n‐hexane/ethyl acetate or dichloromethane/ethyl acetate as eluent system, furnished the corresponding alkylated compounds in 35–48% of yield.

##### SPR Assay

Recombinant human ROR1 protein (Fc Tag) and recombinant human ROR1 protein (aa 453‐783, His&GST Tag) were purchased from Elabscience (Texas, USA). SPR spectroscopy analyses were conducted to evaluate the binding affinity of the synthesized compounds against ROR1 receptor using a Biacore T200 optical biosensor equipped with research‐grade CM5 sensor chips (Cytiva, Marlborough, USA). ROR1 kinase domain was immobilized on the CM5 sensor chip surface following standard amine‐coupling protocols. Specifically, kinasic domain was immobilized at pH 4.5 10 mM CH_3_COONa using a flow rate of 10 μL min^−1^, achieving a density of around 20 kRU.

For the experiments, surfaces of ROR1, along with an unmodified reference surface, were prepared for simultaneous analyses. Compounds were dissolved to prepare 10 mM solutions in 100% DMSO, then diluted 1:20 *v*/*v* in PBS‐P (PBS–P buffer: 0.2 M phosphate buffer, 27 mM KCl, 1.37 M NaCl, 0.5% surfactant P20) to achieve a final DMSO concentration of 5.0%. The compounds were injected in a series of concentrations (1:2 dilution, 10 different concentrations), spanning from 0 to 100 μM. The concentration series were prepared in 96‐well plates. SPR experiments were conducted at 25 °C, with a flow rate of 20 μL min^−1^, allowing for 90 s of association monitoring and 400 s of dissociation monitoring. Changes in mass, reflective of the binding response, were recorded as resonance units (RU). *K*_D_ values were determined using the Biaevaluation software performing a global fit of the double‐referenced association and dissociation data to a 1:1 interaction model^[^ ^43^ ^]^ (see Figure S38, Supporting Information).

##### Cell Culture

The neuroblastoma SH‐SY5Y cells, a human neuroblastoma cell line of neural crest origin, were obtained from ATCC and were grown in DMEM supplemented with 10% *v/v* fetal bovine serum, 2 mM L‐glutamine, 100 U mL^−1^ penicillin, and 0.1 mg mL^−1^ streptomycin.

The JeKo‐1 mantle cell lymphoma (MCL) cell line, derived from mantle cell lymphoma of B‐cell lineage, was obtained from ATCC and was grown in RPMI supplemented with 10% *v/v* fetal bovine serum, 2 mM L‐glutamine, 100 U mL^−1^ penicillin, and 0.1 mg mL^−1^ streptomycin.

Cells were routinely grown in culture dishes (Corning, Corning, New York) in an environment containing 5% CO_2_ at 37 °C and passaged at confluence using a solution of 0.025% trypsin and 0.01% EDTA. Cell growth and viability were monitored using phase‐contrast microscopy and trypan blue staining.^[^ ^44^ ^]^ In each experiment, cells were placed in a fresh medium, cultured in the presence of synthesized compounds, and followed for further analyses. All experiments were performed in triplicate.

##### Cell Viability Assay

Cell viability was established by measuring mitochondrial metabolic activity with MTT. In brief, SH‐SY5Y (20 × 10^3^ cells/well) and JeKo‐1 (10 × 10^3^ cells/well) were plated into 96‐well plates for 24 h, then the compounds (1.56–50 μM) were added. After 24 h, cells were replaced with a fresh medium containing 0.5 mg mL^−1^ MTT.^[^ ^45^ ^]^ Cells were incubated for 1–3 h at 37 °C, then the medium was discarded, and formazan blue crystals in the cells dissolved with 100 μL per well of a solution containing isopropanol/HCl 0.1 M. The absorbance was measured at 570 nm using a microplate reader (Multiskan Go, Thermo Scientific, Waltham, MA, USA). Cell viability was expressed as a percentage relative to the untreated cells cultured in medium with 0.1% DMSO and set to 100%, whereas 10% DMSO was used as positive control and set to 0% of viability. The EC_50_ values were calculated using GraphPad Prism 8.0 software by nonlinear regression of dose‐response inhibition.

##### Annexin V‐FITC/PI Staining

Apoptosis of the cells was assessed using annexin V‐FITC/PI reagents. JeKo‐1 cells (60 × 10^3^ cells/well) were seeded into 24‐well plates and incubated for 72 h with compounds (20 and 10 μM). After treatment, the collected cells were resuspended in a 100 μL assay buffer, then 5 Annexin V‐FITC and 1 PI reagents were added following incubation for 20 min at room temperature according to the manufacturer's protocol (Dead Cell Apoptosis Kits with Annexin V for Flow Cytometry, Thermo Fisher Scientific). Cells were analyzed with a Becton Dickinson FACScan flow cytometer using the Cell Quest software, version 4 (Franklin Lakes, NJ, USA).^[^ ^46^ ^]^

##### Western Blotting Analysis

JeKo‐1 cells (2.5 × 10^6^ cells/well) were seeded in a T‐75 flask, treated with compound 17 (10 μM) for different times (4, 8, and 24 h). After treatments, cells were washed twice with PBS and detached with a scraper, centrifuged at 655* g* × 10 min at 4 °C. Full proteins were extracted by lysis buffer (20 mM Tris‐HCl pH 7.5, 150 mM NaCl, 1 mM Na_2_EDTA, 1 mM EGTA, 2% NP‐40, 1% sodium deoxycholate, 1× protease and phosphatase inhibitor cocktail) for 30 min. Then, cell lysates were centrifuged at 4850* g* for 20 min at 4 °C. 60 μg of total proteins were run on 8% SDS‐PAGE and transferred to nitrocellulose membranes using a minigel apparatus (Bio‐Rad Laboratories, Hercules, CA, USA). Blots were blocked in phosphate‐buffered saline, containing Tween‐20 0.1% and 5% BSA for 1 h at room temperature and incubated overnight with specific primary antibodies at 4 °C with slight agitation. *α*‐Tubulin was used as the loading control. The following antibodies were used: rabbit polyclonal anti‐ROR1 (Thermo Fisher Scientific, Waltham, MA, USA), rabbit polyclonal anti‐phospho‐ROR1 (Tyr786) (Thermo Fisher Scientific, Waltham, MA, USA), rabbit monoclonal anti‐AKT (Santa Cruz Biotechnology, Dallas, TX, USA), rabbit monoclonal anti‐phospho‐AKT (Santa Cruz Biotechnology, Dallas, TX, USA), rabbit monoclonal anti‐PARP (Cell Signaling Technology, Danvers, MA, USA), and mouse monoclonal anti‐α‐tubulin (Santa Cruz Biotechnology, Dallas, TX, USA). After washing in PBS/Tween‐20 0.1%, the appropriate antirabbit or antimouse (Pierce, Thermo Fisher Scientific, Waltham, MA, USA) peroxidase‐linked secondary antibody was added for 1 h at room temperature. Antigen‐antibody complexes were detected by enhanced chemiluminescence (ECL kit, Amersham, Germany). Filters were exposed to LAS 4000 (GE Healthcare, Chicago, IL, USA) and the densitometry analysis of autoradiographs was performed by the ImageJ program, version 1.47.

##### Cellular Thermal Shift Assay

The CETSA is particularly suitable for identifying the interaction between ligands and their protein targets. This method is based on the principle of thermodynamic stabilization of the protein due to ligand binding. The change in the thermal stability of the target protein is measured by the amount of residual soluble protein at different temperatures, comparing treated samples with control samples.^[^ ^32^ ^]^ JeKo‐1 cells (5 × 10^6^) were incubated with compound 17 (40 μM) for 2 h. Ponatinib (10 μM) was used as a positive control. After treatment, the cells were harvested and centrifuged at 655* g* for 5 min, and the cell pellet was resuspended in PBS. The samples were then divided into 6 aliquots, each subjected to a 5 min incubation at a specific temperature ranging from 25 to 58 °C. Afterward, the samples were lysed and centrifuged to separate soluble proteins from aggregated and precipitated proteins. The amount of the soluble target protein was assessed by western blotting analysis.

##### Statistical Analysis

Data are reported as mean ± SD of results from three independent experiments. Comparisons between the groups were analyzed by one‐way analysis of variance (ANOVA), followed by Bonferroni's test, with GraphPad Prism 8.0 software (San Diego, CA, USA). Significance was assumed at *p* < 0.05.

##### Microsomal Stability

The liver microsomal stability assay of compound 17 was conducted as previously described.^[^ ^47^ ^]^ Briefly, mouse (CD‐1) liver microsomes (Thermo Fisher Scientific, Bremen, Germany) were used, and the reaction was initiated by the addition of NADPH. The incubation was carried out 37 °C for 15, 30, 45, and 60 min in a Thermomixer comfort (Eppendorf, Hamburg, Germany). The reaction was stopped by the addition of ice‐cold methanol containing IS. Finally, the samples were centrifuged, and the supernatants were injected into the LC‐MS. The control at 0 min was established by adding the organic solvent immediately after incubation with microsomes. Testosterone was employed as positive control, while the negative control involved incubation without cofactor for up to 60 min. All experiments were performed in triplicate, and results were expressed as the in vitro microsomal half‐life (*t*_1/2_), calculated using the equation *t*_1/2 _= 0.693/*b*, where *b* is the slope of the linear regression obtained by plotting the natural logarithm of the remaining parent compound fraction against incubation time.

##### Plasma Stability Assay

The plasma stability of compound 17 was evaluated. Briefly, mouse plasma is equilibrated to 37 °C and biotransformation is initiated by addition of compound solution and mixing. At each specified time point (0, 60, and 120 min), the test compound was extracted using ice‐cold methanol to halt degradation, with the IS added during the quenching phase. The concentration of the test compound was then quantified using LC‐MS. The percentage of the test compound remaining at each time point, relative to the 0 min reference, was recorded. Procaine (low stability) and procainamide (high stability) served as controls. All experiments were conducted in triplicate.

##### Plasma Protein Binding

A Rapid Equilibrium Dialysis Plate (Pierce™ RED, Thermo Scientific) was used to determine the percentage of compound binding to plasma proteins. Potassium phosphate buffer was added to each white well, and plasma/compound (1 μM) mixture was added to the red wells. The plate was sealed and incubated at 37 °C on an orbital shaker at 300 rpm for 4 h. Afterward, 50 μL of post‐dialysis samples from the buffer and plasma chambers were transferred into separate microcentrifuge tubes. An equal volume of plasma was added to the buffer sample, while the plasma sample received an equivalent volume of buffer. Precooled IS was added to the assay plate to quench the reaction. Compounds were quantified by LC‐MS/MS. The percentage of free and bound compound was calculated using the following equations: % free = (concentration in buffer chamber/concentration in plasma chamber) × 100, and % bound = 100 − % free.^[^ ^48^ ^]^ All experiments were performed in duplicate. Propranolol was used as positive control.

##### Instrumentation

LC‐MS/MS analysis was performed on a Vanquis UHPLC system connected online to an Orbitrap Exploris™ 120 mass spectrometer (Thermo Fisher Scientific, Bremen, Germany) equipped with a heated electrospray ionization probe (HESI II).

##### LC‐MS/MS Conditions

The chromatographic separation was performed on a Kinetex 2.6 μm Evo C18 column (100 × 2.1 mm, Phenomenex) using H_2_O (A) and ACN (B), both acidified with 0.1% formic acid, with the following gradient: 0.01–10.00 min, 5–95% B; isocratic at 95% B for 1 min; 11.01–11.50 min, 95–5% B; followed by 1.5 min for re‐equilibration.

The flow rate and column temperature were set at 0.4 mL min^−1^ and 40 °C, respectively.

The ESI operated in positive mode. Full MS parameters: Orbitrap resolution 60,000; scan range (*m/z*) 100–1500; RF lens 70%; AGC target 200%; maximum injection time 200 ms.

Data‐dependent MS/MS: Orbitrap resolution 15,000; isolation window 2 *m/z*; normalized HCD collision energy 30%. Ion source parameters: sheath gas 60; auxiliary gas 15; sweep gas 2; ion transfer tube and vaporizer temperature 300 °C; spray voltage + 3.4 kV/−3.0 kV.

##### Calibration Curve

For the calibration curve, the primary stock solution was prepared in DMSO. Intermediate and working standard solutions were obtained by serial dilution in methanol. Tolbutamide (1 μM) was used as the internal standard (IS). The calibration curve covered a concentration range of 0.125–20 μM with seven levels, each analyzed in triplicate. Linear regression of the response ratios (peak area analyte/peak area IS) generated the calibration curve (*y* = 1.9516*x* − 0.0373) with a correlation coefficient (*R* ^2^) of ≥ 0.9996.

## Conflict of Interest

The authors declare no conflict of interest.

## Supporting information

Supplementary Material

## Data Availability

The data that support the findings of this study are available on request from the corresponding author. The data are not publicly available due to privacy or ethical restrictions.
