# Supplementary material for: Novel Para‐Phenylenediamine‐Based Derivatives as Receptor Tyrosine Kinase‐like Orphan Receptor 1 (ROR1) Inhibitors: An In Vitro Preliminary Characterization
Source: ChemMedChem. 2025 Jun 1;20(14):e202500247. doi: 10.1002/cmdc.202500247 (PMC12276035; doi:10.1002/cmdc.202500247)
Supplement: Supplementary file 1 — Supplementary Material [file CMDC-20-e202500247-s001.pdf]

SUPPLEMENTARY INFORMATION

Novel *para*-phenylenediamine-based derivatives as Receptor Tyrosine Kinase-like Orphan Receptor 1 (ROR1) Inhibitors: An in vitro preliminary characterization

Gerardina Smaldone, ǁ,[a] Maria Rosaria Miranda, ǁ,[a] Francesca Di Matteo, [a] Valeria Napolitano, [a] Michela Aliberti, [a] Simona Musella, [a] Veronica Di Sarno, [a] Gianluigi Lauro, [a] Giuseppe Bifulco, [a] Giacomo Pepe, [a] Giovanna Aquino, [a] Mario Felice Tecce, [a] Isabel M. Gomez-Monterrey, [b] Pietro Campiglia, [a] Carmine Ostacolo, [a] Alessia Bertamino, [a] Vincenzo Vestuto, *,[a] and Tania Ciaglia. *,[a]

[a] Dr. G. Smaldone, Dr. M.R. Miranda, Dr. F. Di Matteo, Dr. V. Napolitano, Dr. M. Aliberti, Prof. S. Musella, Prof. V. Di Sarno, Prof. G. Lauro, Prof. G. Bifulco, Prof. G. Pepe, Dr. G. Aquino, Prof. M.F. Tecce, Prof. P. Campiglia, Prof. C. Ostacolo, Prof. A. Bertamino, Dr. V. Vestuto, Dr. T. Ciaglia
Department of Pharmacy
University of Salerno
Via G. Paolo II 132, 84084, Fisciano, Salerno, Italy
E-mail: vvestuto@unisa.it; tciaglia@unisa.it

[b] Prof. I. M. Gomez-Monterrey
Department of Pharmacy
University Federico II of Naples
Via D. Montesano 49, 80131, Naples, Italy

ǁ These authors contributed equally to the work

Table of content:

Table S1: Docking score and interactions reported by ponatinib and compounds **10**, **13**, **14**, **17-21**, **26-29** evaluated against ROR1……………………………………………**S2**

Figure S1: Binding mode of compounds **10**, **13**, **14**, **21**, **26-28**……………………………**S3**

Figures S2-S37: NMR spectra and HPLC traces of synthesized compounds ……………**S4-S27**

Figures S38: SPR assay raw data……………………………………………**S28-S29**

Figure S39: Representative flow cytometry plots…………………………………**S30**

Figures S40-41: CETSA western blots raw data……………………………………**S31-S33**

Experimental Section: Chemistry……………………………………………**S34-S37**

Table S1. Docking score and interactions reported by ponatinib and compounds **10**, **13**, **14**, **17-2**1, **26-29** evaluated against ROR1.

Compound	Docking score (Kcal/mol)	Interactions
**Ponatinib**	-14.810	H bonds: Glu523, Asp633, Ile555; p-p stacking: Phe552; Salt bridge: Asp633; p-cation: His613
**17**	-11.134	H bonds: Glu523, Asp633, Val612, His613; Salt bridge: Asp633; p-cation: His613
**28**	-9.419	H bonds: Asp633; p-cation: His613; p-p stacking: Phe552
**27**	-5.595	H bond: Glu523
**26**	-7.105	H bond: Asp633; p-p stacking: Phe552
**29**	-10.910	H bonds: Glu523, Asp633; Salt bridge: Asp633 p-cation: His613
**10**	-10.020	H bonds: Glu523, Asp633
**14**	-8.375	H bond: Glu523
**20**	-12.308	H bonds: Glu523, Asp633, Val612; Salt bridge: Asp633; p-cation: His613
**21**	-7.239	H bond:Asp633
**13**	-10.919	H bonds: Glu523, Asp633
**18**	-10.301	H bonds: Glu523, Asp633, Val612; His613; Salt bridge: Asp633; p-cation: His613
**19**	-10.882	H bonds: Glu523, Asp633, Val612; His613; Salt bridge: Asp633; p-cation: His613

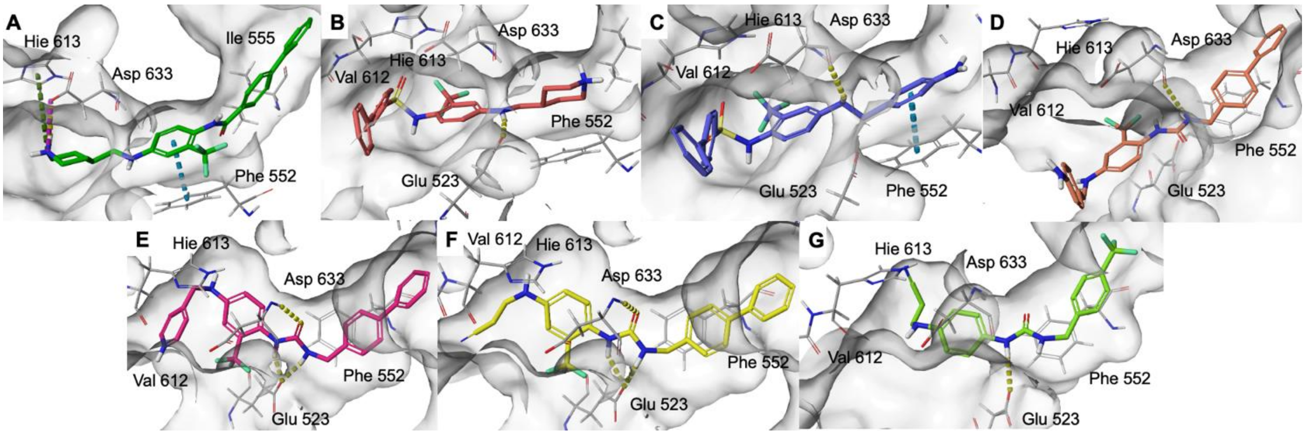

Figure S1. Binding mode of A) **28** (coloured according to atom type: C green, O red, N blue, F light green), B) **27**(coloured according to atom type: C faded red, O red, N blue, F light green, H light grey), C) **26** (coloured according to atom type: C faded blue, O red, N blue, F light green, H light grey), D) **21** (coloured according to atom type: C faded red-orange, O red, N blue, F light green, H light grey), E) **13** (coloured according to atom type: C cyan, O red, N blue, H light grey), F) **10** (coloured according to atom type: C yellow, O red, N blue, H light grey) G) **14** (coloured according to atom type: C faded green, O red, N blue, H light grey). H-bonds, p-p interactions, p-cation interactions and salt bridges are depicted as yellow, blue, dark green and purple dashed lines, respectively.

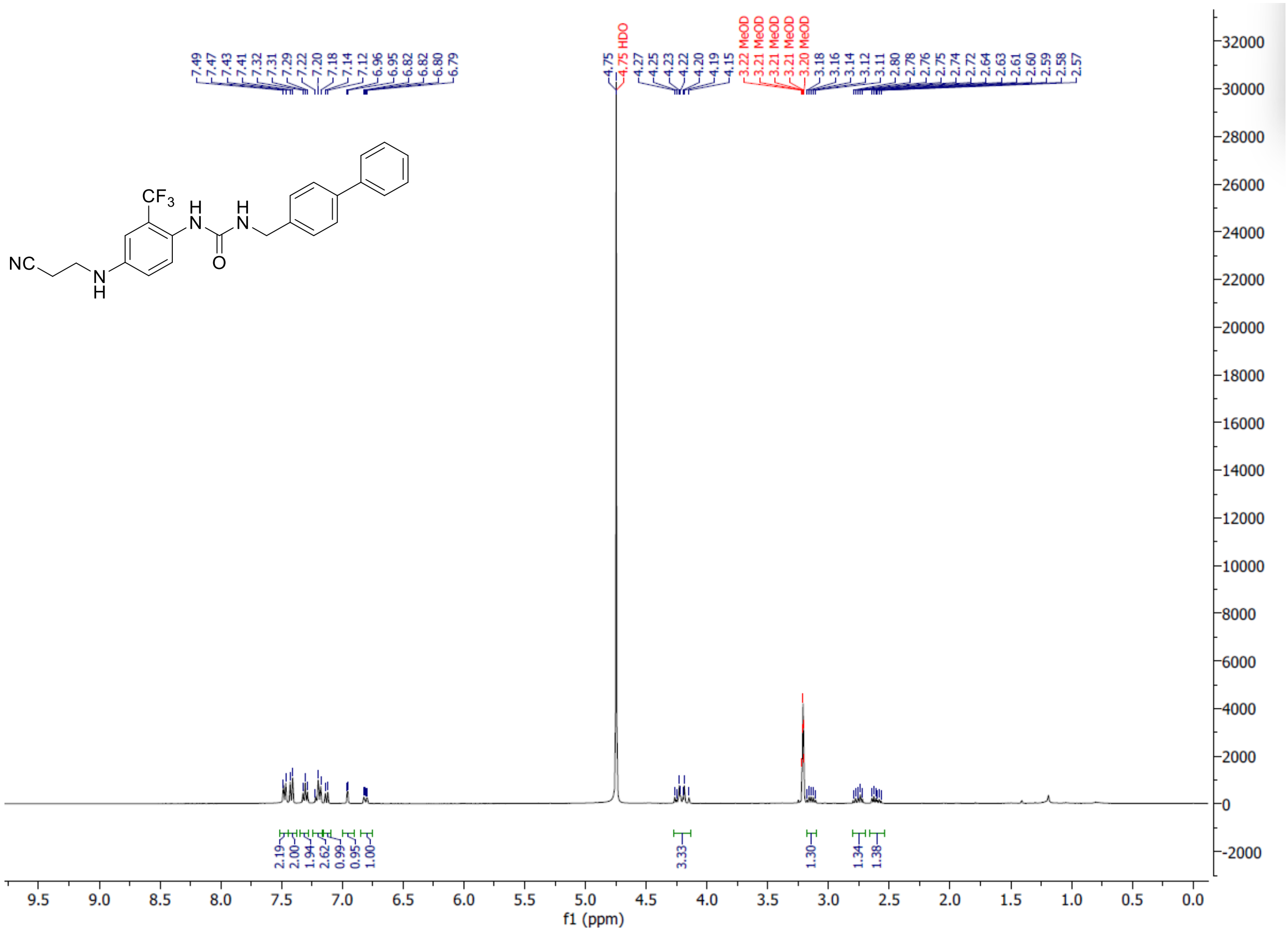

Figure S2: 1H NMR spectrum of compound **10** (MeOD).

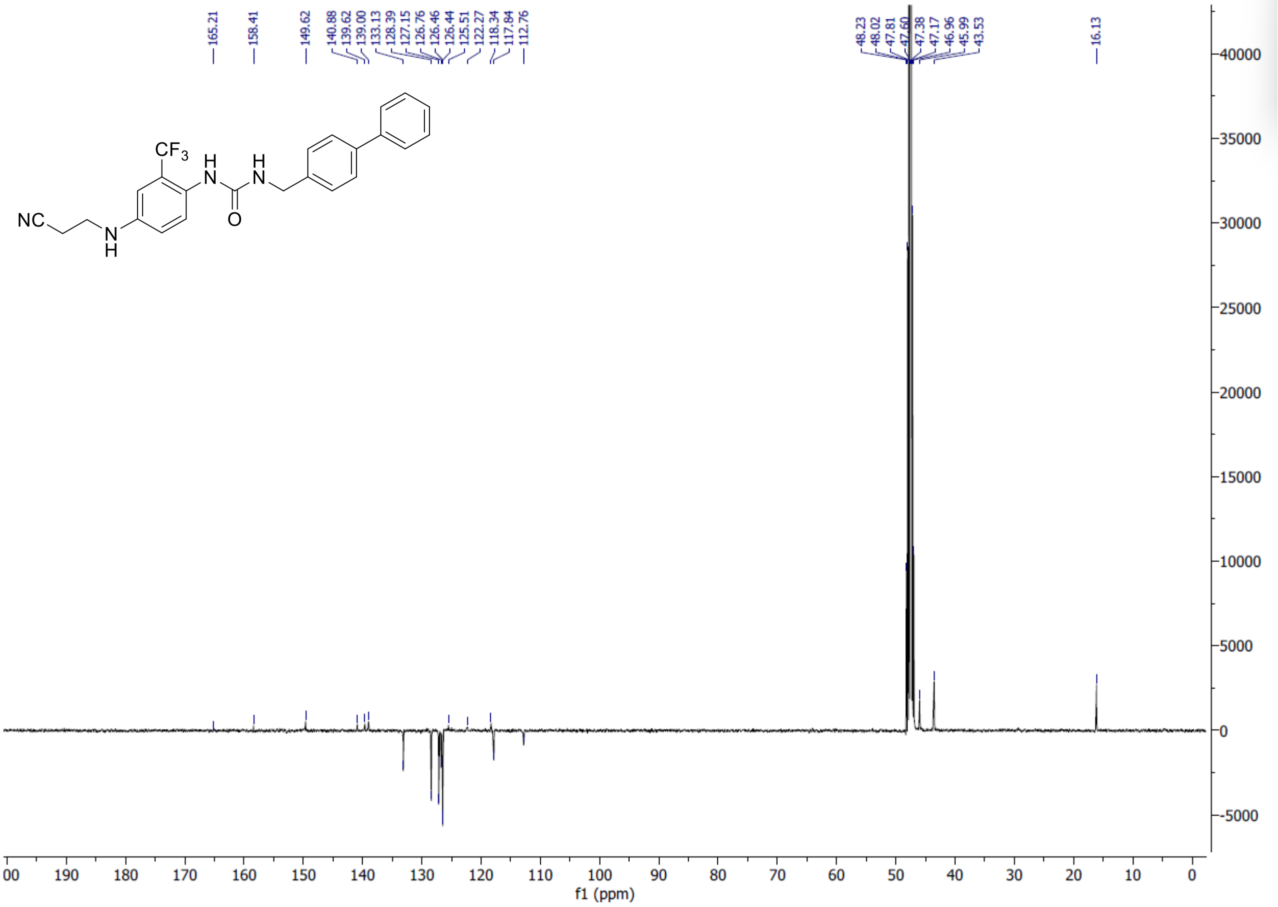

Figure S3: ^{13}C-DEPTq NMR spectrum of compound **10** (MeOD).

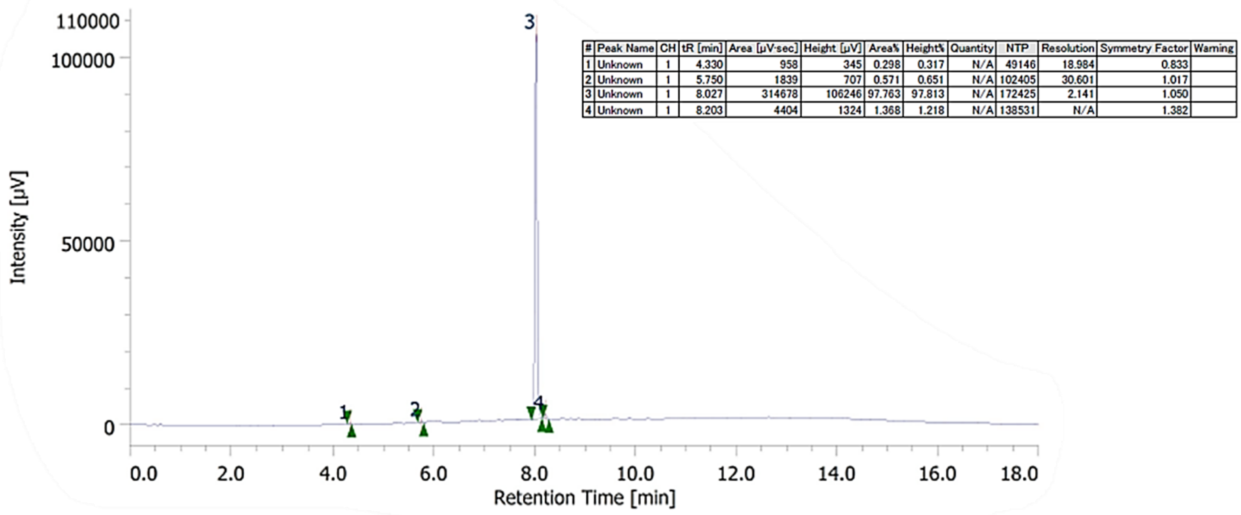

#	Peak Name	CH	tR [min]	Area [μV·sec]	Height [μV]	Area%	Height%	Quantity	NTP	Resolution	Symmetry Factor	Warning
1	Unknown	1	4.330	958	345	0.298	0.317	N/A	49146	18.984	0.833	
2	Unknown	1	5.750	1839	707	0.571	0.651	N/A	102405	30.601	1.017	
3	Unknown	1	8.027	314678	106246	97.763	97.813	N/A	172425	2.141	1.050	
4	Unknown	1	8.203	4404	1324	1.368	1.218	N/A	138531	N/A	1.382	

Figure S4: HPLC trace of compound **10**.

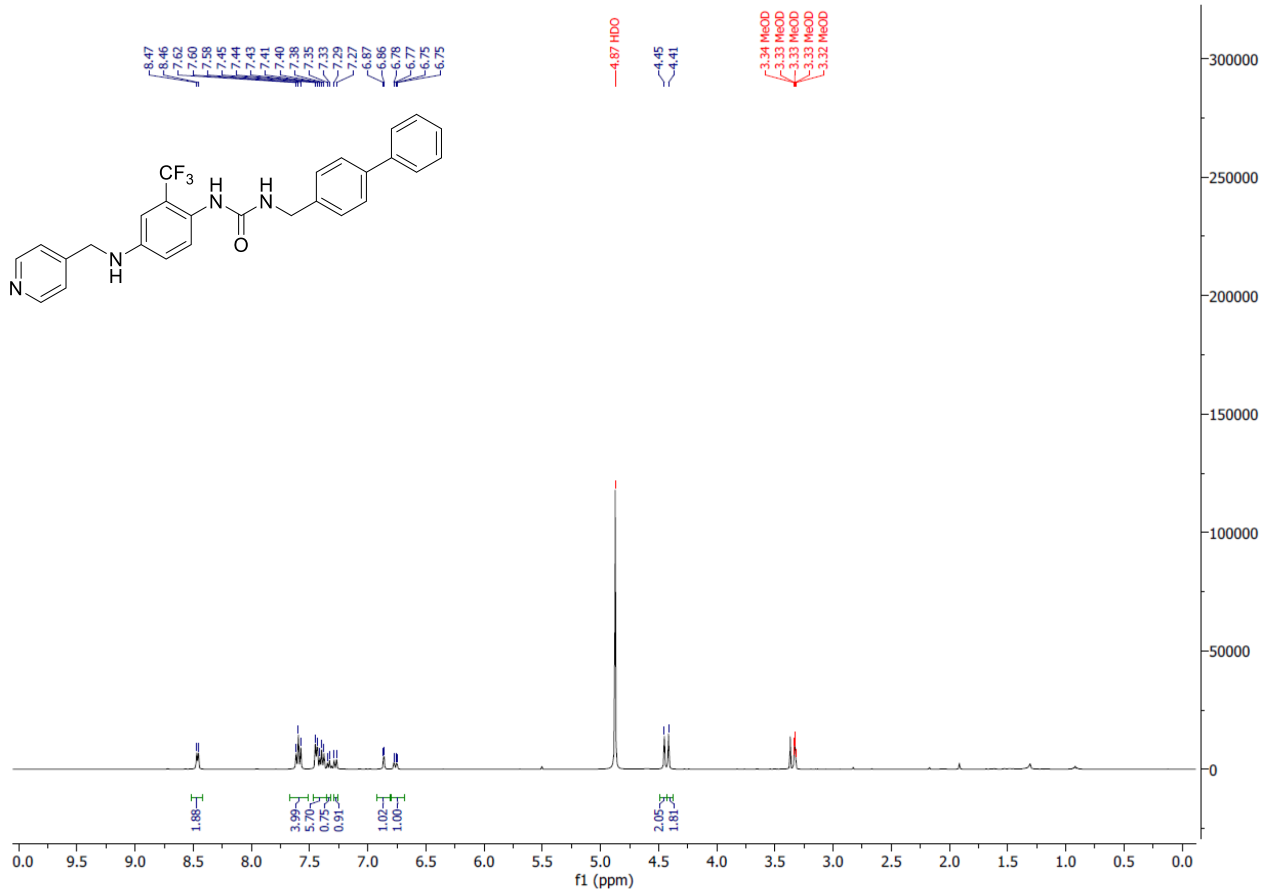

Figure S5: 1H NMR spectrum of compound **13** (MeOD).

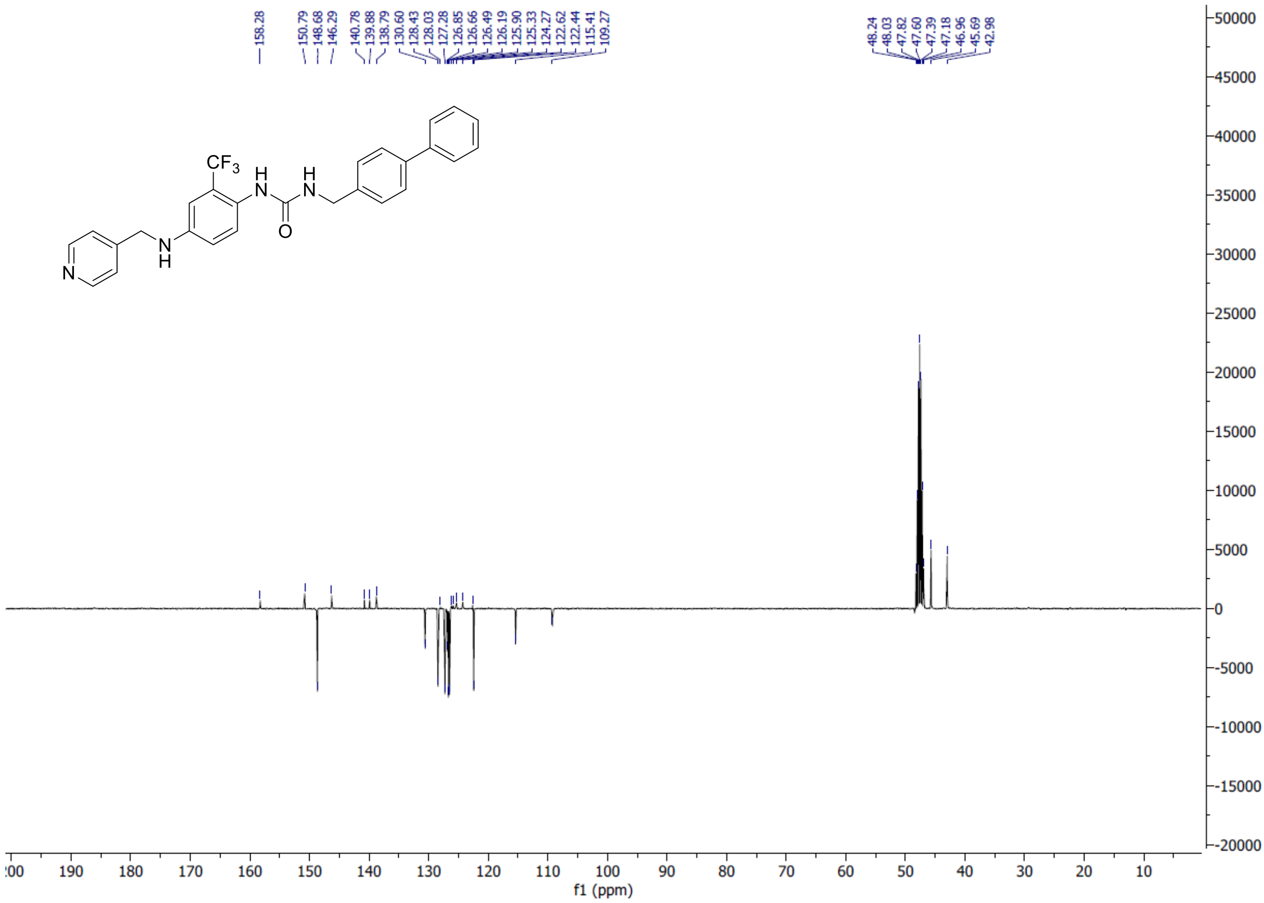

Figure S6: ^{13}C-DEPTq NMR spectrum of compound **13** (MeOD).

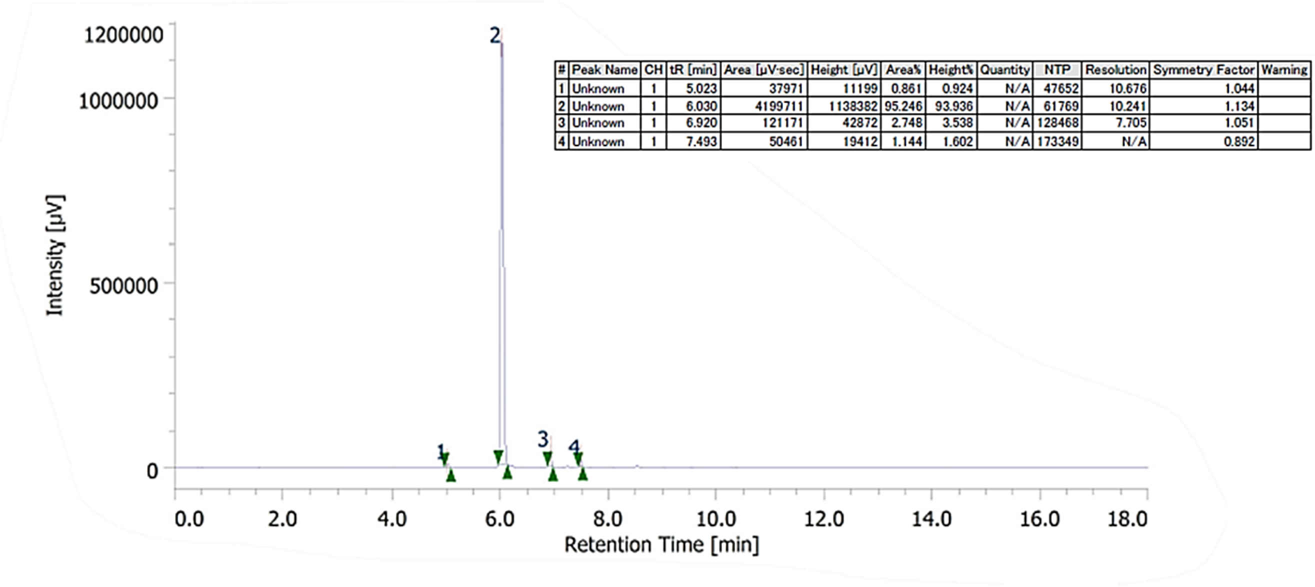

#	Peak Name	CH	tR [min]	Area [µV·sec]	Height [µV]	Area%	Height%	Quantity	NTP	Resolution	Symmetry Factor	Warning
1	Unknown	1	5.023	37971	11199	0.861	0.924	N/A	47652	10.676	1.044	
2	Unknown	1	6.030	4199711	1138382	95.246	93.936	N/A	61769	10.241	1.134	
3	Unknown	1	6.920	121171	42872	2.748	3.538	N/A	128468	7.705	1.051	
4	Unknown	1	7.493	50461	19412	1.144	1.602	N/A	173349	N/A	0.892	

Figure S7: HPLC trace of compound **13**.

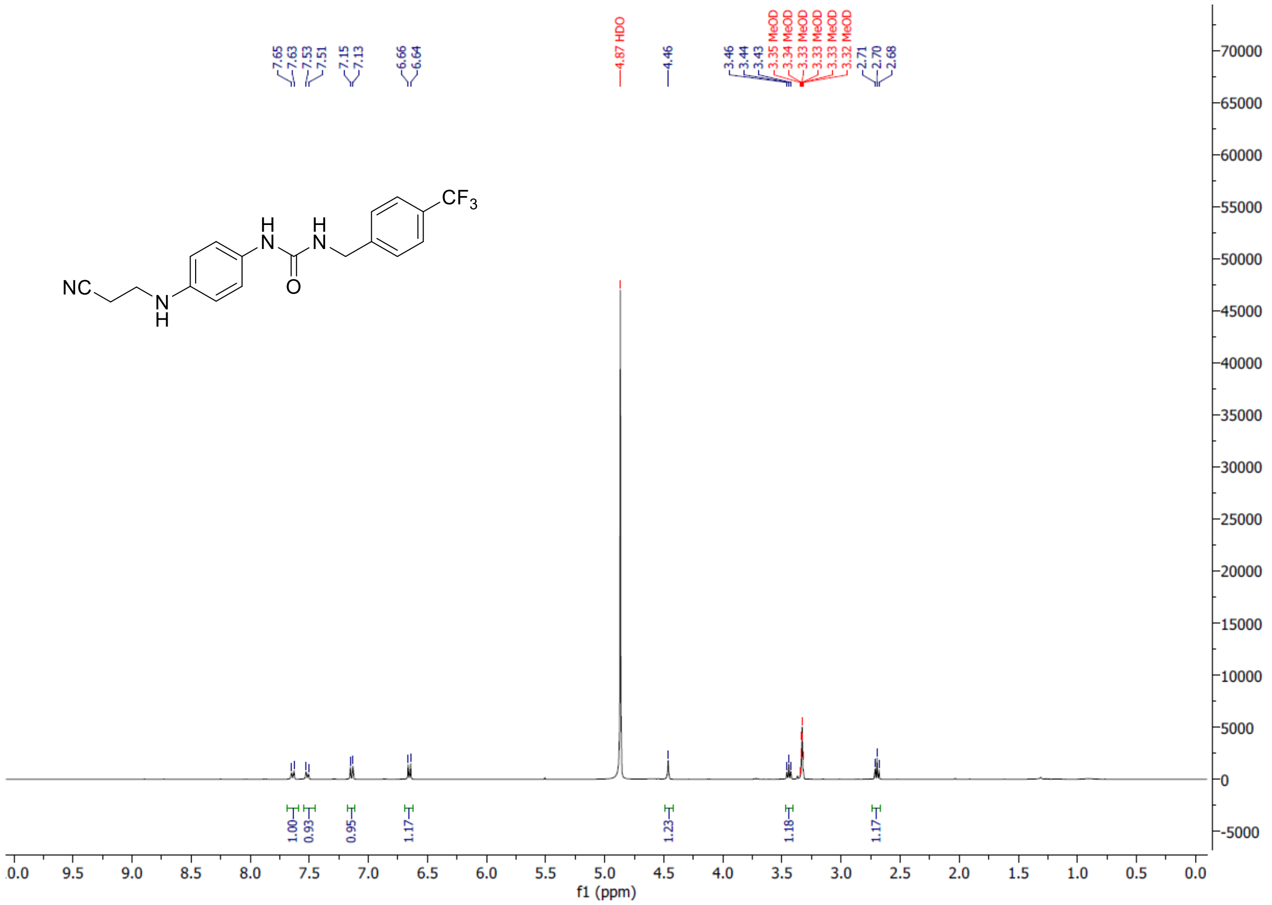

Figure S8: 1H NMR spectrum of compound **14** (MeOD).

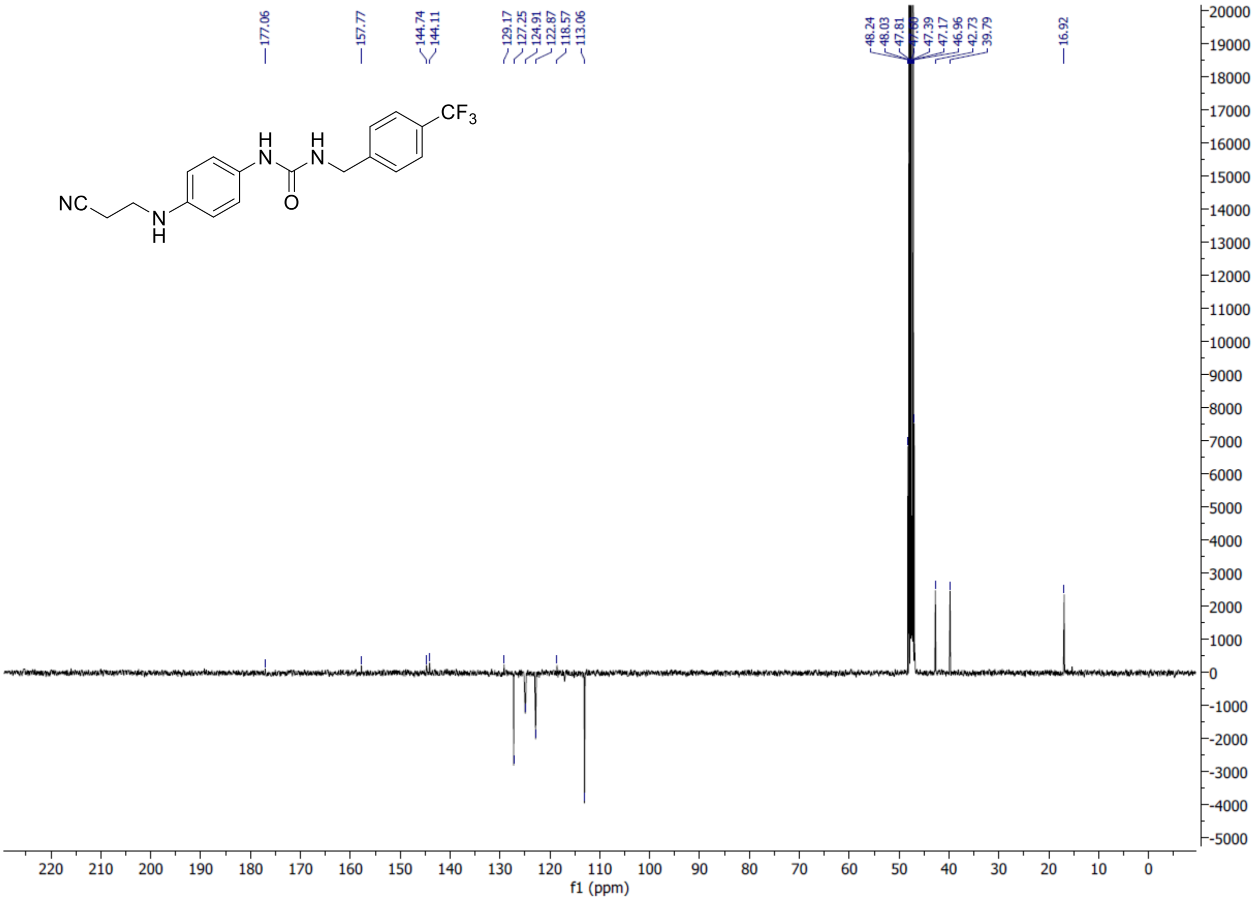

Figure S9: ^{13}C-DEPTq NMR spectrum of compound **14** (MeOD).

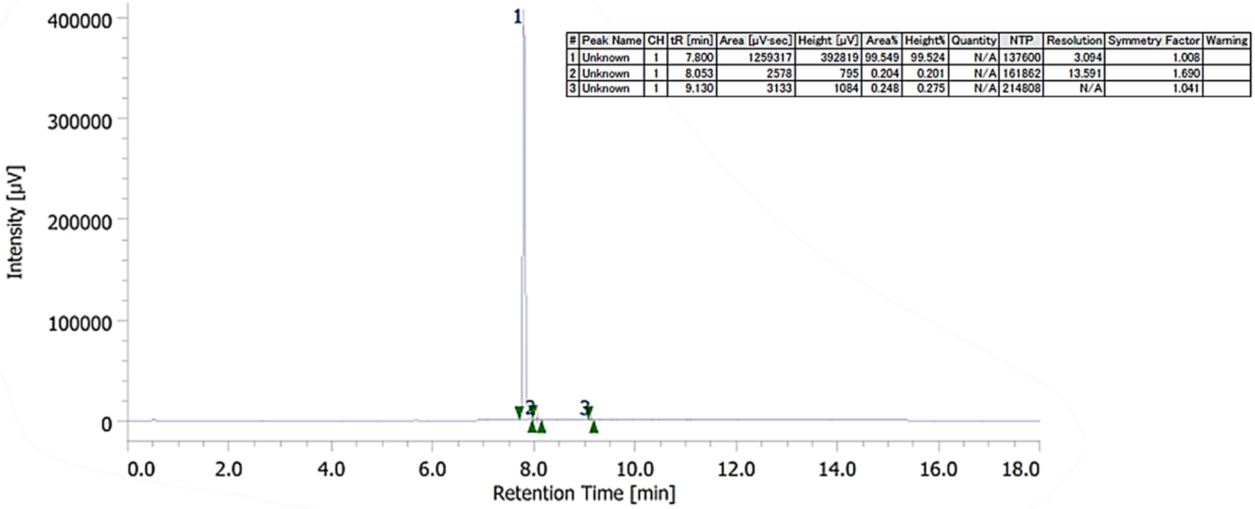

#	Peak Name	CH	tR [min]	Area [µV·sec]	Height [µV]	Area%	Height%	Quantity	NTP	Resolution	Symmetry Factor	Warning
1	Unknown	1	7.800	1259317	392819	99.549	99.524	N/A	137600	3.094	1.008	
2	Unknown	1	8.053	2578	795	0.204	0.201	N/A	161862	13.591	1.690	
3	Unknown	1	9.130	3133	1084	0.248	0.275	N/A	214808	N/A	1.041	

Figure S10: HPLC trace of compound **14**.

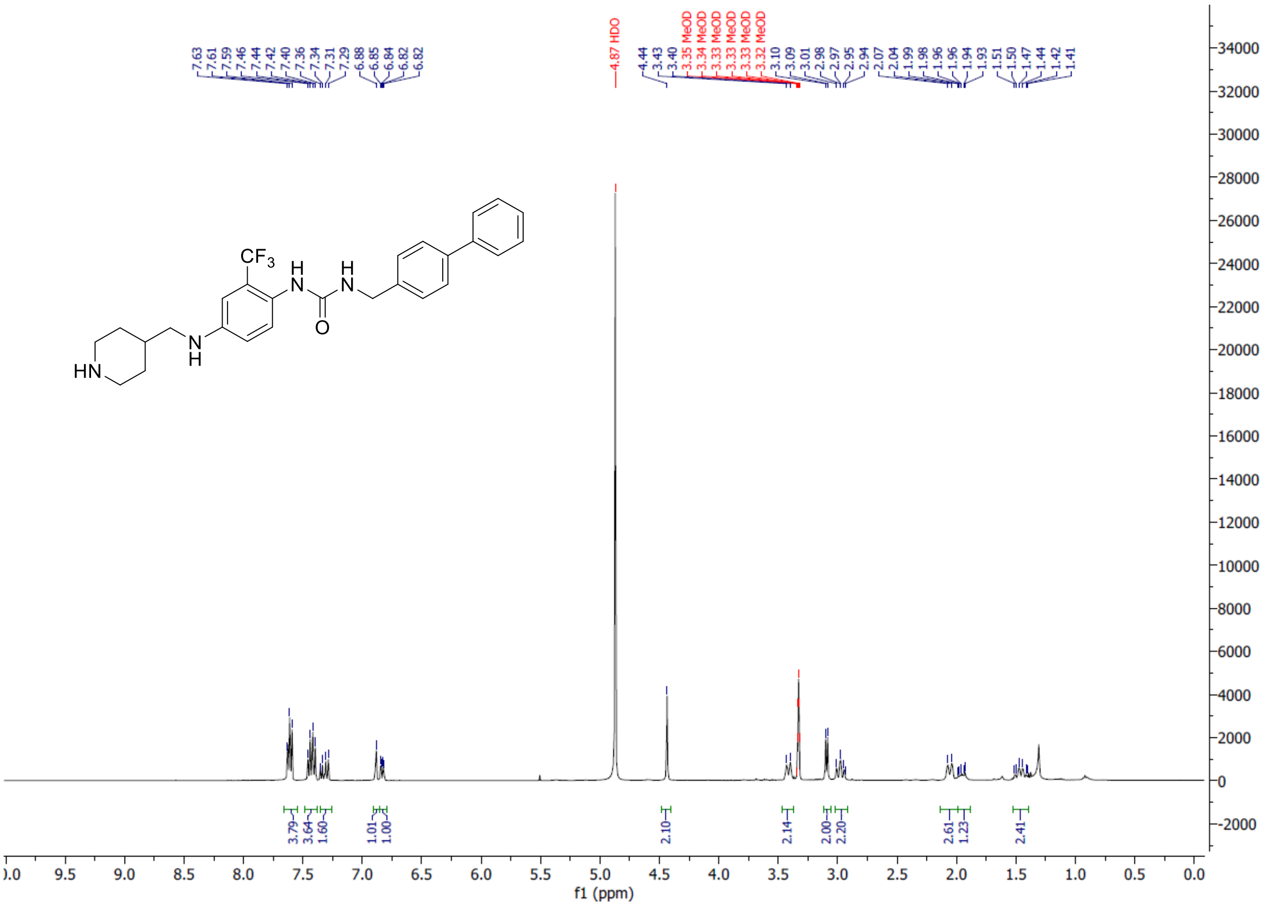

Figure S11: 1H NMR spectrum of compound **17** (MeOD).

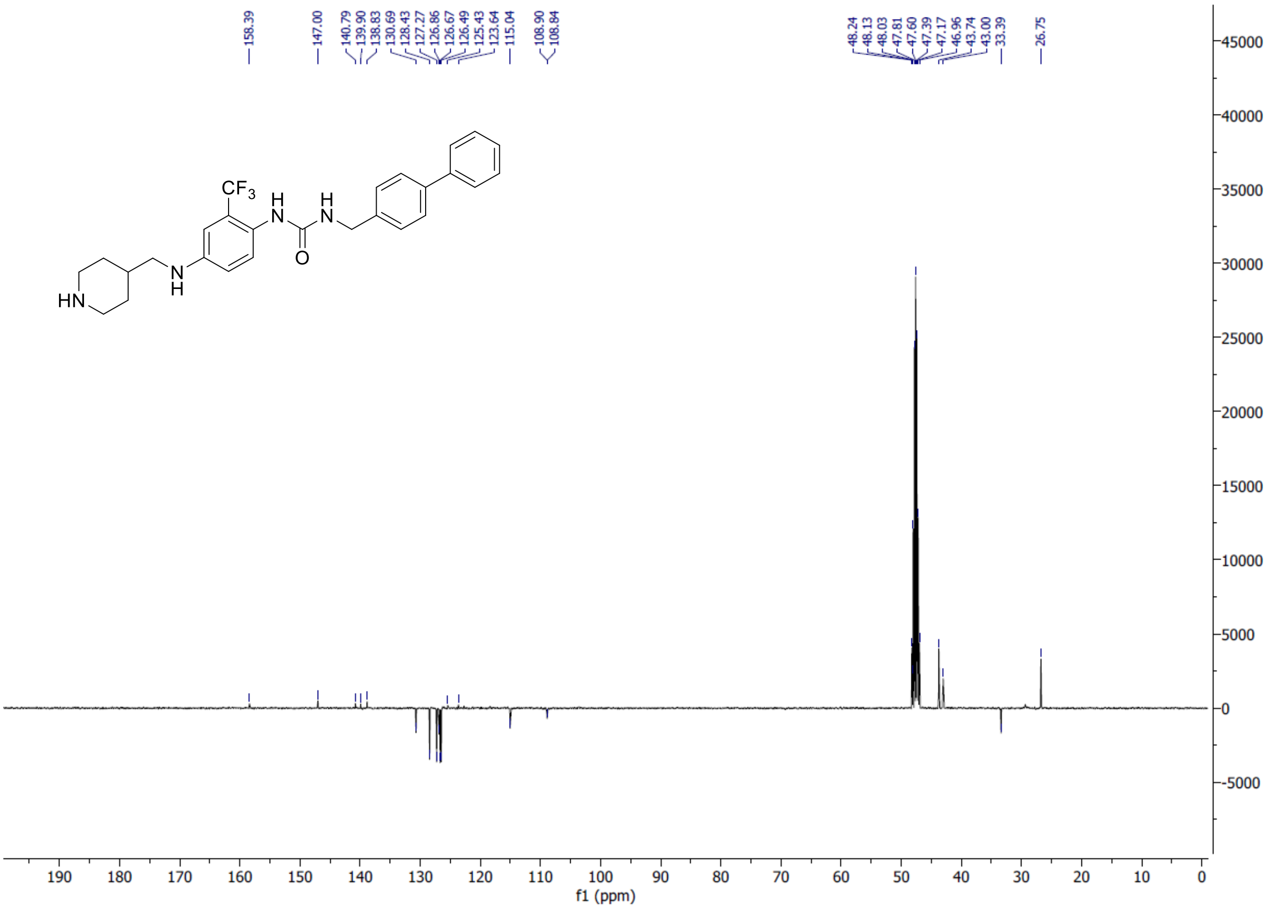

Figure S12: ^{13}C-DEPTq NMR spectrum of compound **17** (MeOD).

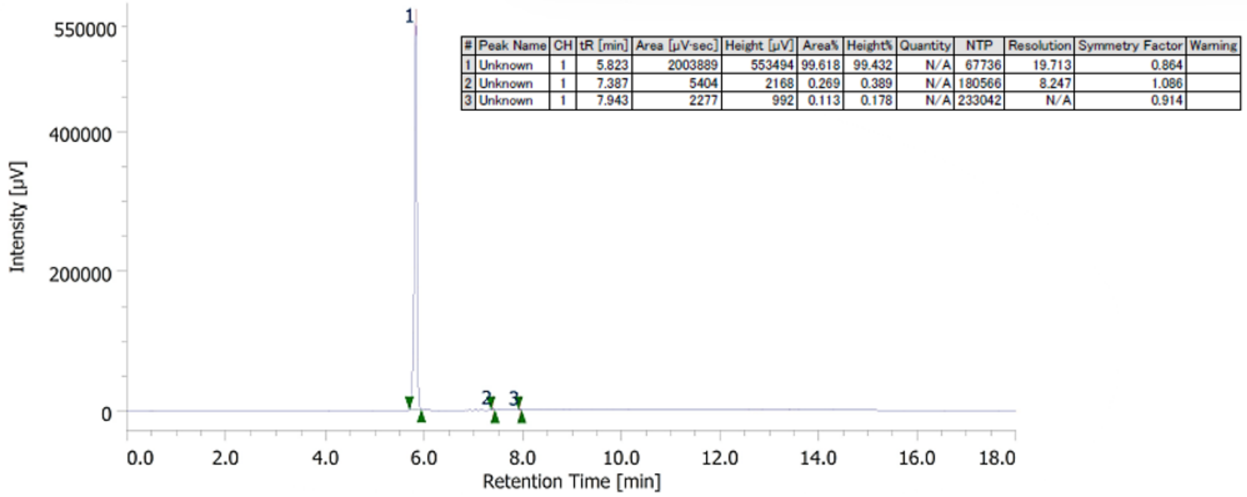

#	Peak Name	CH	tR [min]	Area [µV·sec]	Height [µV]	Area%	Height%	Quantity	NTP	Resolution	Symmetry Factor	Warning
1	Unknown	1	5.823	2003889	553494	99.618	99.432	N/A	67736	19.713	0.864	
2	Unknown	1	7.387	5404	2168	0.269	0.389	N/A	180566	8.247	1.086	
3	Unknown	1	7.943	2277	992	0.113	0.178	N/A	233042	N/A	0.914	

Figure S13: HPLC trace compound **17**.

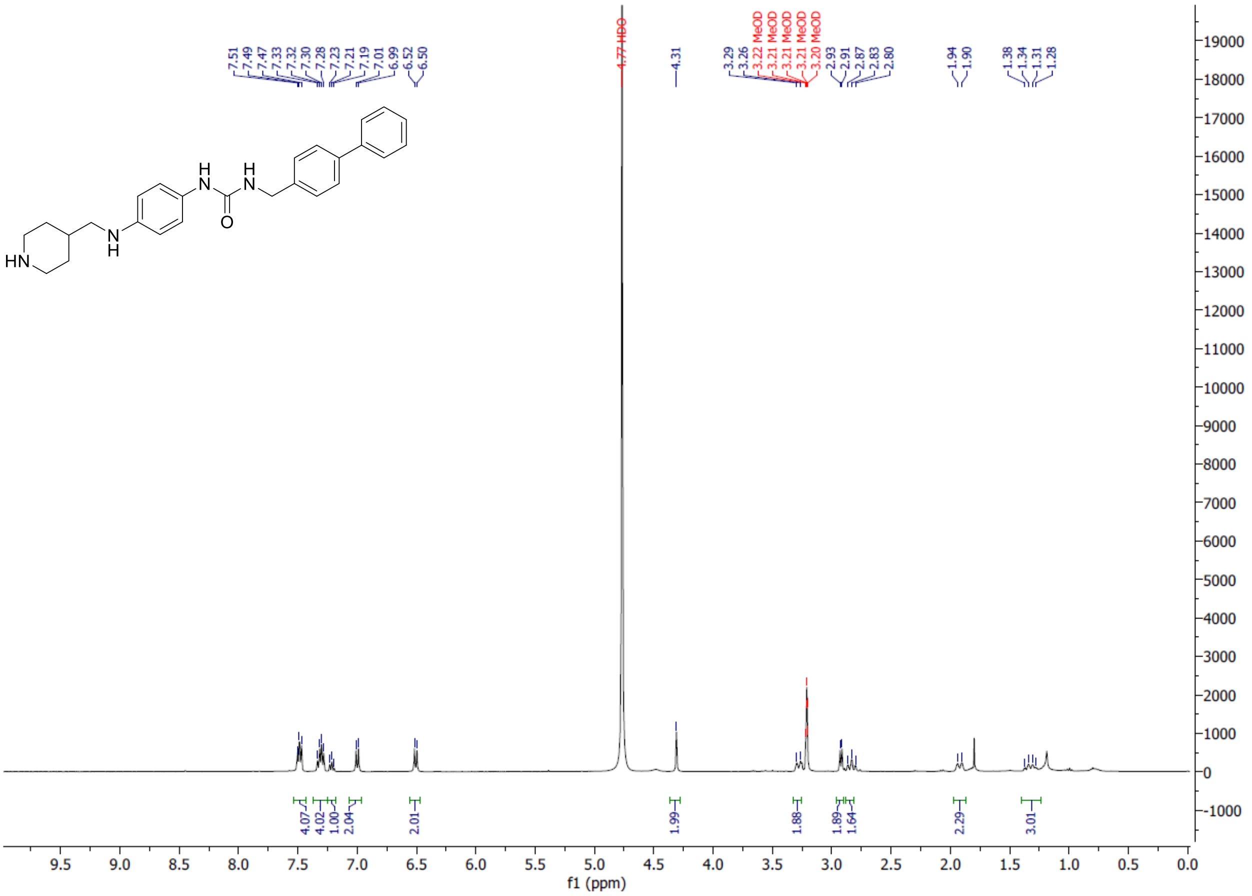

Figure S14: 1H NMR spectrum of compound **18** (MeOD).

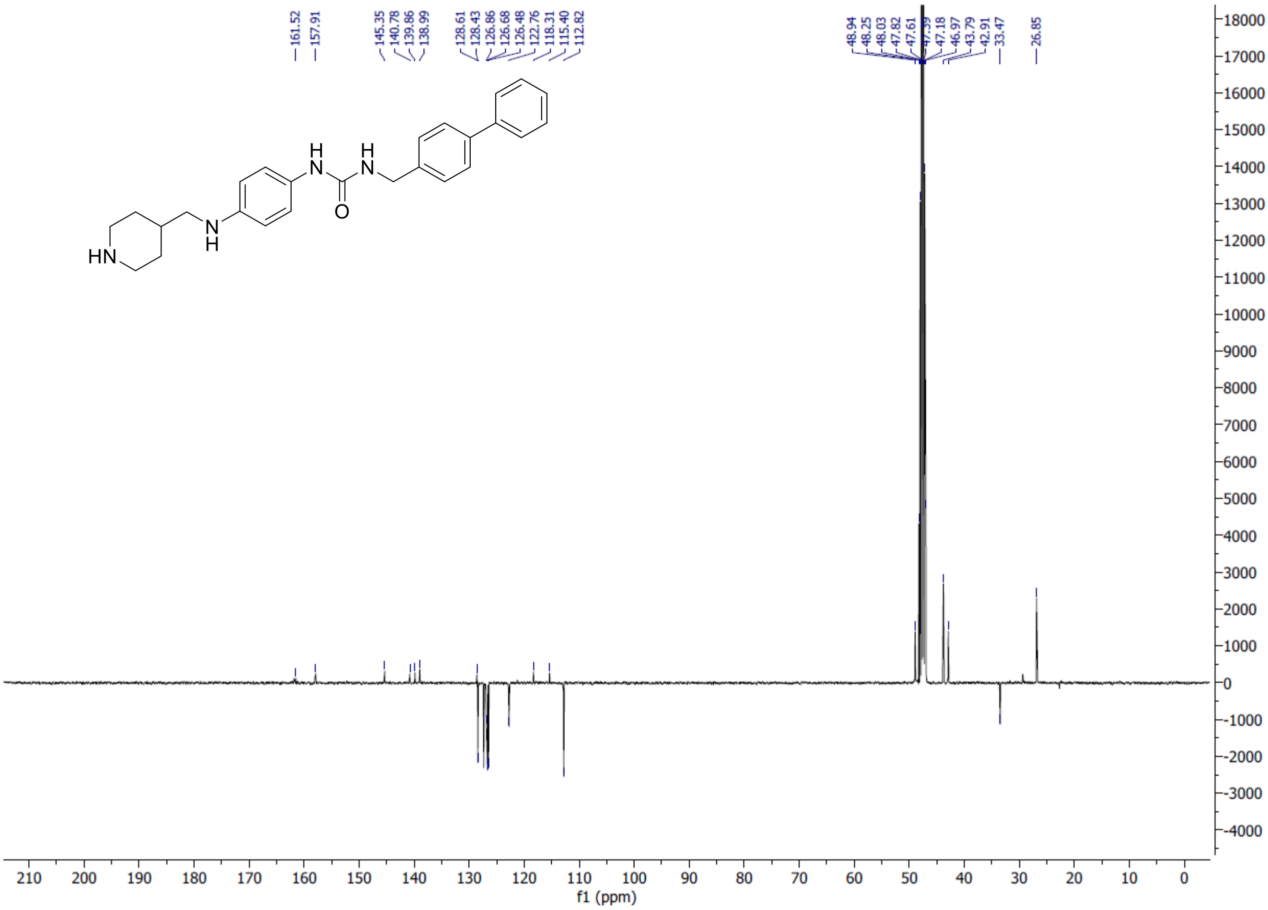

Figure S15: ^{13}C-DEPTq NMR spectrum of compound **18** (MeOD).

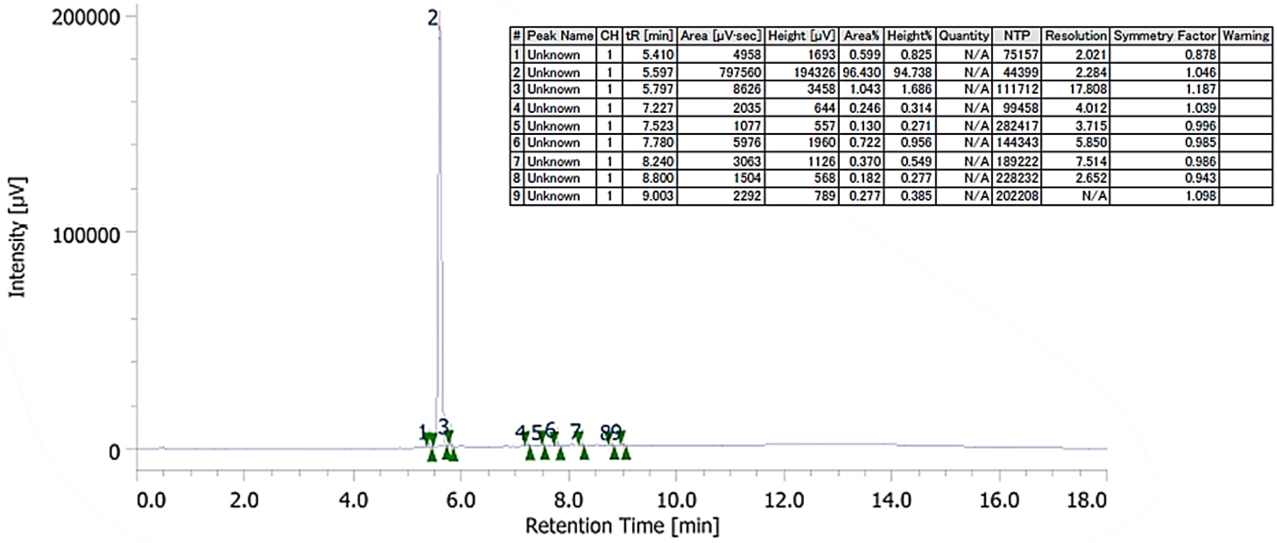

#	Peak Name	CH	tR [min]	Area [µV·sec]	Height [µV]	Area%	Height%	Quantity	NTP	Resolution	Symmetry Factor	Warning
1	Unknown	1	5.410	4958	1693	0.599	0.825	N/A	75157	2.021	0.878	
2	Unknown	1	5.597	797560	194326	96.430	94.738	N/A	44399	2.284	1.046	
3	Unknown	1	5.797	8626	3458	1.043	1.686	N/A	111712	17.808	1.187	
4	Unknown	1	7.227	2035	644	0.246	0.314	N/A	99458	4.012	1.039	
5	Unknown	1	7.523	1077	557	0.130	0.271	N/A	282417	3.715	0.996	
6	Unknown	1	7.780	5976	1960	0.722	0.956	N/A	144343	5.850	0.985	
7	Unknown	1	8.240	3063	1126	0.370	0.549	N/A	189222	7.514	0.986	
8	Unknown	1	8.800	1504	568	0.182	0.277	N/A	228232	2.652	0.943	
9	Unknown	1	9.003	2292	789	0.277	0.385	N/A	202208	N/A	1.098	

Figure S16: HPLC trace of compound **18**.

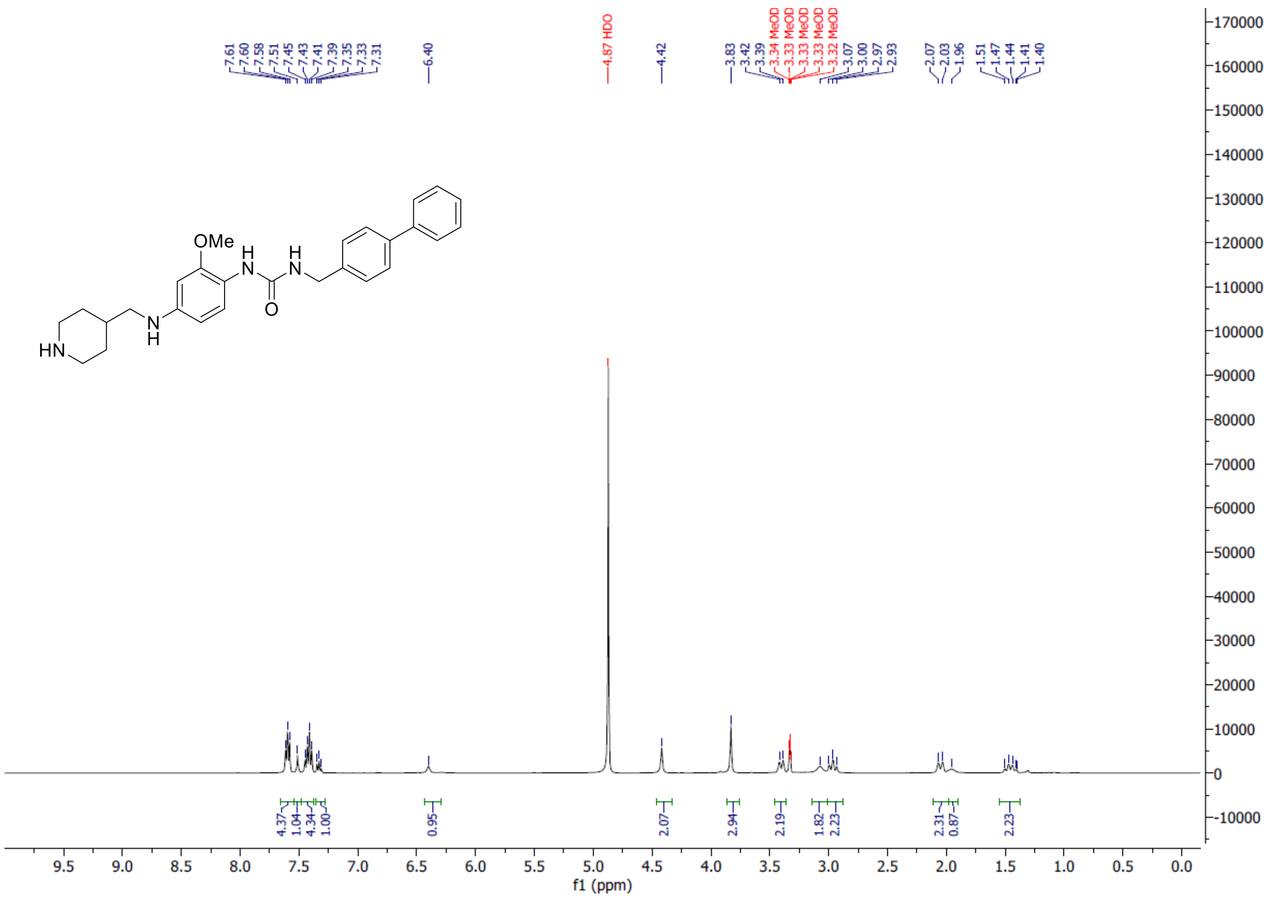

Figure S17: 1H NMR spectrum of compound **19** (MeOD).

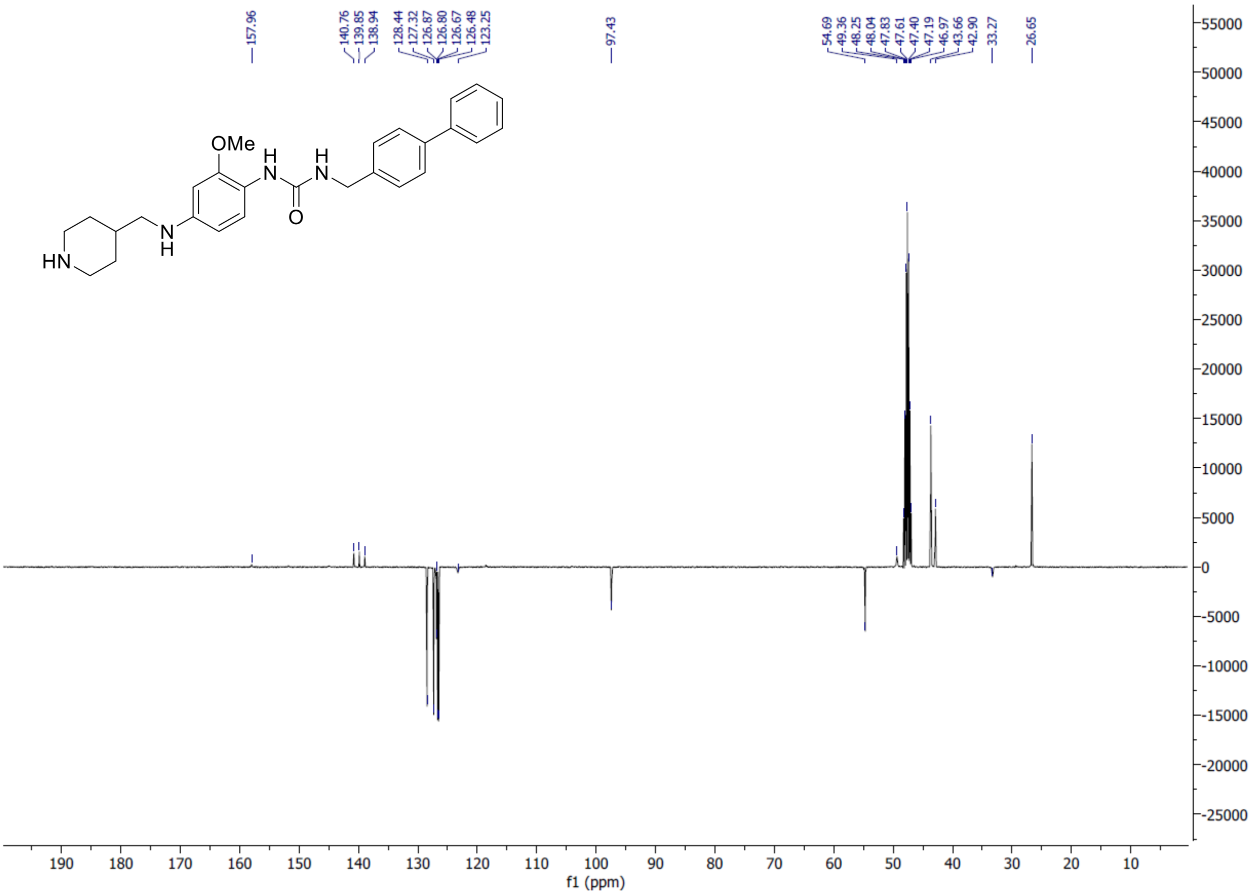

Figure S18: ^{13}C-DEPTq NMR spectrum of compound **19** (MeOD).

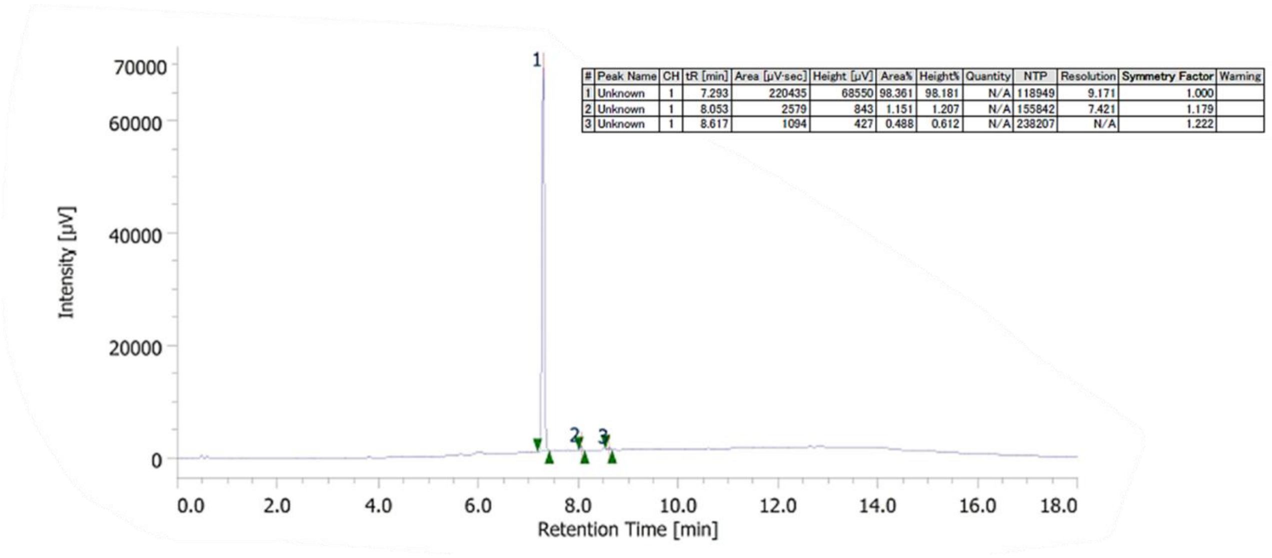

#	Peak Name	CH	tR [min]	Area [µV·sec]	Height [µV]	Area%	Height%	Quantity	NTP	Resolution	Symmetry Factor	Warning
1	Unknown	1	7.293	220435	68550	98.361	98.181	N/A	118949	9.171	1.000	
2	Unknown	1	8.053	2579	843	1.151	1.207	N/A	155842	7.421	1.179	
3	Unknown	1	8.617	1094	427	0.488	0.612	N/A	238207	N/A	1.222	

Figure S19: HPLC trace of compound **19**.

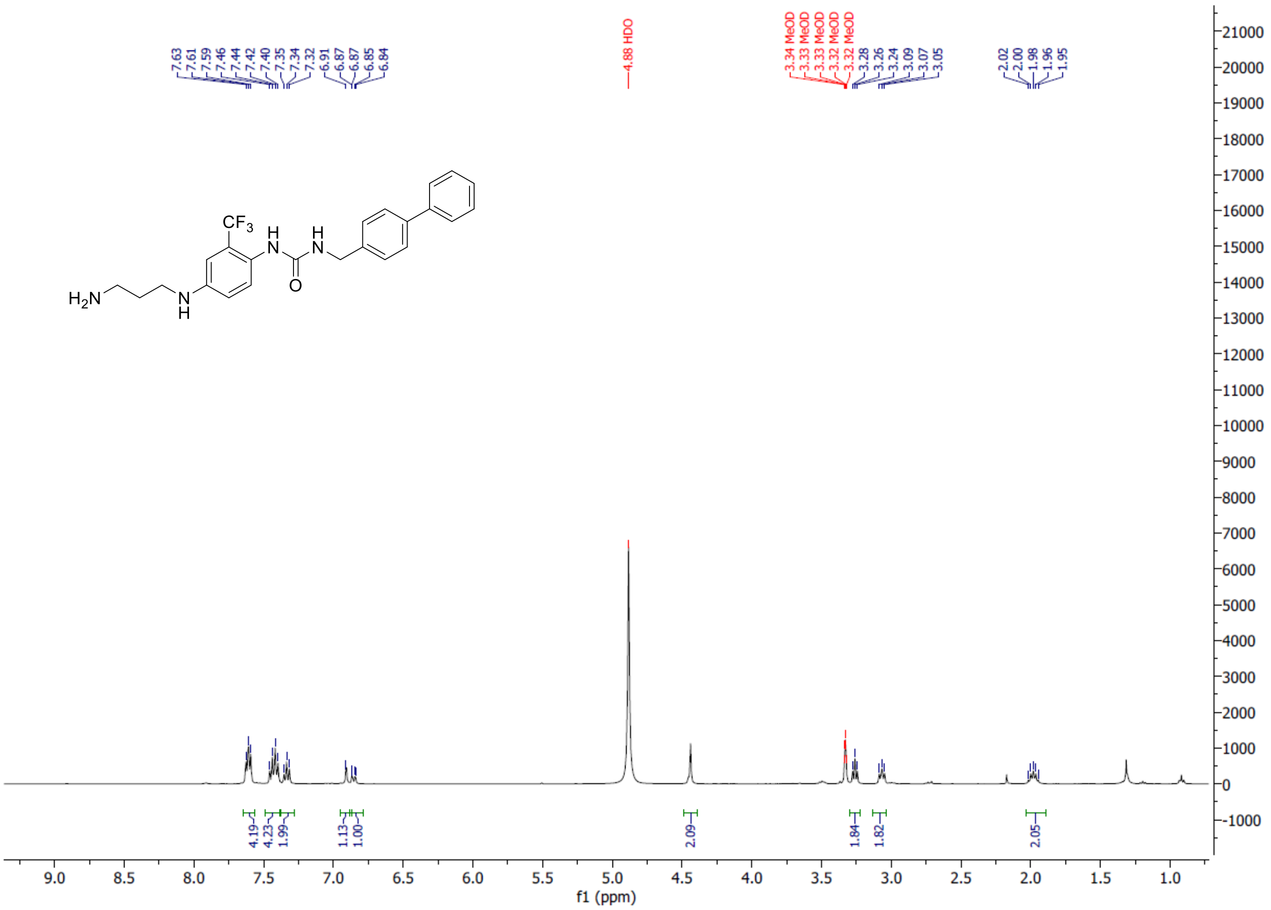

Figure S20: 1H NMR spectrum of compound **20** (MeOD).

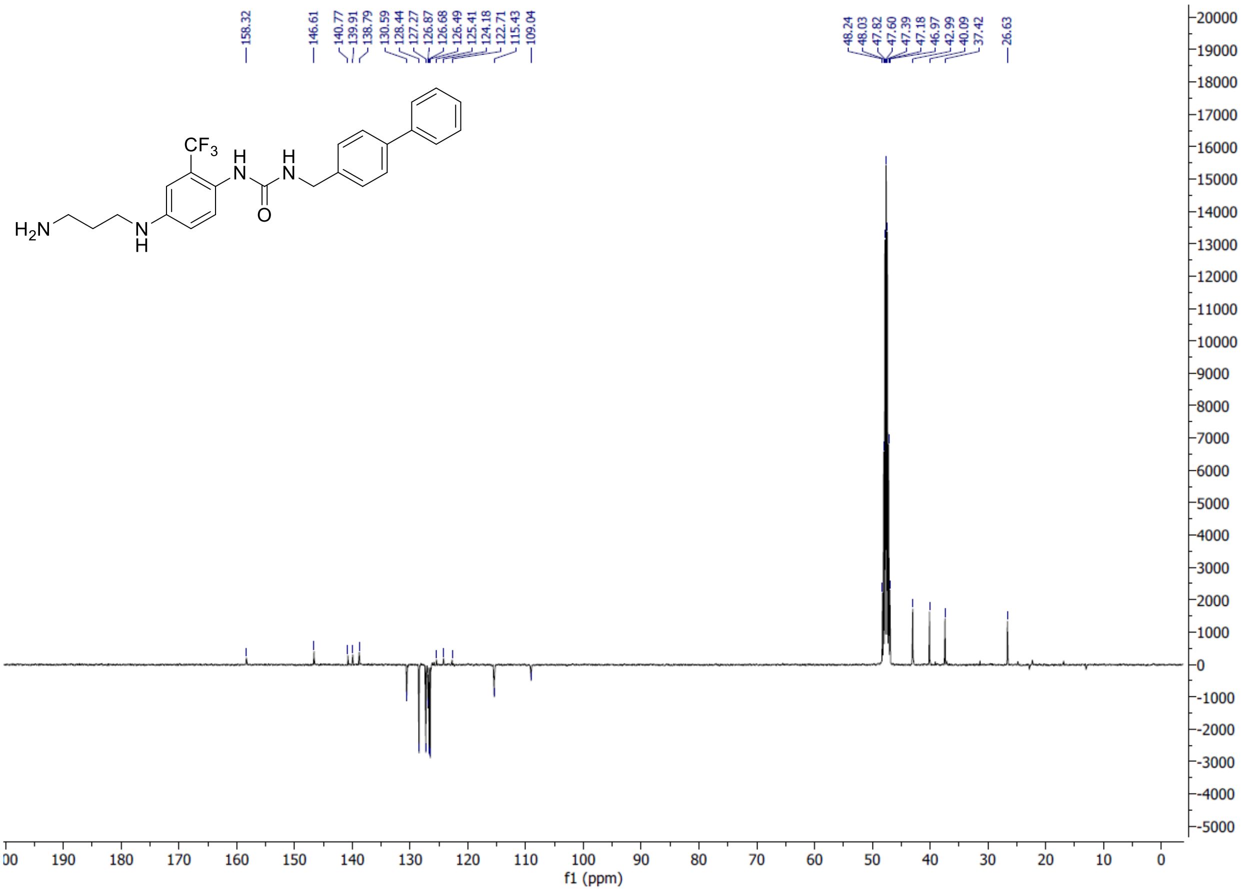

Figure S21: ^{13}C-DEPTq NMR spectrum of compound **20** (MeOD).

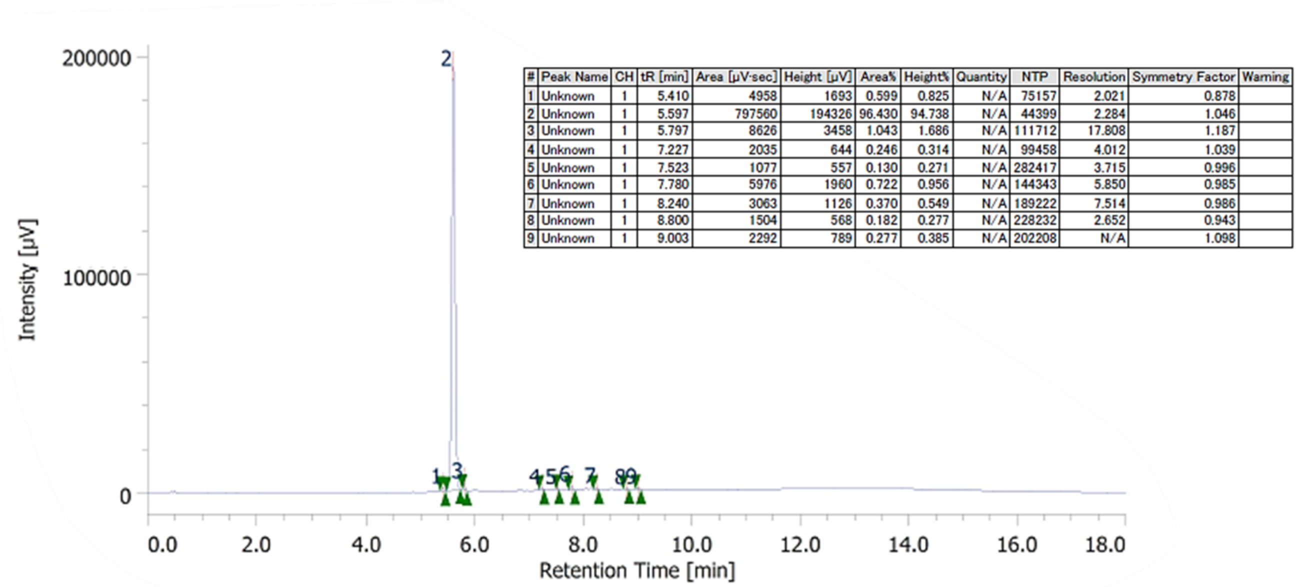

#	Peak Name	CH	tR [min]	Area [µV·sec]	Height [µV]	Area%	Height%	Quantity	NTP	Resolution	Symmetry Factor	Warning
1	Unknown	1	5.410	4958	1693	0.599	0.825	N/A	75157	2.021	0.878	
2	Unknown	1	5.597	797560	194326	96.430	94.738	N/A	44399	2.284	1.046	
3	Unknown	1	5.797	8626	3458	1.043	1.686	N/A	111712	17.808	1.187	
4	Unknown	1	7.227	2035	644	0.246	0.314	N/A	99458	4.012	1.039	
5	Unknown	1	7.523	1077	557	0.130	0.271	N/A	282417	3.715	0.996	
6	Unknown	1	7.780	5976	1960	0.722	0.956	N/A	144343	5.850	0.985	
7	Unknown	1	8.240	3063	1126	0.370	0.549	N/A	189222	7.514	0.986	
8	Unknown	1	8.800	1504	568	0.182	0.277	N/A	228232	2.652	0.943	
9	Unknown	1	9.003	2292	789	0.277	0.385	N/A	202208	N/A	1.098	

Figure S22: HPLC trace of compound **20**.

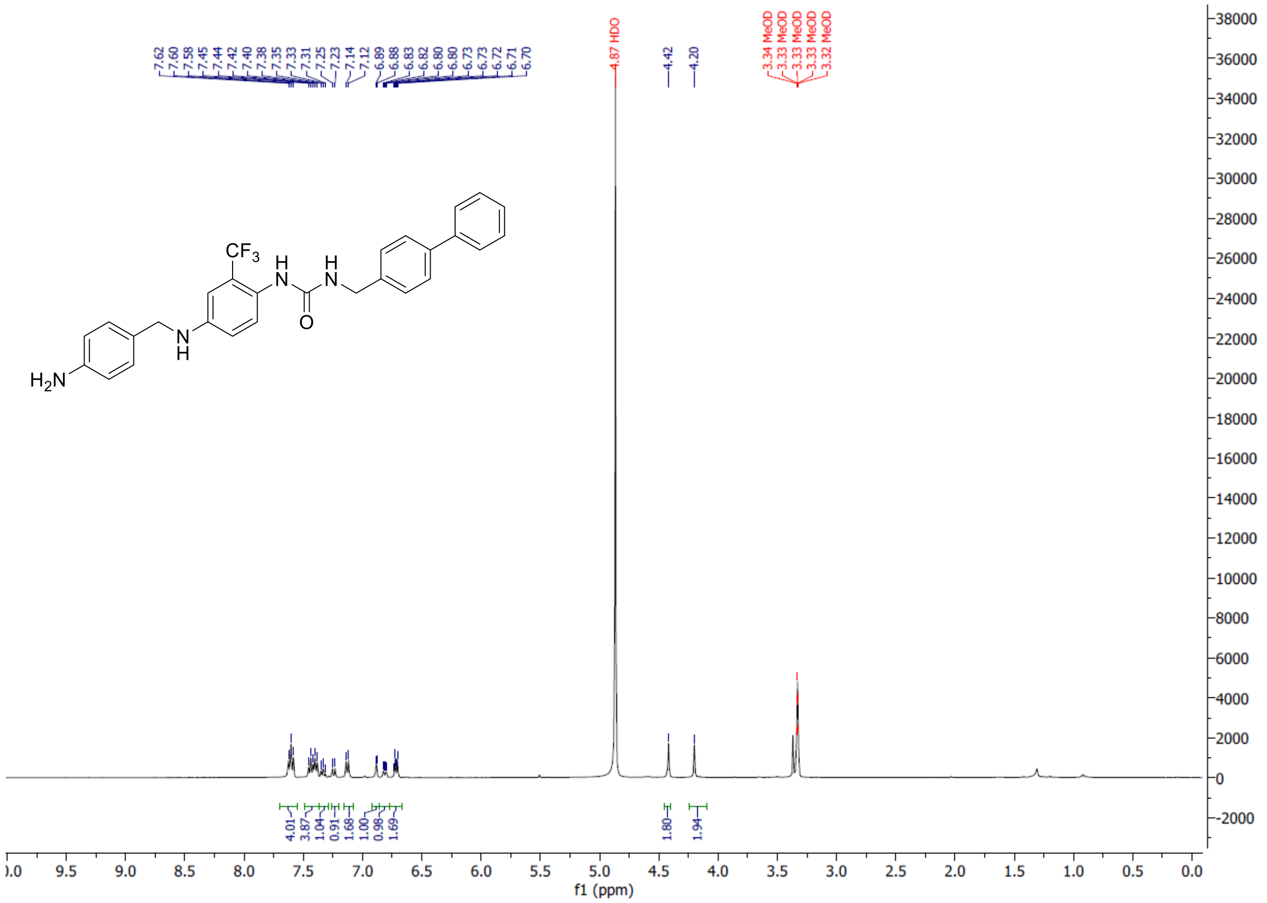

Figure S23: 1H NMR spectrum of compound **21** (MeOD).

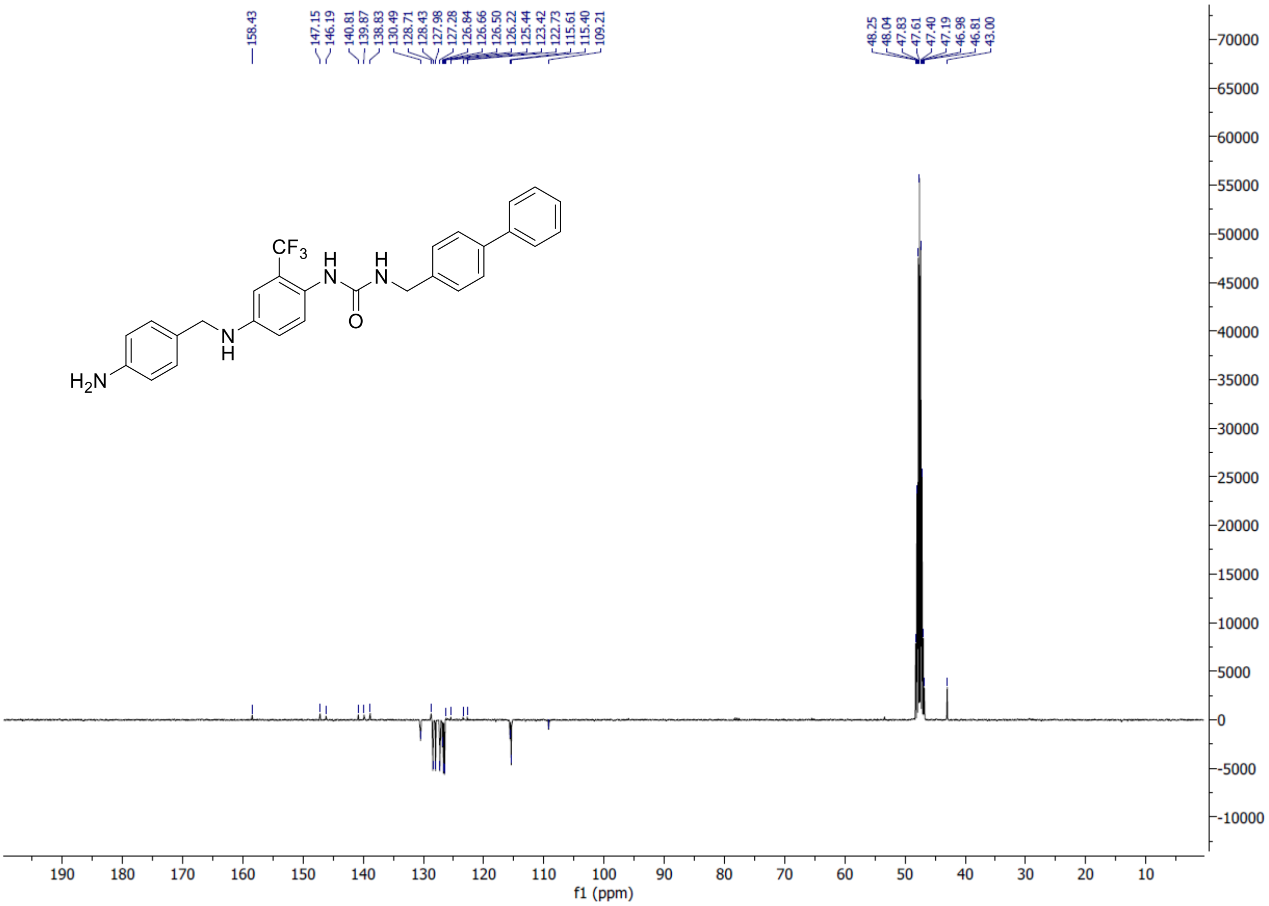

Figure S24: ^{13}C-DEPTq NMR spectrum of compound **21** (MeOD).

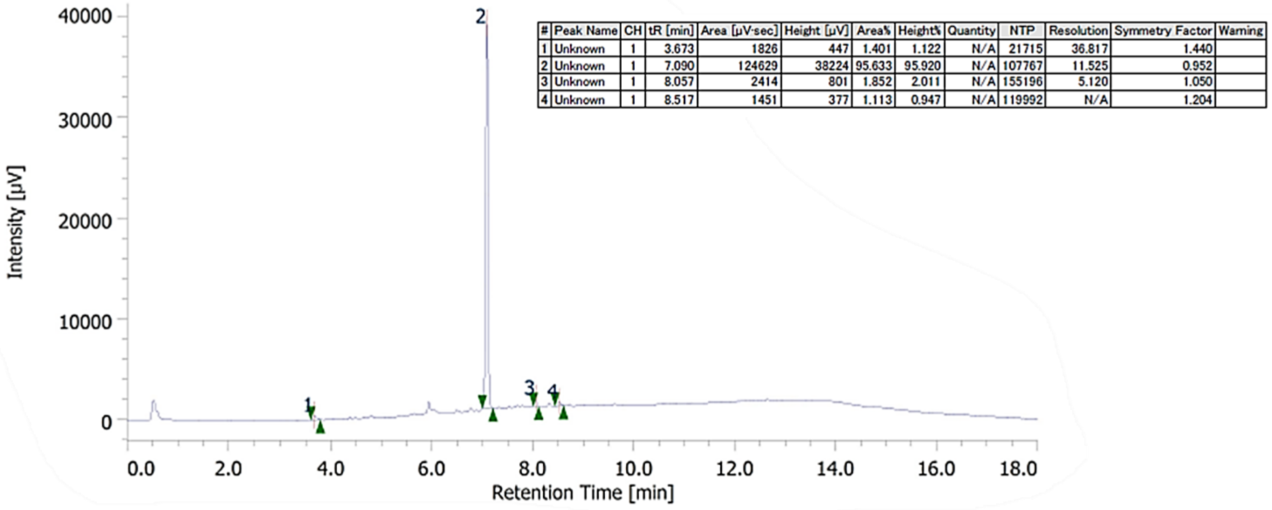

#	Peak Name	CH	tR [min]	Area [µV·sec]	Height [µV]	Area%	Height%	Quantity	NTP	Resolution	Symmetry Factor	Warning
1	Unknown	1	3.673	1826	447	1.401	1.122	N/A	21715	36.817	1.440	
2	Unknown	1	7.090	124629	38224	95.633	95.920	N/A	107767	11.525	0.952	
3	Unknown	1	8.057	2414	801	1.852	2.011	N/A	155196	5.120	1.050	
4	Unknown	1	8.517	1451	377	1.113	0.947	N/A	119992	N/A	1.204	

Figure S25: HPLC trace of compound **21**.

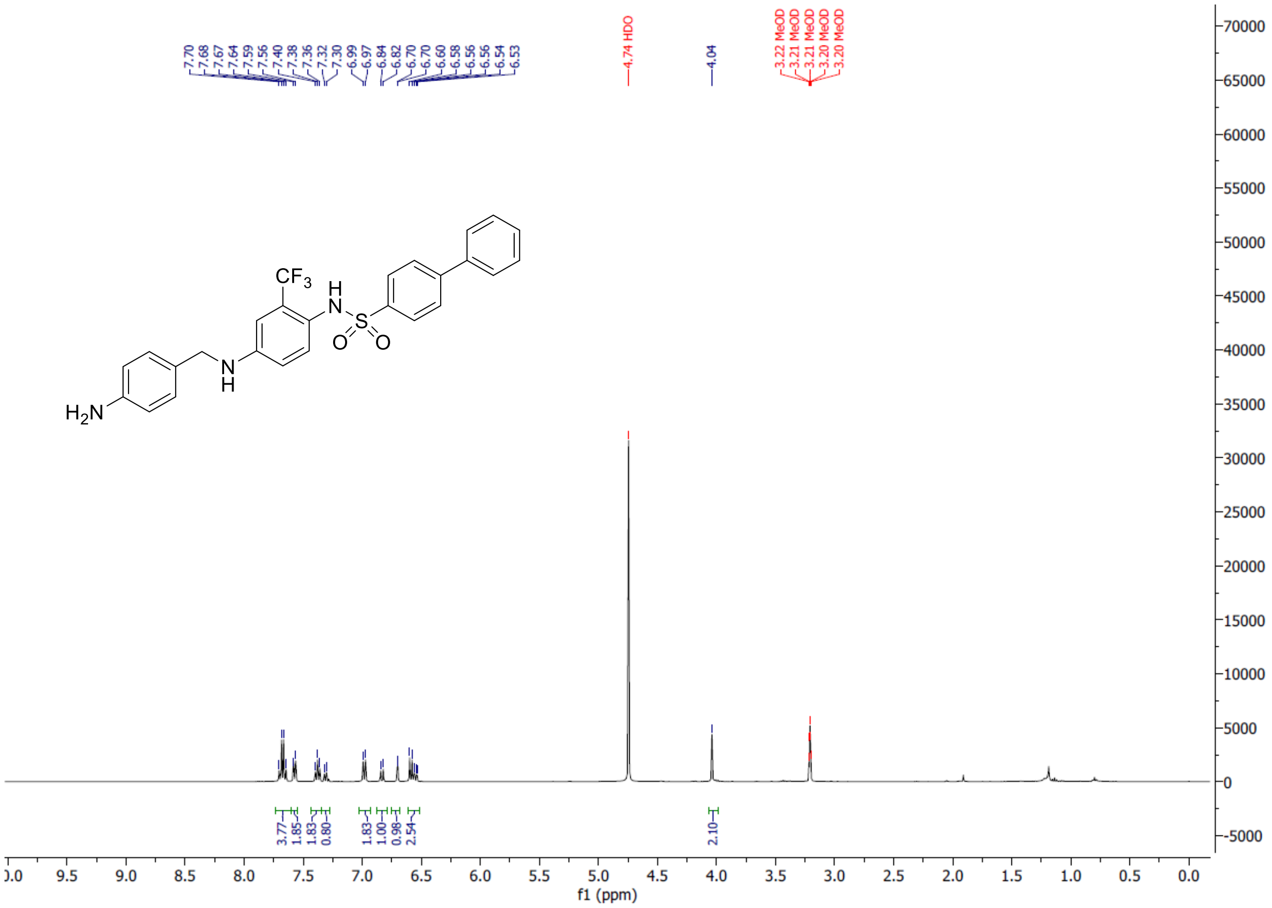

Figure S26: 1H NMR spectrum of compound **26** (MeOD).

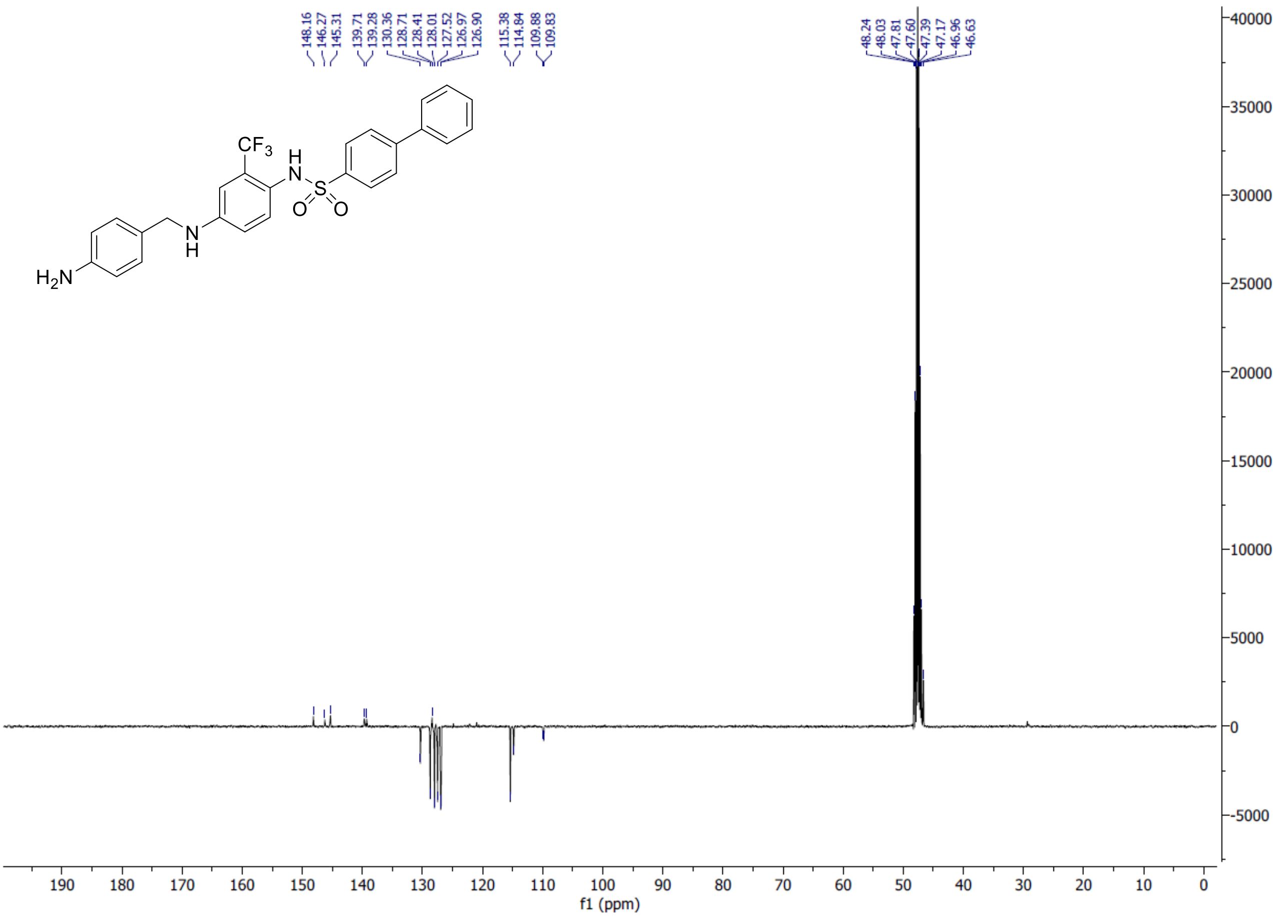

Figure S27: ^{13}C-DEPTq NMR spectrum of compound **26** (MeOD).

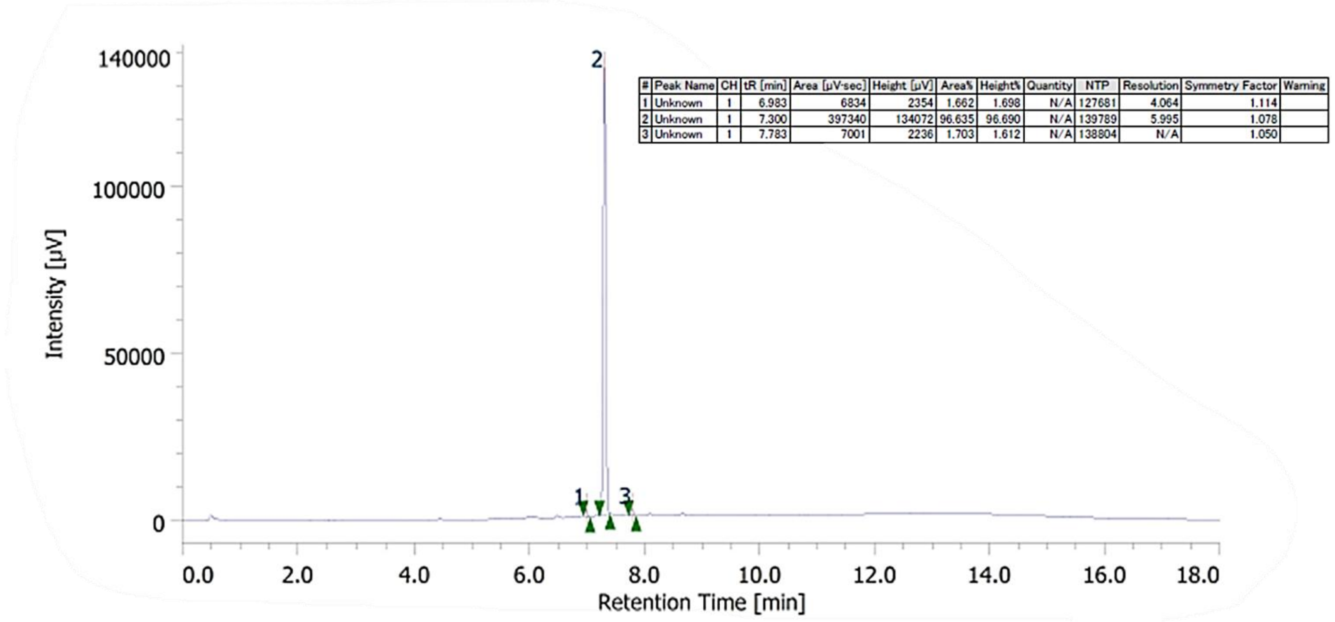

#	Peak Name	CH	tR [min]	Area [µV·sec]	Height [µV]	Area%	Height%	Quantity	NTP	Resolution	Symmetry Factor	Warning
1	Unknown	1	6.983	6834	2354	1.662	1.698	N/A	127681	4.064	1.114	
2	Unknown	1	7.300	397340	134072	96.635	96.690	N/A	139789	5.995	1.078	
3	Unknown	1	7.783	7001	2236	1.703	1.612	N/A	138804	N/A	1.050	

Figure S28: HPLC trace of compound **26**.

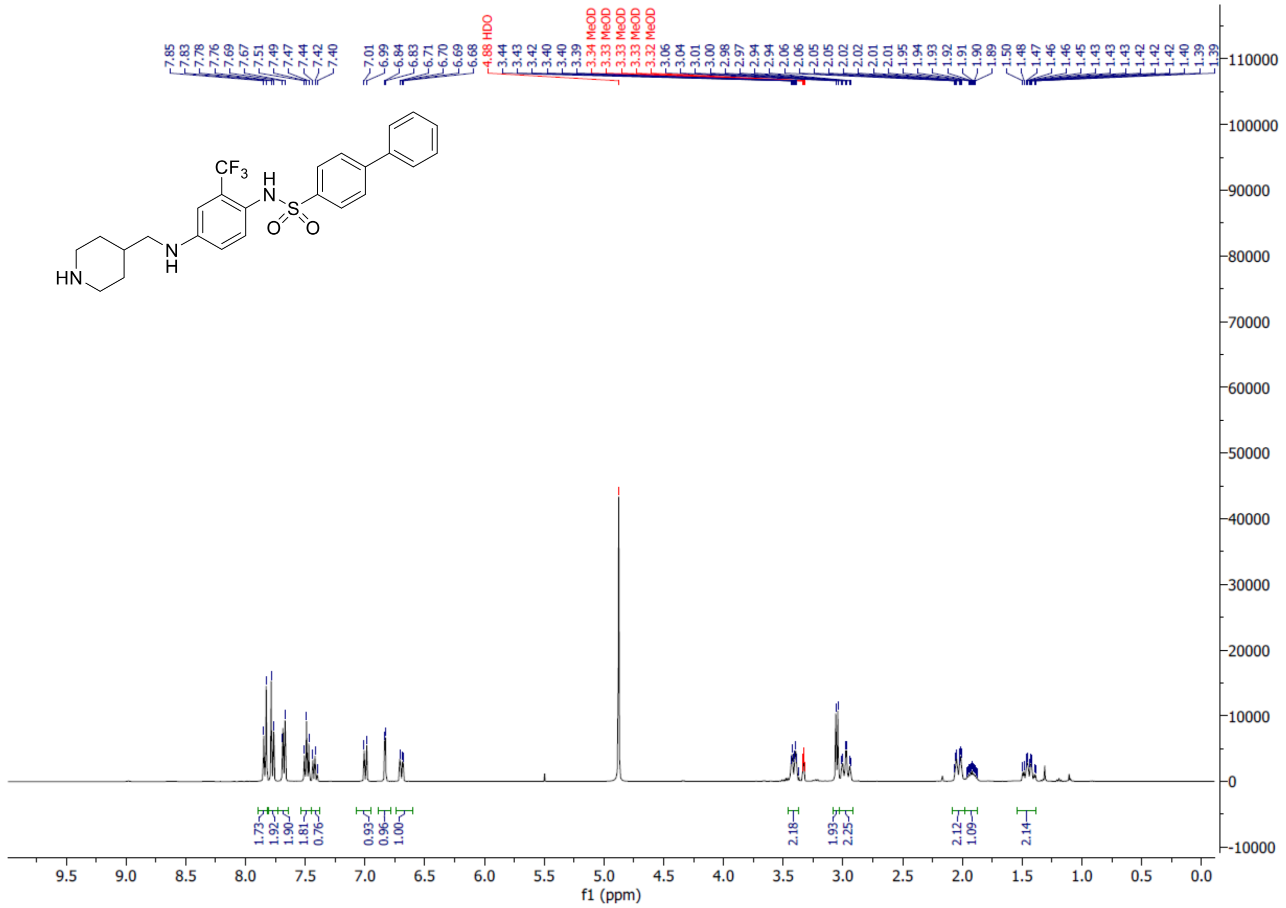

Figure S29: 1H NMR spectrum of compound **27** (MeOD).

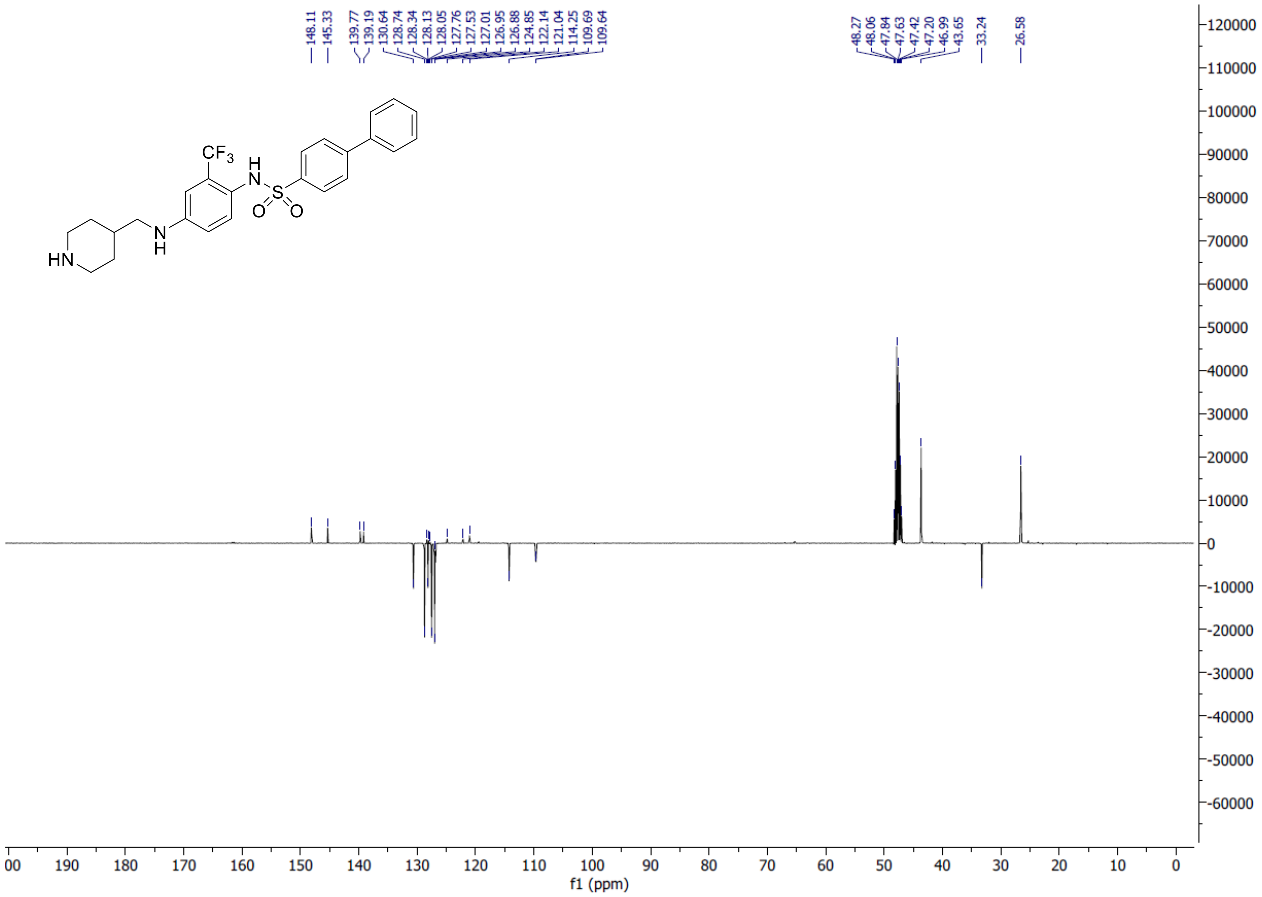

Figure S30: ^{13}C-DEPTq NMR spectrum of compound **27** (MeOD).

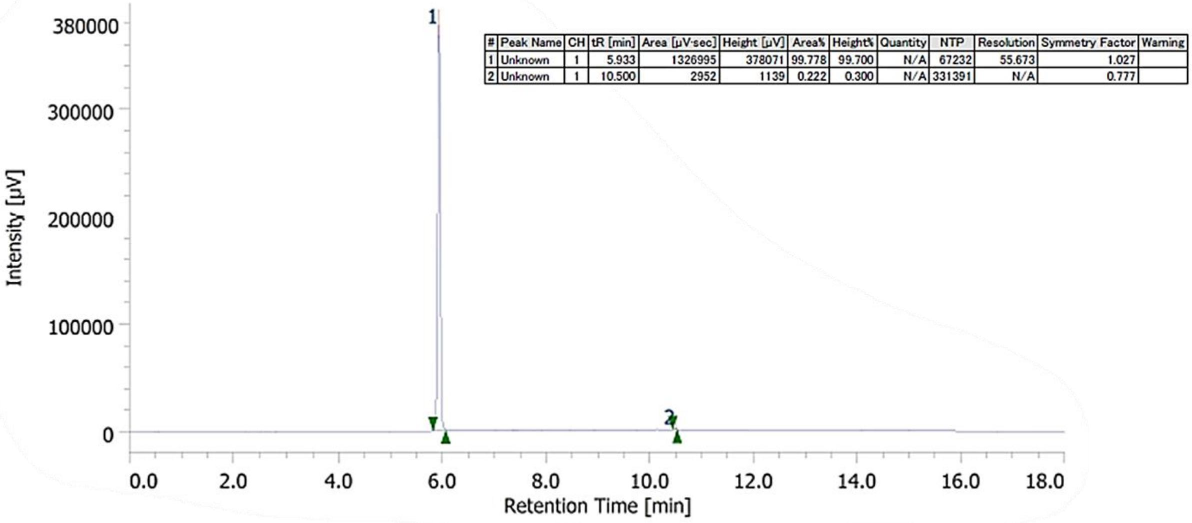

#	Peak Name	CH	tR [min]	Area [µV·sec]	Height [µV]	Area%	Height%	Quantity	NTP	Resolution	Symmetry Factor	Warning
1	Unknown	1	5.933	1326995	378071	99.778	99.700	N/A	67232	55.673	1.027	
2	Unknown	1	10.500	2952	1139	0.222	0.300	N/A	331391	N/A	0.777	

Figure S31: HPLC trace of compound **27**.

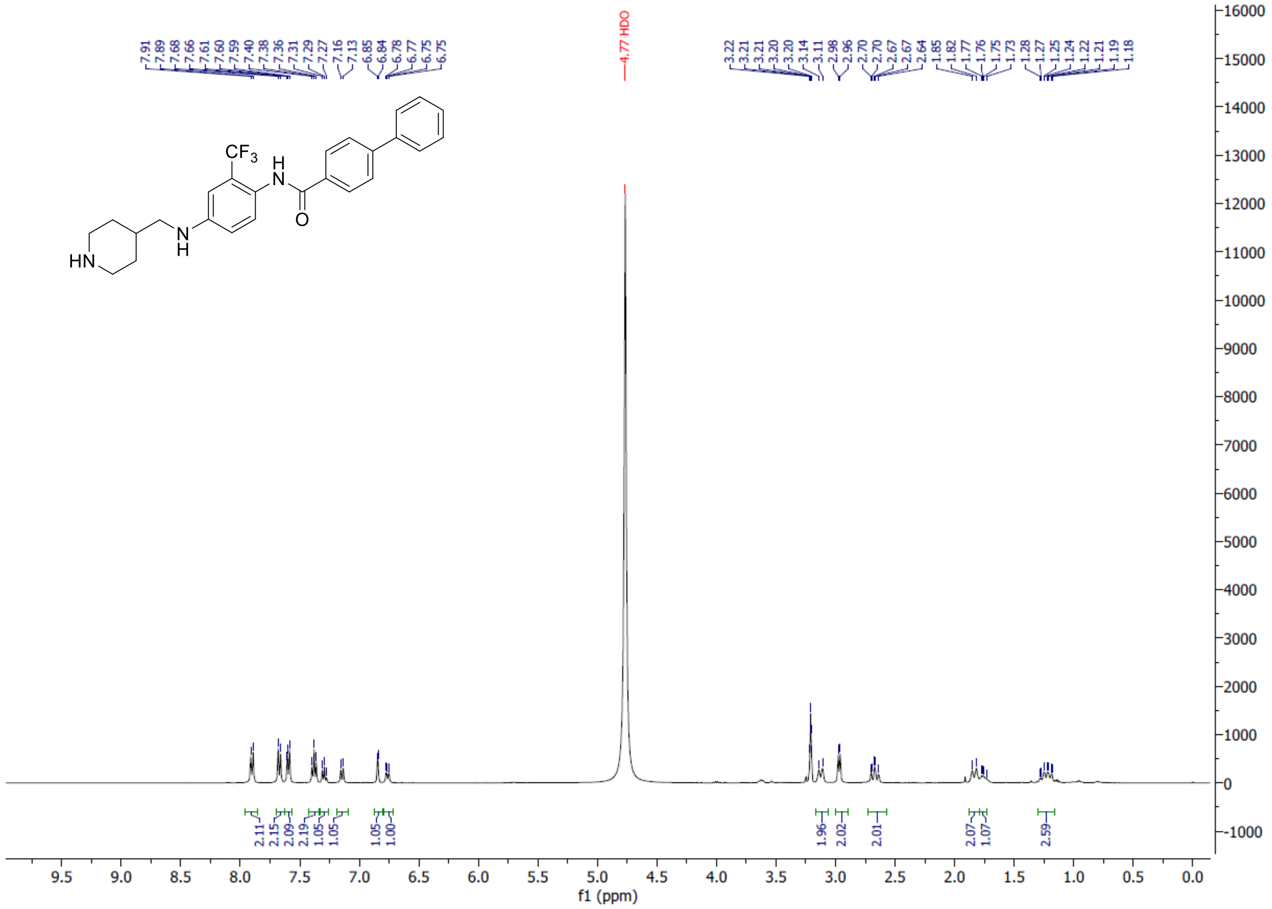

Figure S32: 1H NMR spectrum of compound **28** (MeOD).

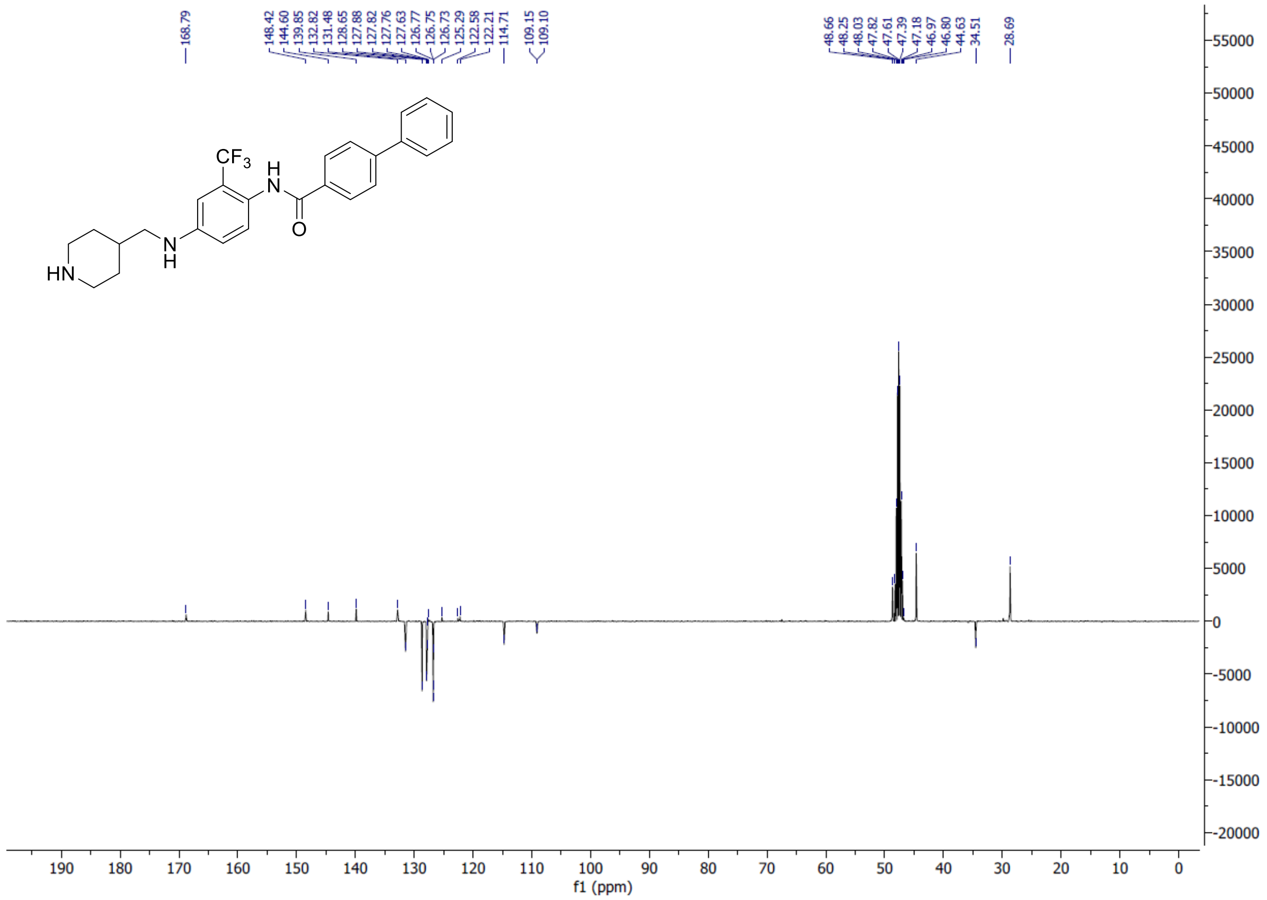

Figure S33: ^{13}C-DEPTq NMR spectrum of compound **28** (MeOD).

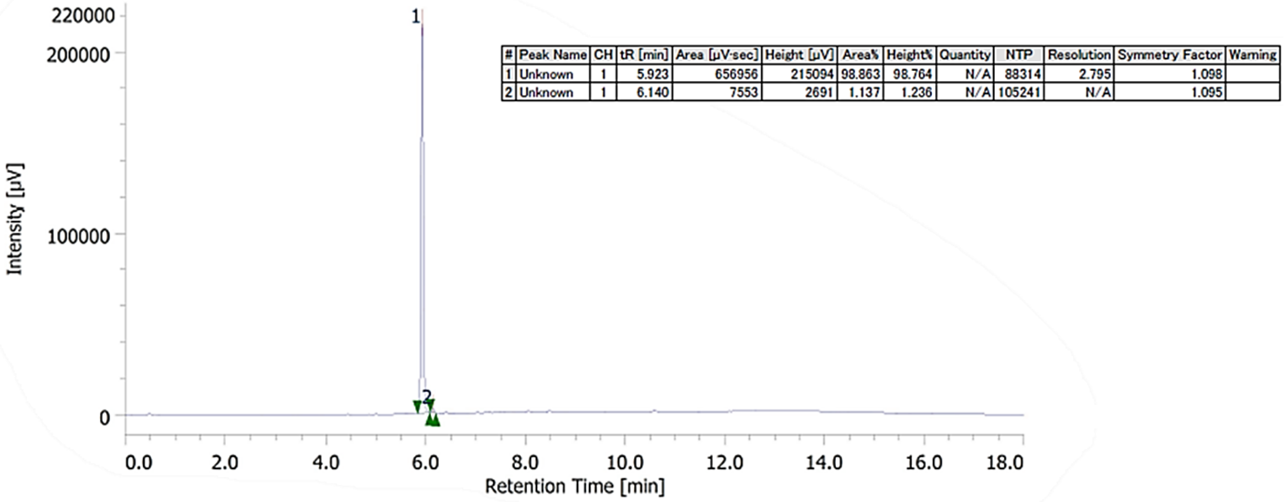

#	Peak Name	CH	tR [min]	Area [µV·sec]	Height [µV]	Area%	Height%	Quantity	NTP	Resolution	Symmetry Factor	Warning
1	Unknown	1	5.923	656956	215094	98.863	98.764	N/A	88314	2.795	1.098	
2	Unknown	1	6.140	7553	2691	1.137	1.236	N/A	105241	N/A	1.095	

Figure S34: HPLC trace of compound **28**.

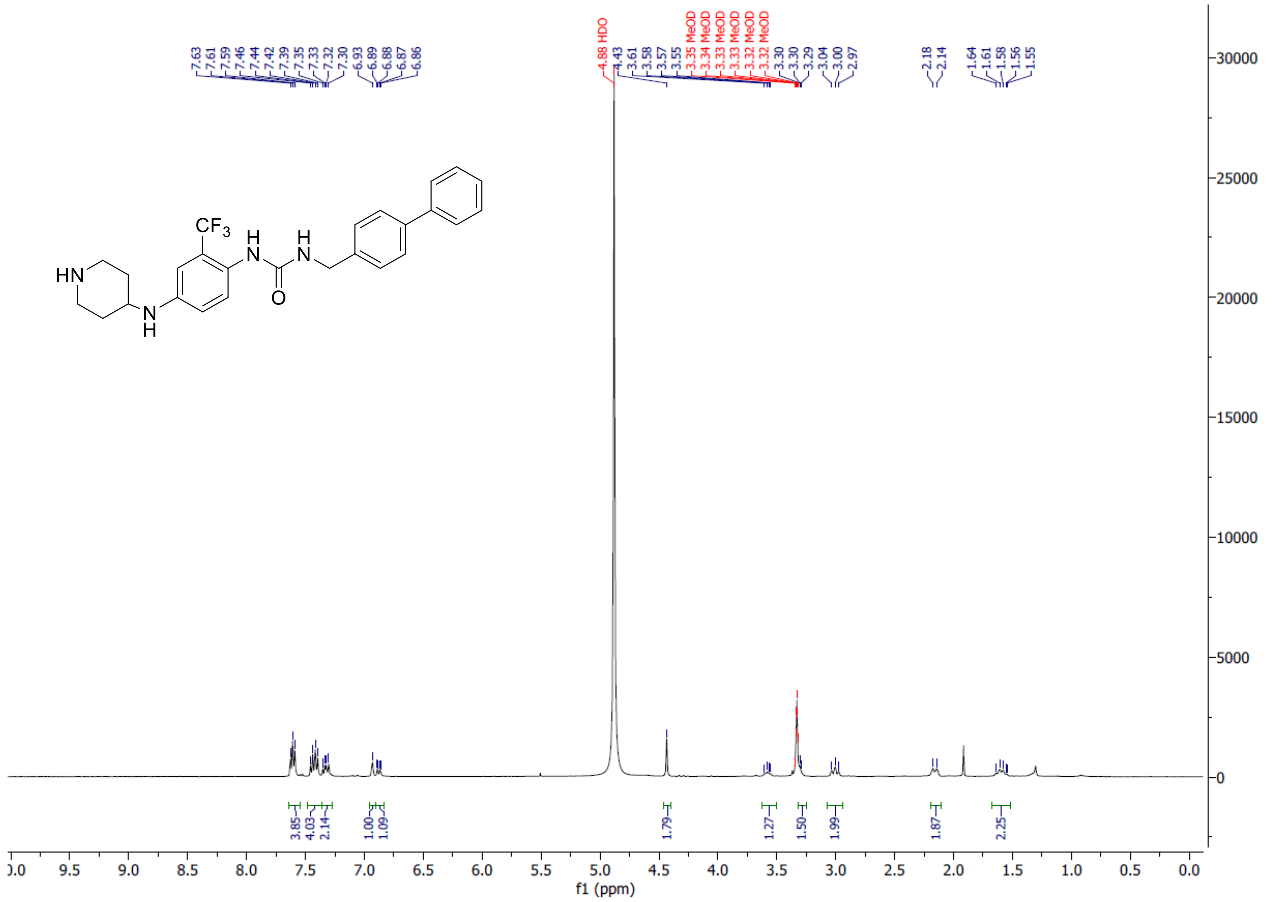

Figure S35: 1H NMR spectrum of compound **29** (MeOD).

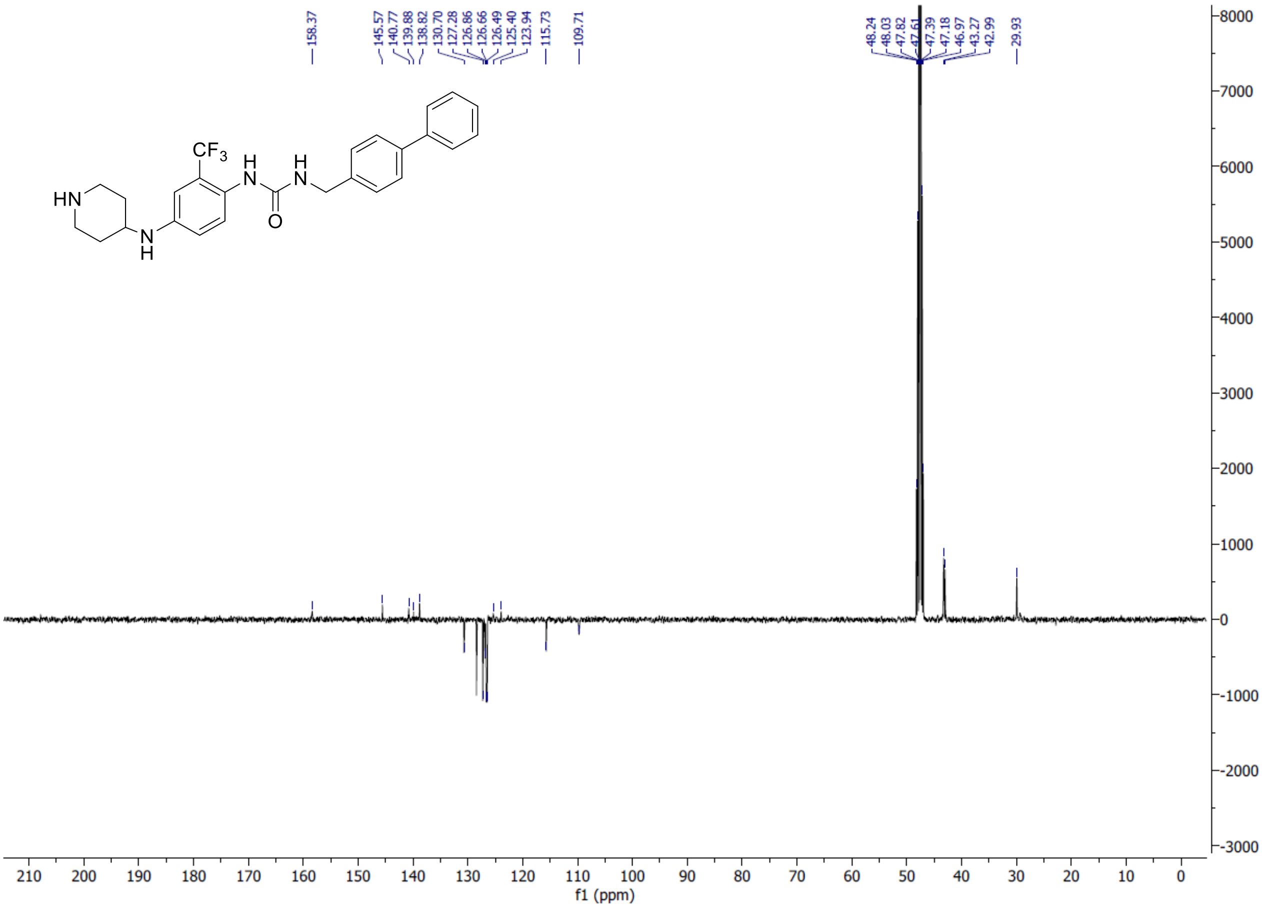

Figure S36: ^{13}C-DEPTq NMR NMR spectrum of compound **29** (MeOD).

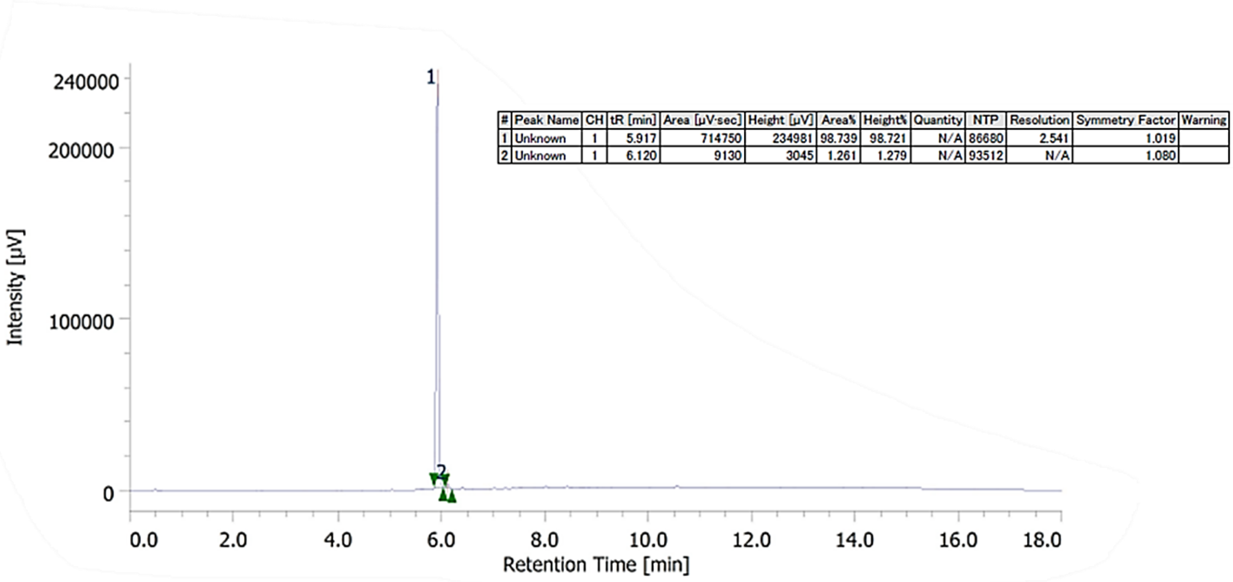

#	Peak Name	CH	tR [min]	Area [µV·sec]	Height [µV]	Area%	Height%	Quantity	NTP	Resolution	Symmetry Factor	Warning
1	Unknown	1	5.917	714750	234981	98.739	98.721	N/A	86680	2.541	1.019	
2	Unknown	1	6.120	9130	3045	1.261	1.279	N/A	93512	N/A	1.080	

Figure S37: HPLC trace of compound **29**.

Kinasic domain – Ponatinib

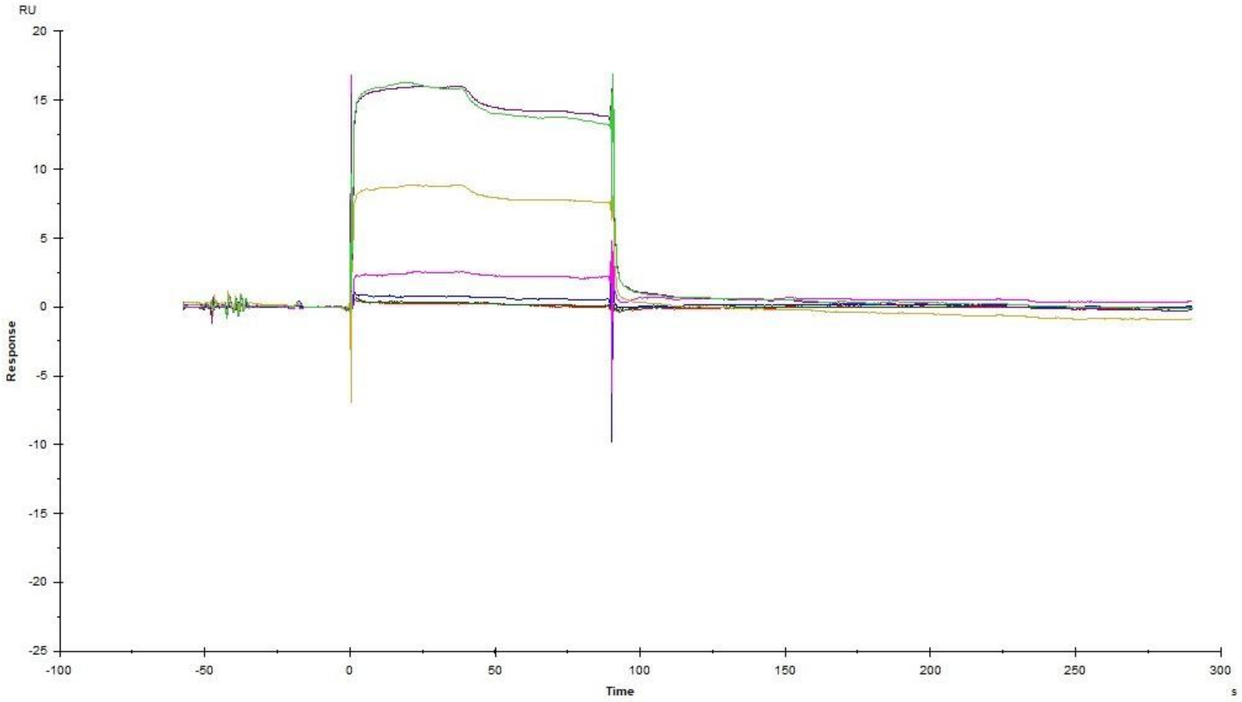

Kinasic domain – 17

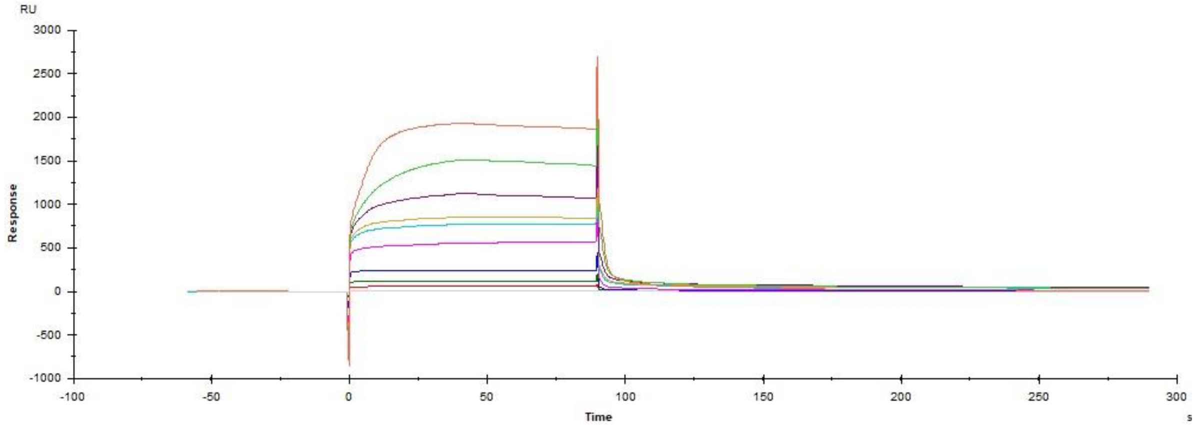

Kinasic domain – 27

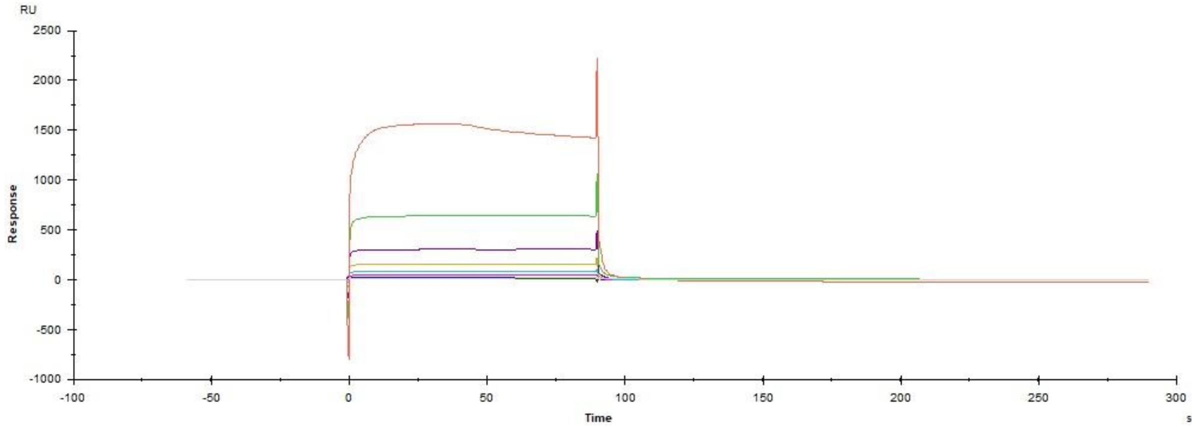

Kinasic domain – 29

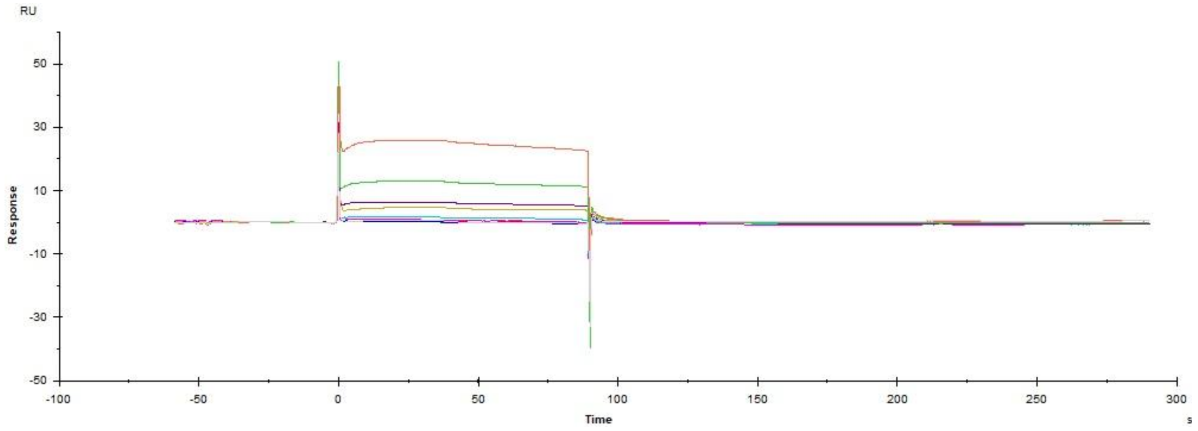

Kinasic domain – 18

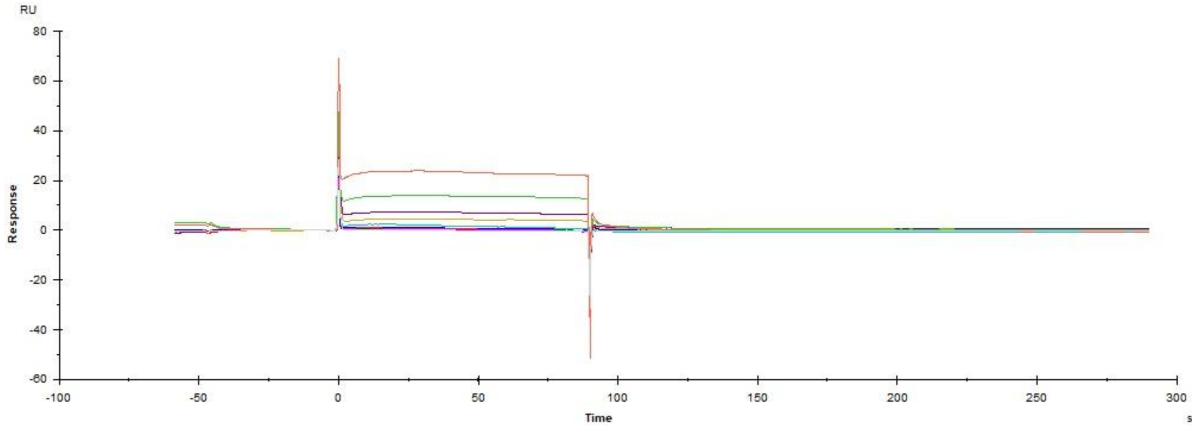

Kinasic domain – 19

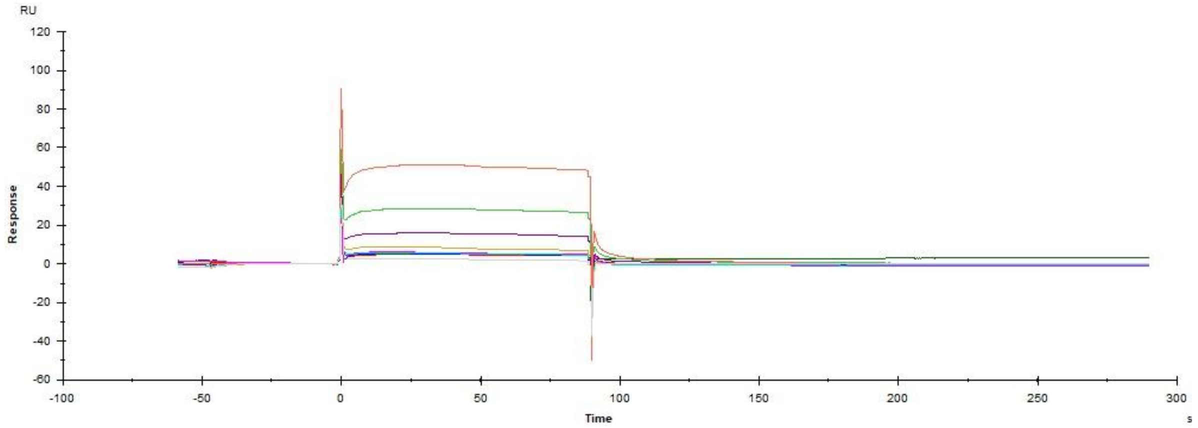

Figure S38. SPR assay raw data. Legend: Sensorgrams corresponding to increasing concentrations of the compound are color-coded as follows: 0 μM – gray, 0.39 μM – red, 0.78 μM – dark green, 1.56 μM – blue, 3.12 μM – fuchsia, 6.25 μM – light blue, 12.5 μM – yellow, 25 μM – purple, 50 μM – light green, and 100 μM – orange.

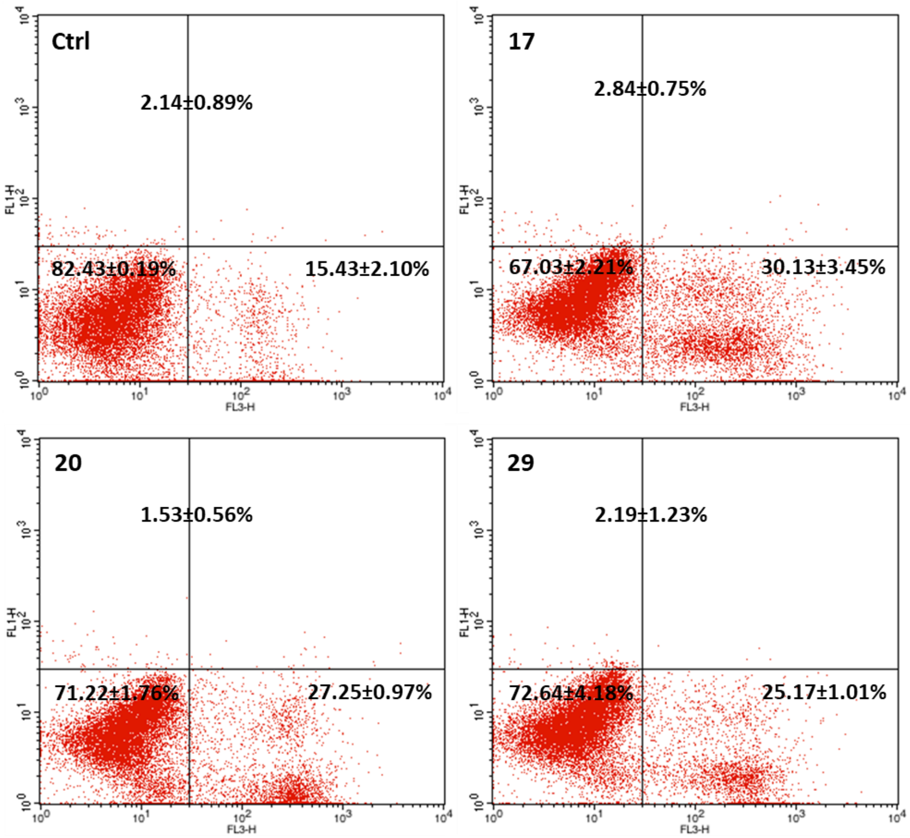

Figure S39. Cell death-promoting activity of compounds 17, 20 and 29 (20 μM) on SH-SY5Y cells treated for 72 h. Representative flow cytometry plots using Annexin V-FITC/PI staining with related quantitative analysis. Data are expressed as a percentage of live, apoptotic and necrotic cells.

WB1: ROR1(17)/α-TUBULIN

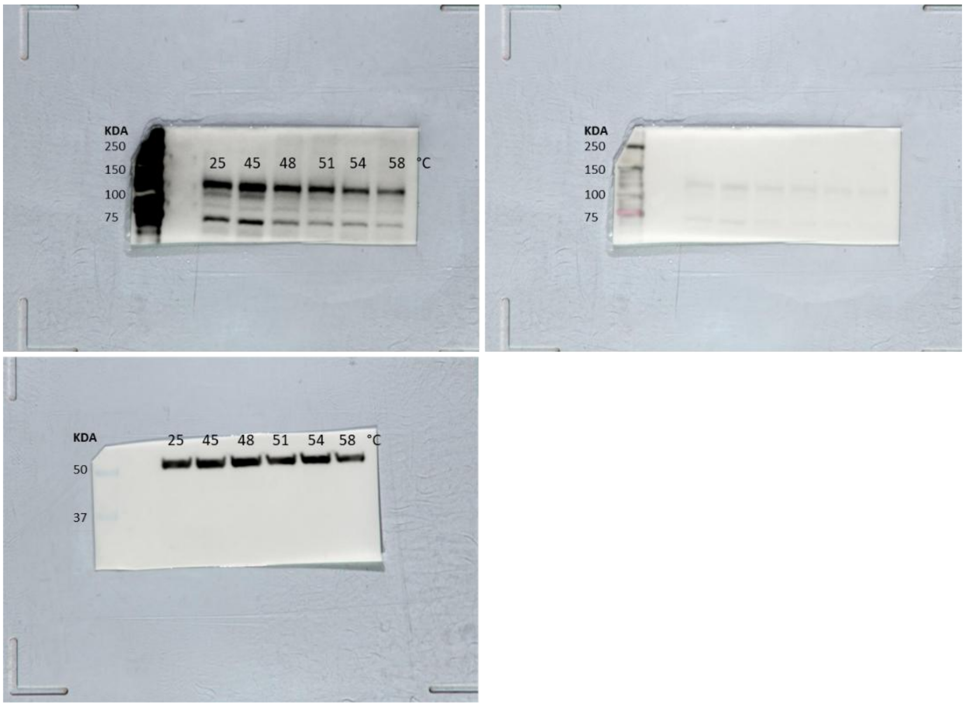

WB2: ROR1(Ctrl)/α-TUBULIN

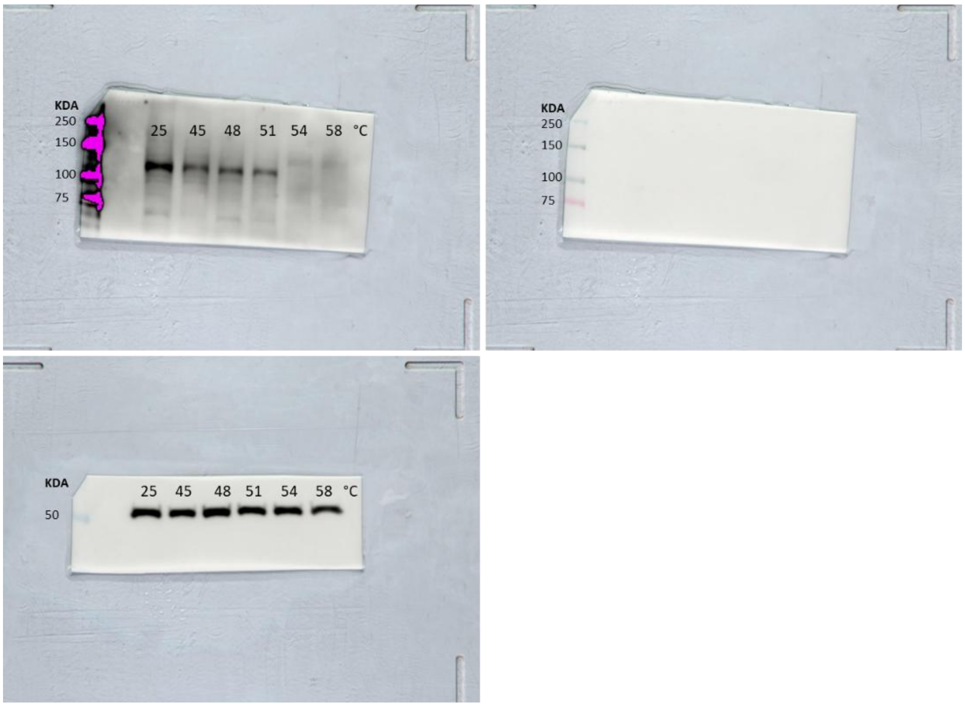

WB3: ROR1(Ponatinib)/α-TUBULIN

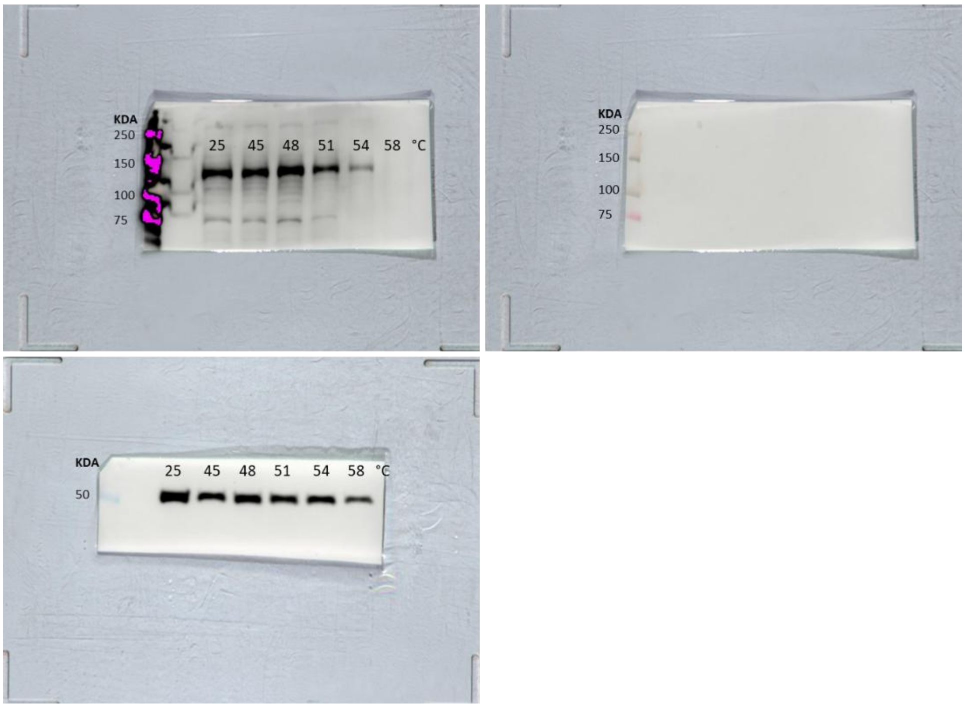

Figure S40. CETSA western blots raw data.

WB1: AKT/α-TUBULIN

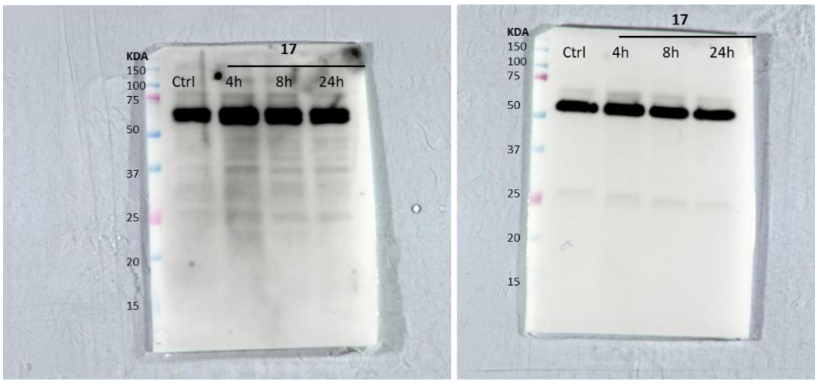

WB2: CASPASE-3/α-TUBULIN

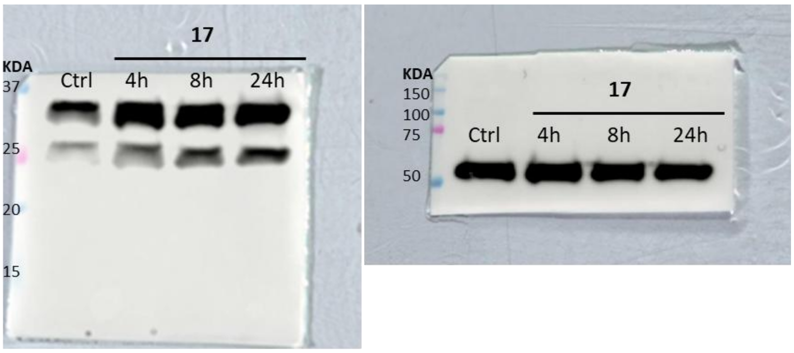

WB3: ROR1/α-TUBULIN

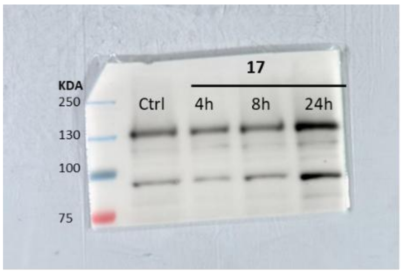

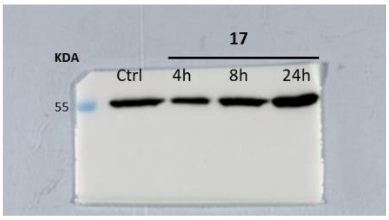

WB4: P-ROR1/α-TUBULIN

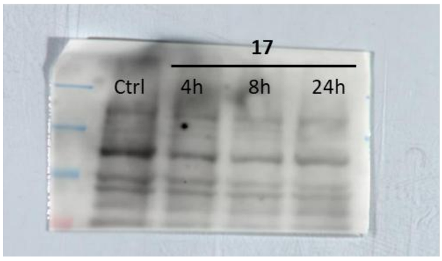

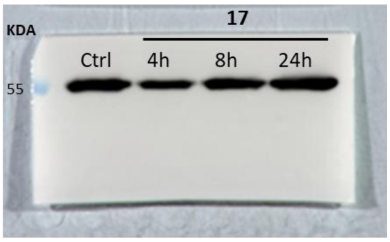

WB5: P-AKT/α-TUBULIN

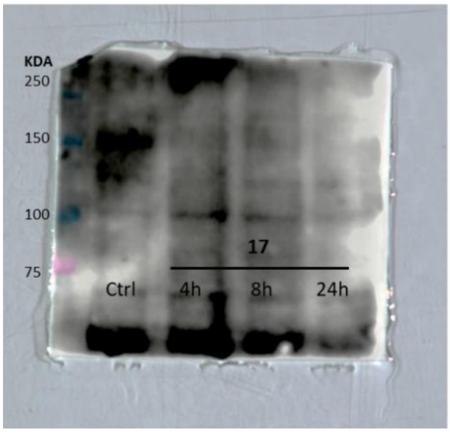

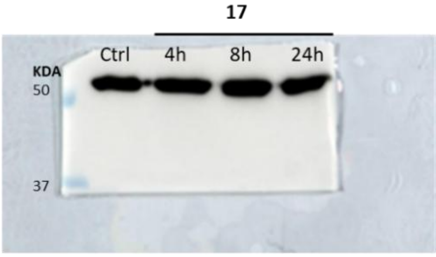

WB6: PARP/ c-PARP /α-TUBULIN

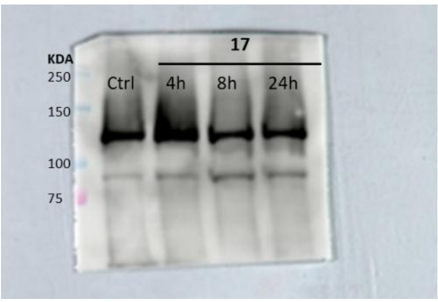

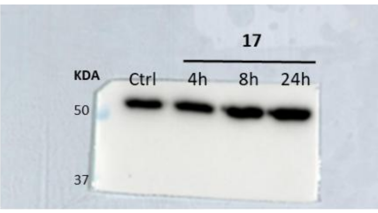

Figure S41. Western blot raw data.

Experimental Section: Chemistry

4-(Isocyanatomethyl)-1,1'-biphenyl

A solution of 4-phenylbenzylamine (1.0 g, 5.46 mmol), triethylamine (663.0 mg, 6.55 mmol) and triphosgene (648.0 mg, 2.2 mmol) in DCM (20 mL) was refluxed for 12 h. Then the mixture was washed with water (2 x 20mL), extracted with DCM, dried, filtered and concentrated under reduced pressure. 4-(Isocyanatomethyl)-1,1'-biphenyl (1.12 g, 98%) was used in the next reaction step without further purification. ^{1}H NMR (400 MHz, $CDCl_3$) δ: 4.55 (s, 2H, C*H2*); 7.38-7.49 (m, 5H, aryl); 7.60-7.64 (m, 4H, aryl). HR-MS m/z not found.

Warning Note: Some precautions must be used for the handling of triphosgene, including vinyl gloves and nitrile gloves and eye protection; further, flame resistant laboratory coats with cuffs must be worn; furthermore, triphosgene should never be exposed to open air outside of a chemical fume hood or glove box. Extreme care should be taken when handling; always work under a chemical fume hood. When working with triphosgene, use explosion-proof equipment. Keep away from sources of ignition. Decomposition of triphosgene solid creates hazardous gases over time during heating or in contact with moisture. Take measures to prevent the buildup heat and moisture.

1-([1,1'-biphenyl]-4-ylmethyl)-3-(4-nitro-2-(trifluoromethyl)phenyl)urea (1): Intermediate 1 (647.0 mg, 39%) was synthesized from 4-nitro-2-(trifluoromethyl)aniline (824.5 mg, 4 mmol) and 4-(isocyanatomethyl)-1,1'-biphenyl (1.0 g, 4.8 mmol) as a brownish powder using the general procedure A. FC in *n*-hexane/ethyl acetate 4/1, R*f*: 0.41.^{1}H NMR (400 MHz, CD_3OD) δ: 4.49 (s, 2H, C*H2*); 7.42-7.45 (m, 5H, aryl); 7.61-7.63 (m, 4H, aryl); 8.15 (d, 1H, aryl, *J*= 6.6 Hz); 8.35 (d, 1H, aryl, *J*= 7.3 Hz); 8.45 (s, 1H, aryl). HR-MS m/z calcd for $C_{21}H_{16}F_3N_3O_3$ $[(M + H)]^+$: 416.1217; found 416.1233.

1-(4-nitrophenyl)-3-(4-(trifluoromethyl)benzyl)urea (2) : Obtained from 4-nitroaniline (552.5 mg, 4 mmol) and 4-(trifluoromethyl)phenyl isocyanate (1.12 g, 6 mmol) as a yellowish powder in 42% of yield (570 mg) applying the general procedure A. FC in n-hexane/ethyl acetate 4/1, R*f*: 0.39. ^{1}H NMR (400 MHz, CD_3OD) δ: 4.37 (s, 2H, C*H2*); 6.61 (d, 1H, aryl, *J*= 6.8 Hz); 6.80 (s, 1H, aryl); 7.20 (d, 1H, aryl, *J*= 8.3 Hz); 7.41 (d, 2H, aryl, *J*= 7.9 Hz); 7.53 (d, 1H, aryl, *J*= 8.1 Hz). HR-MS m/z calcd for $C_{24}H_{21}F_3N_4O$ $[(M + H)]^+$: 439.1740; found 439.1768.

1-([1,1'-biphenyl]-4-ylmethyl)-3-(4-nitrophenyl)urea (3): Intermediate 3 was synthesized from 4-nitroaniline (552.5 mg, 4 mmol) and 4-(isocyanatomethyl)-1,1'-biphenyl (1.25 g, 6 mmol) as a brownish powder in 41% of yield (569 mg) using the general procedure A. FC in n-hexane/ethyl acetate 4/1, R*f*: 0.42. ^{1}H NMR (400 MHz, $(CD_3)_2SO$) δ: 4.37 (s, 2H, C*H2*); 6.99 (bs, 1H, N*H*); 7.34-7.48 (m, 5H, aryl); 7.64-7.67 (m, 6H, aryl); 8.15 (d, 2H, aryl, *J*= 9.2 Hz); 9.40 (s, 1H, N*H*). HR-MS m/z calcd for $C_{20}H_{17}N_3O_3$ $[(M + H)]^+$: 348.1343; found 348.1368.

1-([1,1'-biphenyl]-4-ylmethyl)-3-(2-methoxy-4-nitrophenyl)urea (4): Intermediate 4 (935 mg, 62%) was synthesized from 2-methoxy-4-nitroaniline (672.0 mg, 4 mmol) and 4-(isocyanatomethyl)-1,1'-biphenyl (1.25 g, 6 mmol) as a yellowish powder using the general procedure A. FC in n-hexane/ethyl acetate 3/1, R*f*: 0.43. ^{1}H NMR (400 MHz, $CDCl_3$) δ: 3.93 (s, 3H, C*H3*); 4.53 (d, 2H, C*H2*, *J*= 5.6 Hz); 5.31 (bs, 1H, N*H*); 7.31 (bs, 1H, N*H*); 7.36-7.48 (m, 5H, aryl); 7.58-7.60 (m, 4H, aryl); 7.71 (d, 1H, aryl, *J*= 2.6 Hz); 7.92 (dd, 1H, aryl, *J'*= 2.3 Hz, *J''*= 9.0 Hz); 8.44 (d, 1H, aryl, *J*= 9.0 Hz). HR-MS m/z calcd for $C_{21}H_{19}N_3O_4$ $[(M + H)]^+$: 378.1448; found 378.1482.

1-([1,1'-biphenyl]-4-ylmethyl)-3-(4-amino-2-(trifluoromethyl)phenyl)urea (5) : Synthesized from 1 as an off-white powder in 83% yield (498.0 mg) using the general procedure B. FC in n-hexane/ethyl acetate 1/1, R*f*: 0.44. ^{1}H NMR (400 MHz, CD_3OD) δ: 4.43 (s, 2H, C*H2*); 6.88 (d, 1H, aryl, *J*= 8.5 Hz); 6.98 (d, 1H, aryl, *J*= 2.6 Hz); 7.27 (d, 1H, aryl, *J*= 8.6 Hz); 7.34 (d, 1H, aryl, *J*= 7.4 Hz); 7.39-7.46 (m, 4H, aryl); 7.61 (t, 4H, aryl, *J*= 8.0 Hz). HR-MS m/z calcd for $C_{21}H_{18}F_3N_3O$ $[(M + H)]^+$: 386.1475; found 386.1499.

1-(4-aminophenyl)-3-(4-(trifluoromethyl)benzyl)urea (6): Obtained from 2 in 92% of yield (478.0 mg) applying the general procedure B as a yellowish powder. FC in n-hexane/ethyl acetate 3/2, R*f*: 0.44. ^{1}H NMR (400 MHz, CD_3OD) δ: 4.37 (s, 2H, C*H2*); 6.61 (d, 1H, aryl, *J*= 6.8 Hz); 6.80 (s, 1H, aryl); 7.20 (d, 1H, aryl, *J*= 8.3 Hz); 7.41 (d, 2H, aryl, *J*= 7.9 Hz); 7.53 (d, 1H, aryl, *J*= 8.1 Hz). HR-MS m/z calcd for $C_{15}H_{14}F_3N_3O$ $[(M + H)]^+$: 310.1162; found 310.1177.

1-([1,1'-biphenyl]-4-ylmethyl)-3-(4-aminophenyl)urea (7): Intermediate 7 (389 mg, 75%) was synthesized from 3 as an off-white powder using the general procedure B. FC in n-hexane/ethyl acetate 1/1, R*f*: 0.44. ^{1}H NMR (400 MHz, CD_3OD δ: 4.43 (s, 2H, C*H₂*); 6.71 (d, 2H, aryl, *J*= 8.6 Hz); 7.11 (d, 2H, aryl, *J*= 8.6 Hz); 7.33 (t, 1H, aryl, *J*= 7.3 Hz); 7.40-7.45 (m, 4H, aryl); 7.61 (t, 4H, aryl, *J*= 6.2 Hz). HR-MS m/z calcd for $C_{20}H_{19}N_3O$ $[(M + H)]^+$: 318.1601; found 318.1633.

1-([1,1'-biphenyl]-4-ylmethyl)-3-(4-amino-2-methoxyphenyl)urea (8): Synthesized from 4 as an off-white powder in 79% of yield (533.5 mg) using the general procedure B. FC in n-hexane/ethyl acetate 1/1, R*f*: 0.44.^{1}H NMR (400 MHz, $CDCl_3$) δ: 3.79 (s, 3H, CH_3); 4.50 (d, 2H, CH_2, *J*= 5.8 Hz); 4.93 (bs, 1H, NH); 6.14 (s, 1H, C*H*); 6.27-6.30 (m, 2H, aryl); 7.34-7.47 (m, 5H, aryl); 7.58 (t, 1H, aryl, *J*= 8.6 Hz). HR-MS m/z calcd for $C_{21}H_{21}N_3O_2$ $[(M + H)]^+$: 348.1707; found 348.1742.

***tert*-butyl (3-((4-(3-([1,1'-biphenyl]-4-ylmethyl)ureido)-3-(trifluoromethyl)phenyl)amino)propyl)carbamate (9):** Intermediate 9 (357.7 mg, 51%) was synthesized from 5 (498.0 mg, 1.29 mmol) and 1-Boc-piperidine-4-carboxaldehyde (330.0 mg, 1.55 mmol) as a whitish oil using the general procedure C. FC in dichloromethane/ethyl acetate 4/1, R*f*: 0.44. ^{1}H NMR (400 MHz, $CDCl_3$) δ: 1.46 (s, 9H, CH_3); 1.87 (t, 2H, CH_2, *J*= 7.8 Hz); 3.49 (dd, 2H, CH_2, *J'*= 7.0 Hz, *J''*= 14.0 Hz); 4.14 (dd, 2H, CH_2, *J'*= 7.2 Hz, *J''*= 14.3 Hz); 6.85 (d, 1H, aryl, *J*= 7.4 Hz); 7.22 (s, 1H, aryl); 7.48-7.53 (m, 4H, aryl); 7.66-7.69 (m, 4H, aryl); 7.75 (d, 2H, aryl, *J*= 8.0 Hz). HR-MS m/z calcd for $C_{29}H_{33}F_3N_4O_3$ $[(M + H)]^+$: 543.2578; found 543.2565.

1-([1,1'-biphenyl]-4-ylmethyl)-3-(4-((2-cyanoethyl)amino)-2-(trifluoromethyl)phenyl)urea (10): Final derivative 10 (244.0 mg, 43%) was synthesized from 5 and 3-bromopropionitrile (346.6 mg, 2.6 mmol) as an off-white oil using the general procedure E. FC in dichloromethane/ethyl acetate 4.8/0.2, R*f*: 0.41. ^{1}H NMR (400 MHz, CD_3OD) δ: 2.57-2.64 (m, 1H, C*H*); 2.72-2.80 (m, 1H, C*H*); 3.11-3.18 (m, 1H, C*H*); 4.15-4.27 (m, 3H, C*H* and CH_2); 6.81 (dd, 1H, aryl, *J'*= 2.5 Hz; *J''*= 8.5 Hz); 6.95 (d, 1H, aryl, *J*= 2.6 Hz); 7.13 (d, 1H, aryl, *J*= 8.5 Hz); 7.20 (t, 3H, aryl, *J*= 8.3 Hz); 7.31 (t, 2H, aryl, *J*= 7.8 Hz); 7.42 (d, 2H, aryl, *J*= 8.3 Hz); 7.47 (d, 2H, aryl, *J*= 7.2 Hz). ^{13}C-DEPTq NMR (100 MHz, CD_3OD) δ: 16.1, 43.5, 46.0, 112.76, 112.8, 117.8, 118.3, 125.5, 126.4, 126.5, 126.8, 127.1, 128.4, 133.1, 139.0, 139.6, 140.9, 149.6, 158.4, 165.2. HR-MS m/z calcd for $C_{24}H_{21}F_3N_4O$ $[(M + H)]^+$: 439.1740; found 439.1788.

***tert*-butyl (3-((4-(3-([1,1'-biphenyl]-4-ylmethyl)ureido)-3-(trifluoromethyl)phenyl)amino)propyl)carbamate (11):** Intermediate 11 (517.0 mg, 40%) was synthesized from 5 and 3-(Boc-amino)propyl bromide (831.0 mg, 2.6 mmol) as a whitish oil using the general procedure E. FC in dichloromethane/ethyl acetate 4/1, R*f*: 0.45. ^{1}H NMR (400 MHz, CD_3OD) δ: 1.46 (s, 9H, CH_3); 2.66 (t, 2H, CH_2, *J*= 6.6 Hz); 3.46 (t, 2H, CH_2, *J*= 6.6 Hz); 4.42 (s, 2H, CH_2); 3.11-3.18 (m, 2H, CH_2); 6.88 (d, 2H, aryl, *J*= 8.7 Hz); 6.93 (d, 1H, aryl, *J*= 2.6 Hz); 7.32-7.36 (m, 2H, aryl); 7.43 (dd, 4H, aryl, *J'*= 8.4 Hz, *J''*= 16.7 Hz); 7.61 (t, 4H, aryl, *J*= 7.7 Hz). HR-MS m/z calcd for $C_{24}H_{25}F_3N_4O$ $[(M + H)]^+$: 443.2053; found 443.2068.

1-([1,1'-biphenyl]-4-ylmethyl)-3-(4-((4-nitrobenzyl)amino)-2-(trifluoromethyl)phenyl)urea (12): Compound 12 (672 mg, 53%) was synthesized from 5 and 4-nitrobenzaldehyde (234.5 mg, 1.55 mmol) as a yellowish oil using the general procedure C. FC in n-hexane/ethyl acetate 2/1, R*f*: 0.45. ^{1}H NMR (400 MHz, CD_3OD) δ: 4.41 (s, 2H, CH_2); 4.51 (s, 2H, CH_2); 6.77 (d, 1H, aryl, *J*= 6.0 Hz); 6.88 (s, 1H, aryl); 7.27 (d, 1H, aryl, *J*= 8.7 Hz); 7.37-7.44 (m, 4H, aryl); 7.57-7.62 (m, 6H, aryl); 8.20 (d, 1H, aryl, *J*= 8.8 Hz). HR-MS m/z calcd for $C_{28}H_{23}F_3N_4O_3$ $[(M + H)]^+$: 520.1722; found 520.1748.

1-([1,1'-biphenyl]-4-ylmethyl)-3-(4-((pyridin-4-ylmethyl)amino)-2-(trifluoromethyl)phenyl)urea (13): Final derivative 13 (357 mg, 58%) was synthesized from 5 and 4-pyridinecarboxaldehyde (166 mg, 1.55 mmol) as an off-white oil using the general procedure C. FC in n-hexane/ethyl acetate 3/1, R*f*: 0.41. ^{1}H NMR (400 MHz, CD_3OD) δ: 4.41 (s, 2H, CH_2); 4.45 (s, 2H, CH_2); 6.76 (dd, 1H, aryl, *J'*= 2.5 Hz; *J''*= 8.7 Hz); 6.86 (d, 2H, aryl, *J*= 2.6 Hz); 7.28 (d, 1H, aryl, *J*= 8.7 Hz); 7.33 (d, 1H, aryl, *J*= 7.4 Hz); 7.38-7.45 (m, 6H, aryl); 7.60 (t, 4H, aryl, *J*= 8.3 Hz); 7.46 (d, 2H, aryl, *J*= 5.9 Hz). ^{13}C-DEPTq NMR (100 MHz, CD_3OD) δ: 43.0, 45.7, 109.2, 109.3, 115.4, 122.4, 124.3, 125.3, 126.2, 126.5, 126.7, 126.8, 127.3, 128.4, 130.6, 138.8, 139.9, 140.8, 146.3, 148.7, 150.8, 158.3. HR-MS m/z calcd for $C_{27}H_{23}F_3N_4O$ $[(M + H)]^+$: 477.1897; found 477.1888.

1-(4-((2-cyanoethyl)amino)phenyl)-3-(4-(trifluoromethyl)benzyl)urea (14): Obtained from 6 and 3-bromopropionitrile (346.5 mg, 2.6 mmol) as a whitish oil in 48% of yield (268.8 mg) using the general procedure E. FC in n-hexane/ethyl acetate 3/1, R*f*: 0.44. ^{1}H NMR (400 MHz, CD_3OD) δ: 2.70 (t, 2H, CH_2, *J*= 6.6 Hz); 3.44

(t, 2H, C*H*$_2$, *J*= 6.6 Hz); 4.46 (s, 2H, C*H*$_2$); 3.11-3.18 (m, 1H, C*H*); 6.65 (d, 2H, aryl, *J*= 8.8 Hz); 7.14 (d, 1H, aryl, *J*= 8.9 Hz); 7.52 (d, 2H, aryl, *J*= 8.0 Hz); 7.64 (d, 2H, aryl, *J*= 8.1 Hz). ^{13}C-DEPTq NMR (100 MHz, CD_3OD) δ: 16.9, 39.8, 42.7, 113.1, 118.6, 122.9, 124.9, 127.2, 129.2, 144.1, 144.7, 157.8, 177.1. HR-MS m/z calcd for $C_{24}H_{21}F_3N_4O$ [(M + H)]$^+$: 439.1740; found 439.1784.

***tert*-butyl 4-(((4-(3-([1,1'-biphenyl]-4-ylmethyl)ureido)phenyl)amino)methyl)piperidine-1-carboxylate (15):** Intermediate 15 (334.3 mg 53%) was synthesized from 7 and 1-Boc-piperidine-4-carboxaldehyde (330.0 mg, 1.55 mmol) as an off-white oil in yield using the general procedure C. FC in dichloromethane/ethyl acetate 4.5/0.5, R*f*: 0.44. ^{1}H NMR (400 MHz, CD_3OD δ: 1.09-1.20 (m, 3H, C*H* and C*H*$_2$); 1.47 (s, 9H, 3C*H*$_3$); 1.80 (d, 2H, C*H*$_2$, *J*= 10.3 Hz); 2.76 (bs, 2H, C*H*$_2$); 2.98 (d, 2H, C*H*$_2$, *J*= 6.2 Hz); 4.10 (d, 2H, C*H*$_2$, *J*= 12.7 Hz); 4.42 (s, 2H, C*H*$_2$); 6.62 (d, 2H, aryl, *J*= 8.7 Hz); 7.10 (d, 2H, aryl, *J*= 8.7 Hz); 7.33 (t, 1H, aryl, *J*= 7.3 Hz); 7.40-7.45 (m, 4H, aryl); 7.60 (t, 4H, aryl, *J*= 6.6 Hz). HR-MS m/z calcd for $C_{31}H_{38}N_4O_3$ [(M + H)]$^+$: 515.3017; found 515.3064.

***tert*-butyl 4-(((4-(3-([1,1'-biphenyl]-4-ylmethyl)ureido)-3-methoxyphenyl)amino)methyl)piperidine-1-carboxylate (16) :** Synthesized from 8 and 1-Boc-piperidine-4-carboxaldehyde (330.0 mg, 1.55 mmol) an off-white oil in 57% of yield (476.8 mg) using the general procedure C. FC in dichloromethane/ethyl acetate 4.5/0.5, R*f*: 0.38. ^{1}H NMR (400 MHz, CD_3OD) δ: 1.39-1.48 (m, 2H, C*H*$_2$); 1.53 (s, 9H, C*H*$_3$); 1.92-1.98 (m, 1H, C*H*); 2.03 (d, 2H, C*H*$_2$, *J*= 14.0 Hz); 2.94 (t, 2H, C*H*$_2$, *J*= 12.7 Hz); 3.03 (bs, 2H, C*H*$_2$); 3.40 (d, 2H, C*H*$_2$, *J*= 12.6 Hz); 3.80 (s, 3H, C*H*$_3$); 4.40 (s, 2H, C*H*$_2$); 6.37 (s, 1H, aryl); 7.31 (t, 1H, aryl, *J*= 7.2 Hz); 7.40-7.44 (m, 4H, aryl); 7.50 (s, 1H, aryl); 7.61 (t, 4H, aryl, *J*= 8.0 Hz). HR-MS m/z calcd for $C_{32}H_{40}N_4O_4$ [(M + H)]$^+$: 545.3122; found 545.3155.

1-([1,1'-biphenyl]-4-ylmethyl)-3-(4-((piperidin-4-ylmethyl)amino)-2-(trifluoromethyl)phenyl)urea (17): Final derivative 17 (242.0 mg,83%) was synthesized from 9 as an off-white powder using the general procedure D. Precipitated from methanol/diethyl ether. ^{1}H NMR (400 MHz, CD_3OD) δ: 1.39-1.51 (m, 2H, C*H*$_2$); 1.89-1.97 (m, 1H, C*H*); 2.05 (d, 1H, C*H*$_2$, *J*= 13.5 Hz); 2.94-3.01 (m, 2H, C*H*$_2$); 3.09 (d, 1H, C*H*$_2$, *J*= 6.8 Hz); 3.41 (d, 1H, C*H*$_2$, *J*= 12.7 Hz); 4.44 (s, 2H, C*H*$_2$); 6.82-6.85 (m, 1H, aryl); 6.88 (d, 1H, aryl, *J*= 2.7 Hz); 7.29-7.35 (m, 2H, aryl); 7.39-7.46 (m, 4H, aryl); 7.59-7.63 (m, 4H, aryl). ^{13}C-DEPTq NMR (100 MHz, CD_3OD) δ: 26.7, 33.4, 43.0, 43.7, 48.1, 108.8, 108.9, 115.0, 123.6, 125.4, 126.5, 126.7, 126.9, 127.3, 128.4, 130.7, 138.8, 139.9, 147.0, 158.4. HR-MS m/z calcd for $C_{27}H_{29}F_3N_4O$ [(M + H)]$^+$: 483.2366; found 483.2366.

1-([1,1'-biphenyl]-4-ylmethyl)-3-(4-((piperidin-4-ylmethyl)amino)phenyl)urea (18): Final derivative 18 (380.0 mg, 87%) was obtained applying the general procedure D from 15 as an off-white powder. Precipitated from methanol/diethyl ether. ^{1}H NMR (400 MHz, CD_3OD) δ: 1.28-1.37 (m, 2H, C*H*$_2$); 1.92 (d, 2H, C*H*$_2$, *J*= 2.6 Hz); 2.84 (t, 2H, C*H*$_2$, *J*= 12.8 Hz); 2.92 (d, 2H, C*H*$_2$, *J*= 6.8 Hz); 3.28 (d, 2H, C*H*$_2$, *J*= 12.7 Hz); 4.31 (s, 2H, C*H*$_2$); 6.51 (d, 2H, aryl, *J*= 8.8 Hz); 7.00 (d, 2H, aryl, *J*= 8.8 Hz); 7.21 (t, 1H, aryl, *J*= 7.4 Hz); 7.28-7.33 (m, 4H, aryl); 7.49 (t, 4H, aryl, *J*= 8.0 Hz). ^{13}C-DEPTq NMR (100 MHz, CD_3OD) δ: 26.8, 33.5, 42.9, 43.8, 48.9, 115.4, 118.3, 122.8, 126.5, 126.7, 126.9, 127.3, 128.4, 139.0, 139.9, 140.8, 145.3, 157.9, 161.5. HR-MS m/z calcd for $C_{26}H_{30}N_4O$ [(M + H)]$^+$: 415.2492; found 415.2443.

1-([1,1'-biphenyl]-4-ylmethyl)-3-(2-methoxy-4-((piperidin-4-ylmethyl)amino)phenyl)urea (19): The general procedure D led to the final derivative 19 (330 mg, 85%), that was synthesized from 16 as an off-white powder. Precipitated from methanol/diethyl ether. ^{1}H NMR (400 MHz, CD_3OD) δ: 1.40-1.51 (m, 2H, C*H*$_2$); 1.93-1.99 (m, 1H, C*H*); 2.05 (d, 2H, C*H*$_2$, *J*= 14.1 Hz); 2.96 (t, 2H, C*H*$_2$, *J*= 12.8 Hz); 3.07 (bs, 2H, C*H*$_2$); 3.40 (d, 2H, C*H*$_2$, *J*= 12.7 Hz); 3.83 (s, 3H, C*H*$_3$); 4.42 (s, 2H, C*H*$_2$); 6.40 (s, 1H, aryl); 7.33 (t, 1H, aryl, *J*= 7.4 Hz); 7.39-7.45 (m, 4H, aryl); 7.51 (s, 1H, aryl); 7.60 (t, 4H, aryl, *J*= 8.0 Hz). ^{13}C-DEPTq NMR (100 MHz, CD_3OD) δ: 26.6, 42.9, 43.7, 54.7, 97.4, 118.5, 126.5, 126.7, 126.9, 127.3, 128.4, 138.9, 139.8, 140.8, 158.0. HR-MS m/z calcd for $C_{27}H_{32}N_4O_2$ [(M + H)]$^+$: 445.2598; found 445.2598.

1-([1,1'-biphenyl]-4-ylmethyl)-3-(4-((3-aminopropyl)amino)-2-(trifluoromethyl)phenyl)urea (20): Final derivative 20 (345 mg, 82%) was synthesized from 11 as an off-white powder using the general procedure D. Precipitated from methanol/diethyl ether. ^{1}H NMR (400 MHz, CD_3OD) δ: 1.95-2.02 (m, 2H, C*H*$_2$); 3.07 (t, 2H, C*H*$_2$, *J*= 7.7 Hz); 3.26 (t, 2H, C*H*$_2$, *J*= 6.7 Hz); 4.44 (s, 2H, C*H*$_2$); 6.85 (d, 2H, aryl, *J*= 8.7 Hz); 6.91 (d, 1H, aryl, *J*= 2.6 Hz); 7.34 (t, 2H, aryl, *J*= 8.2 Hz); 7.40-7.46 (m, 4H, aryl); 7.61 (t, 4H, aryl, *J*= 6.4 Hz). ^{13}C-DEPTq NMR (100 MHz, CD_3OD) δ: 26.6, 37.4, 40.1, 43.0, 109.0, 115.4, 122.7, 124.2, 125.4, 126.5, 126.7, 126.9, 127.3, 128.4, 130.6, 138.8, 139.9, 140.8, 146.6, 158.3. HR-MS m/z calcd for $C_{24}H_{25}F_3N_4O$ [(M + H)]$^+$: 443.2053; found 443,2077.

1-([1,1'-biphenyl]-4-ylmethyl)-3-(4-((4-aminobenzyl)amino)-2-(trifluoromethyl)phenyl)urea (21): Compound 21 (487.5 mg, 77%) was synthesized from 12 as a whitish powder using the general procedure B. FC in n-hexane/ethyl acetate 1/3, R*f*: 0.44. ^{1}H NMR (400 MHz, CD_3OD) δ: 4.20 (s, 2H, C*H2*); 4.42 (s, 2H, C*H2*); 6.72 (d, 2H, aryl, *J*= 8.3 Hz); 6.82 (d, 1H, aryl, *J*= 10.9 Hz); 6.88 (d, 1H, aryl, *J*= 2.5 Hz); 7.13 (d, 2H, aryl, *J*= 8.2 Hz); 7.24 (d, 1H, aryl, *J*= 8.7 Hz); 7.33 (t, 1H, aryl, *J*= 8.7 Hz); 7.38-7.45 (m, 4H, aryl); 7.60 (t, 1H, aryl, *J*= 8.4 Hz). ^{13}C-DEPTq NMR (100 MHz, CD_3OD) δ: 43.0, 46.8, 109.1, 109.2, 115.4, 115.6, 123.5, 125.4, 126.5, 126.6, 126.8, 127.3, 128.0, 128.4, 128.7, 138.8, 139.9, 140.8, 146.2, 147.2, 158.4. HR-MS m/z calcd for $C_{28}H_{25}F_3N_4O$ $[(M + H)]^+$: 491.2053; found 491.2063.

***N*-(4-nitro-2-(trifluoromethyl)phenyl)-[1,1'-biphenyl]-4-sulfonamide (22):** Intermediate 22 was synthesized from 4-nitro-2-(trifluoromethyl)aniline (412.0 mg, 2 mmol) and biphenyl-4-sulfonyl chloride. The starting material was dissolved in tetrahydrofuran (10 mL) under magnetic stirring and added with 4 mmol of NaH (160 mg, 60 % dispersion in mineral oil) at room temperature. Afterwards, 3 mmol of biphenyl-4-sulfonyl chloride (758.0 mg) were added and the mixture was stirred at 90°C for 12 h. Then, the reaction was washed with a 2N aqueous solution of HCl, extracted with ethyl acetate, dried, filtered and evaporated under vacuum. The flash chromatography of the residue furnished intermediate 22 in 44% of yield (371.0 mg). FC in n-hexane/ethyl acetate 3/1, R*f*: 0.43. ^{1}H NMR (400 MHz, CD_3OD) δ: 1.39-1.49 (m, 2H, C*H2*); 1.88-1.96 (m, 1H, C*H*); 2.03 (d, 1H, C*H*2, *J*= 13.5 Hz); 2.94-3.00 (m, 2H, C*H2*); 3.05 (d, 1H, C*H*2, *J*= 6.8 Hz); 3.41 (d, 2H, C*H*2, *J*= 12.7 Hz); 6.69 (dd, 1H, aryl, *J'*= 2.7 Hz; *J''*= 8.7 Hz); 6.83 (d, 1H, aryl, *J*= 2.7 Hz); 7.00 (d, 1H, aryl, *J*= 8.8 Hz); 7.40-7.44 (m, 1H, aryl); 7.49 (t, 1H, aryl, *J*= 7.8 Hz); 7.68 (d, 2H, aryl, *J*= 7.3 Hz); 7.80 (dd, 1H, aryl, *J'*= 8.5 Hz; *J''*= 12.0 Hz). HR-MS m/z calcd for $C_{25}H_{26}F_3N_3O_2S$ $[(M + H)]^+$: 490,1771; found 490,1788.

***N*-(4-nitro-2-(trifluoromethyl)phenyl)-[1,1'-biphenyl]-4-carboxamide (23):** 4-Nitro-2-(trifluoromethyl)aniline (412.0 mg, 2 mmol) was dissolved in dichloromethane (10 mL), added with 3 mmol of DBU (365 mg) and 3 mmol of biphenyl-4-carbonyl chloride (649.5 mg), and stirred overnight at 45 °C. Then, the mixture was washed twice with a 2N aqueous solution of HCl, extracted with DCM, dried over Na_2SO_4, filtered and evaporated *in vacuo*. The following purification performed using a flash chromatography system yielded intermediate 23 as a yellowish powder in 47% yield (362.7 mg). FC in n-hexane/ethyl acetate 3/1, R*f*: 0.45. ^{1}H NMR (400 MHz, $CDCl_3$) δ: 7.37 (d, 1H, aryl, *J*= 13.5 Hz); 7.43 (t, 2H, aryl, *J*= 7.7 Hz); 7.58 (d, 2H, aryl, *J*= 7.2 Hz); 7.72 (d, 2H, aryl, *J*= 8.3 Hz); 7.88 (d, 2H, aryl, *J*= 8.4 Hz); 8.42 (d, 1H, aryl, *J*= 6.8 Hz); 8.50-8.53 (m, 2H, aryl); 8.88 (d, 1H, aryl, *J*= 9.2 Hz). HR-MS m/z calcd for $C_{20}H_{13}F_3N_2O_3$ $[(M + H)]^+$: 387.0951; found 387.0964.

***N*-(4-amino-2-(trifluoromethyl)phenyl)-[1,1'-biphenyl]-4-sulfonamide (24):** Synthesized from 22 as a yellowish powder in 79% of yield (272 mg) using the general procedure B. FC in n-hexane/ethyl acetate 1/2, R*f*: 0.43. FC in n-hexane/ethyl acetate 3/1, R*f*: 0.43. ^{1}H NMR (400 MHz, $CDCl_3$) δ: 3.82 (bs, 2H, N*H2*); 6.56 (s, 1H, aryl); 6.77 (d, 1H, aryl, *J*= 2.5 Hz); 6.83-6.86 (m, 1H, aryl); 7.48 (t, 2H, aryl, *J*= 7.6 Hz); 7.58-7.65 (m, 6H, aryl, N*H*); 7.77 (d, 2H, aryl, *J*= 8.3 Hz). HR-MS m/z calcd for $C_{19}H_{15}F_3N_2O_2S$ $[(M + H)]^+$: 393.0879; found 393.0852.

***N*-(4-amino-2-(trifluoromethyl)phenyl)-[1,1'-biphenyl]-4-carboxamide (25):** Obtained from 23 as an off-white powder in 84% yield (281 mg) using the general procedure B. FC in n-hexane/ethyl acetate 1/1, R*f*: 0.44. ^{1}H NMR (400 MHz, $CDCl_3$) δ: 3.87 (bs, 2H, N*H*); 6.92 (d, 1H, aryl, *J*= 8.6 Hz); 6.97 (s, 1H, aryl); 7.44 (d, 1H, aryl, *J*= 7.3 Hz); 7.51 (t, 2H, aryl, *J*= 7.7 Hz); 7.66 (d, 2H, aryl, *J*= 7.3 Hz); 7.75 (d, 2H, aryl, *J*= 8.3 Hz); 7.95-8.00 (m, 4H, aryl). HR-MS m/z calcd for $C_{20}H_{15}F_3N_2O$ $[(M + H)]^+$: 357.1209; found 357.1229.

***N*-(4-((4-aminobenzyl)amino)-2-(trifluoromethyl)phenyl)-[1,1'-biphenyl]-4-sulfonamide (26):** Final derivative 26 (179 mg, 52%) was synthesized from 24 as an off-white powder in using the general procedure B. FC in n-hexane/ethyl acetate 1/1, R*f*: 0.45. ^{1}H NMR (400 MHz, CD_3OD) δ: 4.04 (s, 2H, C*H2*); 6.55 (dd, 1H, aryl, *J'*= 2.7 Hz; *J''*= 8.8 Hz); 6.59 (d, 2H, aryl, *J*= 8.4 Hz); 6.70 (d, 1H, aryl, *J*= 2.7 Hz); 6.83 (d, 1H, aryl, *J*= 8.7 Hz); 6.98 (d, 1H, aryl, *J*= 8.4 Hz); 7.28-7.32 (m, 1H, aryl); 7.38 (t, 2H, aryl, *J*= 7.8 Hz); 7.57 (d, 2H, aryl, *J*= 7.2 Hz); 7.67 (dd, 1H, aryl, *J'*= 8.8 Hz; *J''*= 15.8 Hz). ^{13}C-DEPTq NMR (100 MHz, CD_3OD) δ: 46.6, 109.8, 109.9, 114.8, 115.4, 126.9, 127.0, 127.5, 128.0, 128.1, 128.4, 128.7, 139.3, 130.4, 139.3, 139.7, 145.3, 146.3, 148.2. HR-MS m/z calcd for $C_{26}H_{22}F_3N_3O_2S$ $[(M + H)]^+$: 498.1458; found 498.1489.

***N*-(4-((piperidin-4-ylmethyl)amino)-2-(trifluoromethyl)phenyl)-[1,1'-biphenyl]-4-sulfonamide (27):** Final derivative 27 (118.8 mg, 35%) was synthesized from 24 as an off-white powder using the general procedure D. Precipitated from methanol/diethyl ether. ^{1}H NMR (400 MHz, CD_3OD) δ: 1.39-1.49 (m, 2H, C*H2*); 1.88-1.96 (m, 1H, C*H*); 2.03 (d, 1H, C*H*2, *J*= 13.5 Hz); 2.94-3.00 (m, 2H, C*H2*); 3.05 (d, 1H, C*H*2, *J*= 6.8 Hz); 3.41 (d, 2H,

C*H*$_2$, *J*= 12.7 Hz); 6.69 (dd, 1H, aryl, *J'*= 2.7 Hz; *J''*= 8.7 Hz); 6.83 (d, 1H, aryl, *J*= 2.7 Hz); 7.00 (d, 1H, aryl, *J*= 8.8 Hz); 7.40-7.44 (m, 1H, aryl); 7.49 (t, 1H, aryl, *J*= 7.8 Hz); 7.68 (d, 2H, aryl, *J*= 7.3 Hz); 7.80 (dd, 1H, aryl, *J'*= 8.5 Hz; *J''*= 25.3 Hz). ^{13}C-DEPTq NMR (100 MHz, CD_3OD) δ: 26.6, 33.2, 43.6, 109.6, 109.7, 114.2, 121.0, 122.4, 125.0, 126.9, 127.0, 127.5, 128.1, 128.7, 130.6, 139.2, 139.8, 145.3, 148.1. HR-MS m/z calcd for $C_{25}H_{26}F_3N_3O_2S$ $[(M + H)]^+$: 490,1771; found 490,1742.

***N*-([1,1'-biphenyl]-4-yl)-4-((piperidin-4-ylmethyl)amino)-2-(trifluoromethyl)benzamide (28):** Final derivative 28 (157 mg, 44%) was synthesized from 25 as a whitish powder using the general procedure D. Precipitated from methanol/diethyl ether. ^{1}H NMR (400 MHz, CD_3OD) δ: 1.18-1.28 (m, 2H, C*H$_2$*); 1.73-1.85 (m, 3H, C*H* and C*H$_2$*); 2.67 (t, 2H, C*H*$_2$, *J*= 12.5 Hz); 2.97 (d, 2H, C*H*$_2$, *J*= 6.6 Hz); 3.12 (d, 2H, C*H*$_2$, *J*= 12.5 Hz); 6.76 (d, 1H, aryl, *J*= 6.6 Hz); 6.84 (s, 1H, aryl); 7.15 (d, 1H, aryl, *J*= 8.6 Hz); 7.29 (t, 1H, aryl, *J*= 7.3 Hz); 7.38 (t, 2H, aryl, *J*= 7.8 Hz); 7.59 (d, 2H, aryl, *J*= 7.3 Hz); 7.67 (d, 2H, aryl, *J*= 8.3 Hz); 7.90 (d, 2H, aryl, *J*= 8.2 Hz). ^{13}C-DEPTq NMR (100 MHz, CD_3OD) δ: 28.7, 34.5, 44.6, 48.7, 109.10, 109.15, 114.7, 122.2, 122.6, 125.3, 126.7, 126.8, 127.8, 127.9, 128.6, 131.5, 132.8, 139.8, 144.6, 148.4, 168.8. HR-MS m/z calcd for $C_{26}H_{26}F_3N_3O$ $[(M + H)]^+$: 454.2101; found 454.2145.

1-([1,1'-biphenyl]-4-ylmethyl)-3-(4-(piperidin-4-ylamino)-2-(trifluoromethyl)phenyl)urea (29): Final derivative 29 (217.9 mg, 36%) was synthesized from 5 as an off-white powder using the general procedure D. Precipitated from methanol/diethyl ether. ^{1}H NMR (400 MHz, CD_3OD) δ: 1.55-1.64 (m, 2H, C*H$_2$*); 2.16 (d, 1H, C*H*$_2$, *J*= 12.2 Hz); 3.09 (d, 1H, C*H*$_2$, *J*= 6.8 Hz); 3.00 (d, 1H, C*H*$_2$, *J* = 13.1 Hz); 2.98-3.03 (m, 2H, C*H$_2$*); 3.55-3.60 (m, 1H, C*H*); 4.43 (s, 2H, C*H$_2$*); 6.87 (dd, 1H, aryl, *J'*= 2.7 Hz; *J''*= 8.6 Hz); 6.91 (d, 1H, aryl, *J*= 2.6 Hz); 7.30-7.35 (m, 2H, aryl); 7.39-7.46 (m, 4H, aryl); 7.61 (t, 4H, aryl, *J*= 7.6 Hz). ^{13}C-DEPTq NMR (100 MHz, CD_3OD) δ: 29.9, 43.0, 43.3, 109.7, 115.7, 123.9,125.4, 126.5, 126.7, 126.9,127.3, 128.4, 130.7, 138.8, 139.9, 140.8, 145.6, 158. 4. HR-MS m/z calcd for $C_{26}H_{27}F_3N_4O$ $[(M + H)]^+$: 469.2210; found 469.2134.
